# Contributions of mean and shape of blood pressure distribution to worldwide trends and variations in raised blood pressure: a pooled analysis of 1018 population-based measurement studies with 88.6 million participants

**DOI:** 10.1093/ije/dyy016

**Published:** 2018-03-19

**Authors:** NCD Risk Factor Collaboration (NCD-RisC), Bin Zhou, James Bentham, Mariachiara Di Cesare, Honor Bixby, Goodarz Danaei, Kaveh Hajifathalian, Cristina Taddei, Rodrigo M Carrillo-Larco, Shirin Djalalinia, Shahab Khatibzadeh, Charles Lugero, Niloofar Peykari, Wan Zhu Zhang, James Bennett, Ver Bilano, Gretchen A Stevens, Melanie J Cowan, Leanne M Riley, Zhengming Chen, Ian R Hambleton, Rod T Jackson, Andre Pascal Kengne, Young-Ho Khang, Avula Laxmaiah, Jing Liu, Reza Malekzadeh, Hannelore K Neuhauser, Maroje Sorić, Gregor Starc, Johan Sundström, Mark Woodward, Majid Ezzati, Leandra Abarca-Gómez, Ziad A Abdeen, Niveen M Abu-Rmeileh, Benjamin Acosta-Cazares, Robert J Adams, Wichai Aekplakorn, Kaosar Afsana, Carlos A Aguilar-Salinas, Charles Agyemang, Noor Ani Ahmad, Alireza Ahmadvand, Wolfgang Ahrens, Kamel Ajlouni, Nazgul Akhtaeva, Rajaa Al-Raddadi, Mohamed M Ali, Osman Ali, Ala'a Alkerwi, Eman Aly, Deepak N Amarapurkar, Philippe Amouyel, Antoinette Amuzu, Lars Bo Andersen, Sigmund A Anderssen, Lars H Ängquist, Ranjit Mohan Anjana, Daniel Ansong, Hajer Aounallah-Skhiri, Joana Araújo, Inger Ariansen, Tahir Aris, Nimmathota Arlappa, Dominique Arveiler, Krishna K Aryal, Thor Aspelund, Felix K Assah, Maria Cecília F Assunção, Mária Avdicová, Ana Azevedo, Fereidoun Azizi, Bontha V Babu, Suhad Bahijri, Nagalla Balakrishna, Mohamed Bamoshmoosh, Maciej Banach, Piotr Bandosz, José R Banegas, Carlo M Barbagallo, Alberto Barceló, Amina Barkat, Aluisio J D Barros, Mauro V Barros, Iqbal Bata, Anwar M Batieha, Assembekov Batyrbek, Louise A Baur, Robert Beaglehole, Habiba Ben Romdhane, Mikhail Benet, Lowell S Benson, Antonio Bernabe-Ortiz, Gailute Bernotiene, Heloisa Bettiol, Aroor Bhagyalaxmi, Sumit Bharadwaj, Santosh K Bhargava, Yufang Bi, Mukharram Bikbov, Bihungum Bista, Peter Bjerregaard, Espen Bjertness, Marius B Bjertness, Cecilia Björkelund, Anneke Blokstra, Simona Bo, Martin Bobak, Heiner Boeing, Jose G Boggia, Carlos P Boissonnet, Vanina Bongard, Rossana Borchini, Pascal Bovet, Lutgart Braeckman, Imperia Brajkovich, Francesco Branca, Juergen Breckenkamp, Hermann Brenner, Lizzy M Brewster, Graziella Bruno, H B(as) Bueno-de-Mesquita, Anna Bugge, Con Burns, Michael Bursztyn, Antonio Cabrera de León, Joseph Cacciottolo, Hui Cai, Christine Cameron, Günay Can, Ana Paula C Cândido, Vincenzo Capuano, Viviane C Cardoso, Axel C Carlsson, Maria J Carvalho, Felipe F Casanueva, Juan-Pablo Casas, Carmelo A Caserta, Snehalatha Chamukuttan, Angelique W Chan, Queenie Chan, Himanshu K Chaturvedi, Nishi Chaturvedi, Chien-Jen Chen, Fangfang Chen, Huashuai Chen, Shuohua Chen, Zhengming Chen, Ching-Yu Cheng, Imane Cherkaoui Dekkaki, Angela Chetrit, Arnaud Chiolero, Shu-Ti Chiou, Adela Chirita-Emandi, María-Dolores Chirlaque, Belong Cho, Yumi Cho, Diego G Christofaro, Jerzy Chudek, Renata Cifkova, Eliza Cinteza, Frank Claessens, Els Clays, Hans Concin, Cyrus Cooper, Rachel Cooper, Tara C Coppinger, Simona Costanzo, Dominique Cottel, Chris Cowell, Cora L Craig, Ana B Crujeiras, Juan J Cruz, Graziella D'Arrigo, Eleonora d'Orsi, Jean Dallongeville, Albertino Damasceno, Goodarz Danaei, Rachel Dankner, Thomas M Dantoft, Luc Dauchet, Kairat Davletov, Guy De Backer, Dirk De Bacquer, Giovanni de Gaetano, Stefaan De Henauw, Paula Duarte de Oliveira, Delphine De Smedt, Mohan Deepa, Abbas Dehghan, Hélène Delisle, Valérie Deschamps, Klodian Dhana, Augusto F Di Castelnuovo, Juvenal Soares Dias-da-Costa, Alejandro Diaz, Ty T Dickerson, Shirin Djalalinia, Ha T P Do, Chiara Donfrancesco, Silvana P Donoso, Angela Döring, Maria Dorobantu, Kouamelan Doua, Wojciech Drygas, Virginija Dulskiene, Aleksandar Džakula, Vilnis Dzerve, Elzbieta Dziankowska-Zaborszczyk, Robert Eggertsen, Ulf Ekelund, Jalila El Ati, Paul Elliott, Roberto Elosua, Rajiv T Erasmus, Cihangir Erem, Louise Eriksen, Johan G Eriksson, Jorge Escobedo-de la Peña, Alun Evans, David Faeh, Caroline H Fall, Farshad Farzadfar, Francisco J Felix-Redondo, Trevor S Ferguson, Romulo A Fernandes, Daniel Fernández-Bergés, Daniel Ferrante, Marika Ferrari, Catterina Ferreccio, Jean Ferrieres, Joseph D Finn, Krista Fischer, Bernhard Föger, Leng Huat Foo, Ann-Sofie Forslund, Maria Forsner, Heba M Fouad, Damian K Francis, Maria do Carmo Franco, Oscar H Franco, Guillermo Frontera, Flavio D Fuchs, Sandra C Fuchs, Yuki Fujita, Takuro Furusawa, Zbigniew Gaciong, Fabio Galvano, Manoli Garcia-de-la-Hera, Dickman Gareta, Sarah P Garnett, Jean-Michel Gaspoz, Magda Gasull, Louise Gates, Johanna M Geleijnse, Anoosheh Ghasemian, Anup Ghimire, Simona Giampaoli, Francesco Gianfagna, Tiffany K Gill, Jonathan Giovannelli, Rebecca A Goldsmith, Helen Gonçalves, Marcela Gonzalez-Gross, Juan P González-Rivas, Mariano Bonet Gorbea, Frederic Gottrand, Sidsel Graff-Iversen, Dušan Grafnetter, Aneta Grajda, Maria G Grammatikopoulou, Ronald D Gregor, Tomasz Grodzicki, Anders Grøntved, Giuseppe Grosso, Gabriella Gruden, Vera Grujic, Dongfeng Gu, Ong Peng Guan, Elias F Gudmundsson, Vilmundur Gudnason, Ramiro Guerrero, Idris Guessous, Andre L Guimaraes, Martin C Gulliford, Johanna Gunnlaugsdottir, Marc Gunter, Prakash C Gupta, Rajeev Gupta, Oye Gureje, Beata Gurzkowska, Laura Gutierrez, Felix Gutzwiller, Farzad Hadaegh, Jytte Halkjær, Ian R Hambleton, Rebecca Hardy, Rachakulla Hari Kumar, Jun Hata, Alison J Hayes, Jiang He, Yuna He, Marleen Elisabeth, Ana Henriques, Leticia Hernandez Cadena, Sauli Herrala, Ramin Heshmat, Ilpo Tapani Hihtaniemi, Sai Yin Ho, Suzanne C Ho, Michael Hobbs, Albert Hofman, Gonul Horasan Dinc, Andrea R V R Horimoto, Claudia M Hormiga, Bernardo L Horta, Leila Houti, Christina Howitt, Thein Thein Htay, Aung Soe Htet, Maung Maung Than Htike, Yonghua Hu, José María Huerta, Martijn Huisman, Abdullatif S Husseini, Inge Huybrechts, Nahla Hwalla, Licia Iacoviello, Anna G Iannone, Mohsen M Ibrahim, Norazizah Ibrahim Wong, Nayu Ikeda, M Arfan Ikram, Vilma E Irazola, Muhammad Islam, Aziz al-Safi Ismail, Vanja Ivkovic, Masanori Iwasaki, Rod T Jackson, Jeremy M Jacobs, Hashem Jaddou, Tazeen Jafar, Konrad Jamrozik, Imre Janszky, Grazyna Jasienska, Ana Jelaković, Bojan Jelaković, Garry Jennings, Seung-lyeal Jeong, Chao Qiang Jiang, Michel Joffres, Mattias Johansson, Jari J Jokelainen, Jost B Jonas, Torben Jørgensen, Pradeep Joshi, Jacek Jóźwiak, Anne Juolevi, Gregor Jurak, Vesna Jureša, Rudolf Kaaks, Anthony Kafatos, Eero O Kajantie, Ofra Kalter-Leibovici, Nor Azmi Kamaruddin, Khem B Karki, Amir Kasaeian, Joanne Katz, Jussi Kauhanen, Prabhdeep Kaur, Maryam Kavousi, Gyulli Kazakbaeva, Ulrich Keil, Lital Keinan Boker, Sirkka Keinänen-Kiukaanniemi, Roya Kelishadi, Han C G Kemper, Andre P Kengne, Alina Kerimkulova, Mathilde Kersting, Timothy Key, Yousef Saleh Khader, Davood Khalili, Young-Ho Khang, Mohammad Khateeb, Kay-Tee Khaw, Ursula Kiechl-Kohlendorfer, Stefan Kiechl, Japhet Killewo, Jeongseon Kim, Yeon-Yong Kim, Jurate Klumbiene, Michael Knoflach, Elin Kolle, Patrick Kolsteren, Paul Korrovits, Seppo Koskinen, Katsuyasu Kouda, Sudhir Kowlessur, Slawomir Koziel, Susi Kriemler, Peter Lund Kristensen, Steinar Krokstad, Daan Kromhout, Herculina S Kruger, Ruzena Kubinova, Renata Kuciene, Diana Kuh, Urho M Kujala, Zbigniew Kulaga, R Krishna Kumar, Pawel Kurjata, Yadlapalli S Kusuma, Kari Kuulasmaa, Catherine Kyobutungi, Tiina Laatikainen, Carl Lachat, Tai Hing Lam, Orlando Landrove, Vera Lanska, Georg Lappas, Bagher Larijani, Lars E Laugsand, Avula Laxmaiah, Khanh Le Nguyen Bao, Tuyen D Le, Catherine Leclercq, Jeannette Lee, Jeonghee Lee, Terho Lehtimäki, Luz M León-Muñoz, Naomi S Levitt, Yanping Li, Christa L Lilly, Wei-Yen Lim, M Fernanda Lima-Costa, Hsien-Ho Lin, Xu Lin, Lars Lind, Allan Linneberg, Lauren Lissner, Mieczyslaw Litwin, Jing Liu, Roberto Lorbeer, Paulo A Lotufo, José Eugenio Lozano, Dalia Luksiene, Annamari Lundqvist, Nuno Lunet, Per Lytsy, Guansheng Ma, Jun Ma, George L L Machado-Coelho, Suka Machi, Stefania Maggi, Dianna J Magliano, Emmanuella Magriplis, Marjeta Majer, Marcia Makdisse, Reza Malekzadeh, Rahul Malhotra, Kodavanti Mallikharjuna Rao, Sofia Malyutina, Yannis Manios, Jim I Mann, Enzo Manzato, Paula Margozzini, Pedro Marques-Vidal, Larissa Pruner Marques, Jaume Marrugat, Reynaldo Martorell, Ellisiv B Mathiesen, Alicia Matijasevich, Tandi E Matsha, Jean Claude N Mbanya, Anselmo J Mc Donald Posso, Shelly R McFarlane, Stephen T McGarvey, Stela McLachlan, Rachael M McLean, Scott B McLean, Breige A McNulty, Sounnia Mediene-Benchekor, Jurate Medzioniene, Aline Meirhaeghe, Christa Meisinger, Ana Maria B Menezes, Geetha R Menon, Indrapal I Meshram, Andres Metspalu, Haakon E Meyer, Jie Mi, Kairit Mikkel, Jody C Miller, Cláudia S Minderico, Juan Francisco, J Jaime Miranda, Erkin Mirrakhimov, Marjeta Mišigoj-Durakovic, Pietro A Modesti, Mostafa K Mohamed, Kazem Mohammad, Noushin Mohammadifard, Viswanathan Mohan, Salim Mohanna, Muhammad Fadhli Mohd Yusoff, Line T Møllehave, Niels C Møller, Dénes Molnár, Amirabbas Momenan, Charles K Mondo, Kotsedi Daniel K Monyeki, Jin Soo Moon, Leila B Moreira, Alain Morejon, Luis A Moreno, Karen Morgan, George Moschonis, Malgorzata Mossakowska, Aya Mostafa, Jorge Mota, Mohammad Esmaeel Motlagh, Jorge Motta, Kelias P Msyamboza, Thet Thet Mu, Maria L Muiesan, Martina Müller-Nurasyid, Neil Murphy, Jaakko Mursu, Vera Musil, Iraj Nabipour, Gabriele Nagel, Balkish M Naidu, Harunobu Nakamura, Jana Námešná, Ei Ei K Nang, Vinay B Nangia, Sameer Narake, Matthias Nauck, Eva Maria Navarrete-Muñoz, Ndeye Coumba Ndiaye, William A Neal, Ilona Nenko, Martin Neovius, Flavio Nervi, Hannelore K Neuhauser, Chung T Nguyen, Nguyen D Nguyen, Quang Ngoc Nguyen, Quang V Nguyen, Ramfis E Nieto-Martínez, Teemu J Niiranen, Guang Ning, Toshiharu Ninomiya, Sania Nishtar, Marianna Noale, Oscar A Noboa, Ahmad Ali Noorbala, Teresa Norat, Davide Noto, Mohannad Al Nsour, Dermot O'Reilly, Eiji Oda, Glenn Oehlers, Kyungwon Oh, Kumiko Ohara, Maria Teresa A Olinto, Isabel O Oliveira, Mohd Azahadi Omar, Altan Onat, Sok King Ong, Lariane M Ono, Pedro Ordunez, Rui Ornelas, Clive Osmond, Sergej M Ostojic, Afshin Ostovar, Johanna A Otero, Kim Overvad, Ellis Owusu-Dabo, Fred Michel Paccaud, Cristina Padez, Elena Pahomova, Andrzej Pajak, Domenico Palli, Luigi Palmieri, Wen-Harn Pan, Songhomitra Panda-Jonas, Francesco Panza, Dimitrios Papandreou, Soon-Woo Park, Winsome R Parnell, Mahboubeh Parsaeian, Nikhil D Patel, Ivan Pecin, Mangesh S Pednekar, Nasheeta Peer, Petra H Peeters, Sergio Viana Peixoto, Markku Peltonen, Alexandre C Pereira, Annette Peters, Astrid Petersmann, Janina Petkeviciene, Niloofar Peykari, Son Thai Pham, Iris Pigeot, Hynek Pikhart, Aida Pilav, Lorenza Pilotto, Freda Pitakaka, Aleksandra Piwonska, Pedro Plans-Rubió, Ozren Polašek, Miquel Porta, Marileen L P Portegies, Akram Pourshams, Hossein Poustchi, Rajendra Pradeepa, Mathur Prashant, Jacqueline F Price, Jardena J Puder, Maria Puiu, Margus Punab, Radwan F Qasrawi, Mostafa Qorbani, Tran Quoc Bao, Ivana Radic, Ricardas Radisauskas, Mahfuzar Rahman, Olli Raitakari, Manu Raj, Sudha Ramachandra Rao, Ambady Ramachandran, Elisabete Ramos, Lekhraj Rampal, Sanjay Rampal, Daniel A Rangel Reina, Josep Redon, Paul Ferdinand M Reganit, Robespierre Ribeiro, Elio Riboli, Fernando Rigo, Tobias F Rinke de Wit, Raphael M Ritti-Dias, Sian M Robinson, Cynthia Robitaille, Fernando Rodríguez-Artalejo, María del Cristo Rodriguez-Perez, Laura A Rodríguez-Villamizar, Rosalba Rojas-Martinez, Dora Romaguera, Kimmo Ronkainen, Annika Rosengren, Joel G R Roy, Adolfo Rubinstein, Blanca Sandra Ruiz-Betancourt, Marcin Rutkowski, Charumathi Sabanayagam, Harshpal S Sachdev, Olfa Saidi, Sibel Sakarya, Benoit Salanave, Eduardo Salazar Martinez, Diego Salmerón, Veikko Salomaa, Jukka T Salonen, Massimo Salvetti, Jose Sánchez-Abanto, Susana Sans, Diana A Santos, Ina S Santos, Renata Nunes dos Santos, Rute Santos, Jouko L Saramies, Luis B Sardinha, Giselle Sarganas, Nizal Sarrafzadegan, Kai-Uwe Saum, Savvas Savva, Marcia Scazufca, Herman Schargrodsky, Sabine Schipf, Carsten O Schmidt, Ben Schöttker, Constance Schultsz, Aletta E Schutte, Aye Aye Sein, Abhijit Sen, Idowu O Senbanjo, Sadaf G Sepanlou, Sanjib K Sharma, Jonathan E Shaw, Kenji Shibuya, Dong Wook Shin, Youchan Shin, Khairil Si-Ramlee, Rosalynn Siantar, Abla M Sibai, Diego Augusto Santos Silva, Mary Simon, Judith Simons, Leon A Simons, Michael Sjöström, Sine Skovbjerg, Jolanta Slowikowska-Hilczer, Przemyslaw Slusarczyk, Liam Smeeth, Margaret C Smith, Marieke B Snijder, Hung-Kwan So, Eugène Sobngwi, Stefan Söderberg, Vincenzo Solfrizzi, Emily Sonestedt, Yi Song, Thorkild I A Sørensen, Maroje Soric, Charles Sossa Jérome, Aicha Soumare, Jan A Staessen, Gregor Starc, Maria G Stathopoulou, Bill Stavreski, Jostein Steene-Johannessen, Peter Stehle, Aryeh D Stein, George S Stergiou, Jochanan Stessman, Jutta Stieber, Doris Stöckl, Tanja Stocks, Jakub Stokwiszewski, Karien Stronks, Maria Wany Strufaldi, Chien-An Sun, Johan Sundström, Yn-Tz Sung, Paibul Suriyawongpaisal, Rody G Sy, E Shyong Tai, Mari-Liis Tammesoo, Abdonas Tamosiunas, Eng Joo Tan, Xun Tang, Frank Tanser, Yong Tao, Mohammed Rasoul Tarawneh, Carolina B Tarqui-Mamani, Oana-Florentina Tautu, Anne Taylor, Holger Theobald, Xenophon Theodoridis, Lutgarde Thijs, Betina H Thuesen, Anne Tjonneland, Hanna K Tolonen, Janne S Tolstrup, Murat Topbas, Roman Topór-Madry, María José Tormo, Maties Torrent, Pierre Traissac, Dimitrios Trichopoulos, Antonia Trichopoulou, Oanh T H Trinh, Atul Trivedi, Lechaba Tshepo, Marshall K Tulloch-Reid, Fikru Tullu, Tomi-Pekka Tuomainen, Jaakko Tuomilehto, Maria L Turley, Per Tynelius, Christophe Tzourio, Peter Ueda, Eunice E Ugel, Hanno Ulmer, Hannu M T Uusitalo, Gonzalo Valdivia, Damaskini Valvi, Yvonne T van der Schouw, Koen Van Herck, Hoang Van Minh, Lenie van Rossem, Natasja M Van Schoor, Irene G M van Valkengoed, Dirk Vanderschueren, Diego Vanuzzo, Lars Vatten, Tomas Vega, Gustavo Velasquez-Melendez, Giovanni Veronesi, W M Monique Verschuren, Roosmarijn Verstraeten, Cesar G Victora, Lucie Viet, Eira Viikari-Juntura, Paolo Vineis, Jesus Vioque, Jyrki K Virtanen, Sophie Visvikis-Siest, Bharathi Viswanathan, Tiina Vlasoff, Peter Vollenweider, Sari Voutilainen, Alisha N Wade, Aline Wagner, Janette Walton, Wan Mohamad Wan Bebakar, Wan Nazaimoon Wan Mohamud, Rildo S Wanderley, Ming-Dong Wang, Qian Wang, Ya Xing Wang, Ying-Wei Wang, S Goya Wannamethee, Nicholas Wareham, Niels Wedderkopp, Deepa Weerasekera, Peter H Whincup, Kurt Widhalm, Indah S Widyahening, Andrzej Wiecek, Alet H Wijga, Rainford J Wilks, Johann Willeit, Peter Willeit, Emmanuel A Williams, Tom Wilsgaard, Bogdan Wojtyniak, Roy A Wong-McClure, Justin Y Y Wong, Tien Yin Wong, Jean Woo, Mark Woodward, Aleksander Giwercman Wu, Frederick C Wu, Shouling Wu, Haiquan Xu, Weili Yan, Xiaoguang Yang, Xingwang Ye, Panayiotis K Yiallouros, Akihiro Yoshihara, Novie O Younger-Coleman, Ahmad Faudzi Yusoff, Ahmad Ali Zainuddin, Sabina Zambon, Antonis Zampelas, Tomasz Zdrojewski, Yi Zeng, Dong Zhao, Wenhua Zhao, Wei Zheng, Yingfeng Zheng, Dan Zhu, Baurzhan Zhussupov, Esther Zimmermann, Julio Zuñiga Cisneros

**Keywords:** Blood pressure, hypertension, population health, global health, non-communicable disease

## Abstract

**Background:**

Change in the prevalence of raised blood pressure could be due to both shifts in the entire distribution of blood pressure (representing the combined effects of public health interventions and secular trends) and changes in its high-blood-pressure tail (representing successful clinical interventions to control blood pressure in the hypertensive population). Our aim was to quantify the contributions of these two phenomena to the worldwide trends in the prevalence of raised blood pressure.

**Methods:**

We pooled 1018 population-based studies with blood pressure measurements on 88.6 million participants from 1985 to 2016. We first calculated mean systolic blood pressure (SBP), mean diastolic blood pressure (DBP) and prevalence of raised blood pressure by sex and 10-year age group from 20–29 years to 70–79 years in each study, taking into account complex survey design and survey sample weights, where relevant. We used a linear mixed effect model to quantify the association between (probit-transformed) prevalence of raised blood pressure and age-group- and sex-specific mean blood pressure. We calculated the contributions of change in mean SBP and DBP, and of change in the prevalence-mean association, to the change in prevalence of raised blood pressure.

**Results:**

In 2005–16, at the same level of population mean SBP and DBP, men and women in South Asia and in Central Asia, the Middle East and North Africa would have the highest prevalence of raised blood pressure, and men and women in the high-income Asia Pacific and high-income Western regions would have the lowest. In most region-sex-age groups where the prevalence of raised blood pressure declined, one half or more of the decline was due to the decline in mean blood pressure. Where prevalence of raised blood pressure has increased, the change was entirely driven by increasing mean blood pressure, offset partly by the change in the prevalence-mean association.

**Conclusions:**

Change in mean blood pressure is the main driver of the worldwide change in the prevalence of raised blood pressure, but change in the high-blood-pressure tail of the distribution has also contributed to the change in prevalence, especially in older age groups.

**Key Messages**

- After accounting for the difference in mean blood pressure, there is still a 3–5 percentage-point difference in the prevalence of raised blood pressure across regions, being highest in South Asia and in Central Asia, the Middle East and North Africa, and lowest in the high-income Asia Pacific and high-income Western regions.
- Shifts in entire distribution of blood pressure have been the main driver of the change in prevalence of raised blood pressure.
- There is also a measurable contribution from the change in the high-blood-pressure tail of the distribution, towards lowering the prevalence of raised blood pressure, especially in older people.

## Introduction

Raised blood pressure, commonly defined as systolic blood pressure (SBP) ≥ 140 mmHg or diastolic blood pressure (DBP) ≥ 90 mmHg, is used to identify individuals at high risk of cardiovascular diseases.^1–5^ Globally, one in four men and one in five women, totalling 1.13 billion adults, had raised blood pressure in 2015.^6^ One of the global non-communicable disease (NCD) targets adopted by the World Health Organization (WHO) in 2013 is to reduce the prevalence of raised blood pressure by 25% compared with its 2010 level, by 2025.^7^

The prevalence of raised blood pressure varies substantially across and within regions and countries, with age-standardized adult prevalence in 2015 ranging from 20% in the high-income Asia Pacific region to 33% in Central and Eastern Europe for men, and from 11% in the high-income Asia Pacific region to 28% in sub-Saharan Africa for women.^6^ Prevalence has declined substantially in high-income regions for decades, and is also declining in some middle-income regions; it has been stable or has increased in other low- and middle-income regions.^6^

Blood pressure is a complex trait, affected by genes, fetal and early childhood nutrition and growth,^8^ adiposity and weight gain,^9^^,^^10^ diet (especially sodium and potassium intakes),^9^^,^^11^^,^^12^ alcohol use,^10^^,^^13^ smoking,^14^ physical activity,^10^^,^^15^ air pollution,^16^ lead,^17^ noise,^18^ psychosocial stress,^19^ sleep duration^20^ and the use of blood pressure-lowering medicines. Changes in some of these factors, for example increase in body mass index (BMI) and better nutrition in childhood and adolescence, can shift the entire population distribution of blood pressure, and hence change its mean as well as the prevalence of raised blood pressure. In contrast, the use of antihypertensive medicines and lifestyle change to reduce blood pressure in those with elevated levels would reduce the prevalence of raised blood pressure by acting on the high-blood-pressure tail of the distribution, and hence change the shape of the distribution with a relatively small impact on its mean. An important question that can inform strategies for meeting the global target and reducing the burden of raised blood pressure, is to what extent regional differences and changes over time in the prevalence of raised blood pressure are driven by variations in the mean SBP and DBP versus by the shape of the distribution. We used a database of population-based studies with global coverage conducted over three decades to investigate contributions of population mean and high-blood-pressure individuals to worldwide trends and variations in raised blood pressure.

## Methods

### Study design

We first used population-based data to estimate the association between the prevalence of raised blood pressure, defined as SBP ≥ 140 mmHg or DBP ≥ 90 mmHg, and population mean SBP and DBP among men and women aged 20 to 79 years in nine regions of the world, from 1985 to 2016. We used a linear mixed effect model to quantify the association between the prevalence of raised blood pressure and mean blood pressure. Our statistical model, described below, allowed the prevalence of raised blood pressure at any level of mean SBP and DBP to differ by age group, region and time period. We then used the fitted association to estimate the contributions of changes in the population mean blood pressure versus changes in the shape of its distribution (represented by how the prevalence-mean association varied over region and time), to the changes in the prevalence of raised blood pressure in different regions.

### Data sources

We used data from NCD Risk Factor Collaboration (NCD-RisC) database, which contains studies that had measured blood pressure in representative samples of the national populations or of one or more sub-national regions and communities. NCD-RisC is a worldwide network of health researchers and practitioners whose aim is to document systematically the worldwide trends and variations in NCD risk factors. Our methods for identifying and accessing data sources, and the inclusion and exclusion criteria, are described in recent publications.^6^^,^^21–24^ In summary, the database was collated through multiple routes for identifying and accessing data. We accessed publicly available population-based multi-country and national measurement surveys as well as the WHO STEPwise approach to Surveillance (STEPS) surveys. We requested, via WHO and its regional and country offices, ministries of health and other national health and statistical agencies to identify and access population-based surveys. Requests were also sent via the World Heart Federation to its national partners. We made a similar request to the co-authors of an earlier pooled analysis of cardiometabolic risk factors,^25–28^ and invited them to re-analyse data from their studies and join NCD-RisC. To identify major sources not accessed through the above routes, we searched and reviewed published studies, and invited all eligible studies to join NCD-RisC. Finally, NCD-RisC members are periodically asked to review the list of sources from their country, to suggest additional sources currently not in the database and to verify that the included data from their country meet the inclusion criteria as listed in the Supplementary data (available at *IJE* online) and that there are no duplicates. Here, we analysed data collected from 1985 to 2016 on men and women aged 20–79 years, in 10-year age groups from 20–29 years to 70–79 years.

### Statistical methods

We first calculated mean SBP, mean DBP and prevalence of raised blood pressure for these age groups by sex in each study, taking into account complex survey design and survey sample weights, where relevant. We excluded data points which did not cover complete 10-year age groups, e.g. those in people aged 25–29 years or 60–64 years, to avoid bias in the estimated associations. We also excluded age-sex groups with < 25 participants, because their means and prevalence have larger uncertainty.

We then estimated the relationship between the prevalence of raised blood pressure and mean, using a linear mixed effect model, shown below in the equation (where *ε* is the error term), separately by sex. We used probit-transformed prevalence because it provided a better fit to the data than a simple linear model or logit transformation. The model included age group (10-year age groups from 20–29 to 70–79) and the decade when the data were collected (1985–94, 1995–2004 or 2005–16). We also included interactions between age group and mean blood pressure, between decade and mean blood pressure, and among these three terms, which allowed the prevalence-mean association to vary by age group and over time. We included regional random intercepts to account for the differences in prevalence at any level of mean SBP and DBP by region. The regions, used in previous analyses of cardiometabolic risk factors,^6^^,^^21–24^ were: Central and Eastern Europe; Central Asia, the Middle East and North Africa; East and South-east Asia; high-income Asia Pacific; high-income Western countries; Latin America and the Caribbean; Oceania; South Asia; and sub-Saharan Africa. Countries in each region are listed in Supplementary Table 1 (available as Supplementary data at *IJE* online). The models were fitted in statistical software R version 3.4.2. Goodness of fit of the models was assessed by conditional R^2^, which represents the proportion of variance explained by both fixed and random factors.^29^

$$\begin{array}{l} \text{Probit‐transformed prevalence of raised blood pressure}=\beta_{0}+\beta_{\text{1}}Mean_{SBP}+\beta_{\text{2}}Mean_{DBP}+\beta_{\text{3}}Age\_group+\beta_{\text{4}}Decade\\ +\beta_{\text{5}}Age\_group\cdot Decade+\beta_{\text{6}}Mean_{SBP}{}_{}\cdot Age\_group+\beta_{\text{7}}Mean_{DBP}\cdot Age\_group\\ +\beta_{\text{8}}Mean_{SBP}{}_{}\cdot Decade+\beta_{\text{9}}Mean_{DBP}\cdot Decade\\ +\beta_{\text{1}0}Mean_{SBP}\cdot Age\_group\cdot Decade+\beta_{\text{11}}Mean_{DBP}\cdot Age\_group\cdot Decade\\ +Random\_intercept_{Region}\;+\varepsilon \end{array}$$

We used a simulation approach to account for the uncertainty in the mean and prevalence data used in fitting the regression. Specifically, we used 1000 draws from the uncertainty distributions of each age- and sex-specific input data point (i.e. mean SBP and DBP and prevalence of raised blood pressure) with uncertainty represented by a normal distribution for mean SBP and DBP and by a binomial distribution for prevalence of raised blood pressure. We then fitted a separate regression to each of the 1000 simulated datasets. We sampled 1000 draws from the joint distribution of the regression coefficients for each of the 1000 fitted regressions (i.e. 1 000 000 sets of regression coefficients). We report the median of the resulting 1 000 000 draws for each coefficient, and their 2.5th and 97.5th percentiles as the 95% confidence interval. We also report the median of conditional R^2^ from the 1000 fitted regressions.

We used the fitted regressions to quantify how much differences across regions, and changes over time in the prevalence of raised blood pressure, were driven by differences/changes in mean SBP and DBP, versus by differences/changes in the prevalence-mean association. We first used the age-sex-specific global mean SBP and DBP in 2010 (∼mid-point of the 2005–16 period) in the fitted association, and estimated the prevalence of raised blood pressure by region. The age-sex-specific mean SBP and DBP values were taken from a recent comprehensive analysis of worldwide trends in blood pressure,^6^ and are listed in the Supplementary Table 2 (available as Supplementary data at *IJE* online). We report the differences between the predicted regional raised blood pressure prevalence and that of the world as a whole. These differences measure how much prevalence would vary across regions—due to geographical variations in the shape of blood pressure distribution—if they had the same population mean blood pressure.

**Figure 1:**
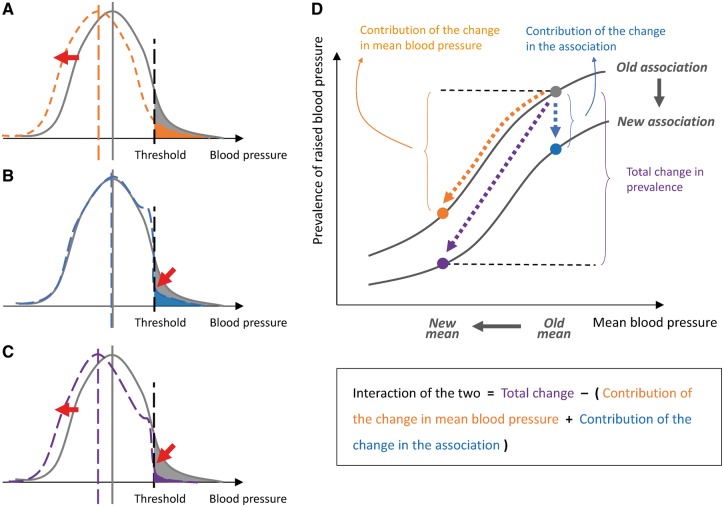
Schematic diagram for the contributions of change in mean blood pressure and in shape of the blood pressure distribution to the change in prevalence of raised blood pressure. Each S-shaped curve shows what the prevalence of raised blood pressure would be at different levels of population mean for a specific shape of population distribution. A change in mean without a change in the shape of the distribution (Panel A) would move prevalence along a curve (orange point in Panel D). A change in the shape of the distribution without a change in mean (Panel B) would vertically move prevalence from one curve to another (blue point in Panel D). The combination (Panel C) would move prevalence from one curve to another, as well as along the curve (purple point in Panel D). The figure shows the contributions when raised blood pressure is defined based on one blood pressure (either SBP or DBP). The same concept applies when raised blood pressure is defined based on both SBP and DBP.

We then decomposed total change in prevalence of raised blood pressure from 1985–94 to 2005–16 into contributions of change in mean SBP and DBP, change in the shape of prevalence-mean association and interaction of the two. The contribution of change in mean was estimated by allowing mean SBP and DBP for each age, sex and region to change over time, while keeping the decade variable fixed at 1985–94. The contribution of change in association was estimated by setting mean SBP and DBP to their 1990 levels (mid-year of 1985–94) for each age, sex and region, and allowing the decade variable to change. The interaction of the two factors is the difference between total change in prevalence and the sum of the above two components. The three components are schematically shown in Figure 1.

We repeated the above analyses for each of the 1 000 000 sets of regression coefficients. We report the median of the resulting 1 000 000 estimates as our main result and their 2.5th and 97.5th percentiles as the 95% confidence interval. All analyses were done separately for men and women. Results were calculated by 10-year age groups and then aggregated into two age bands, 20–49 years and 50–79 years, by taking the weighted average of age-specific results; weights from the WHO standard population were used.

## Results

### Data sources

**Figure 2:**
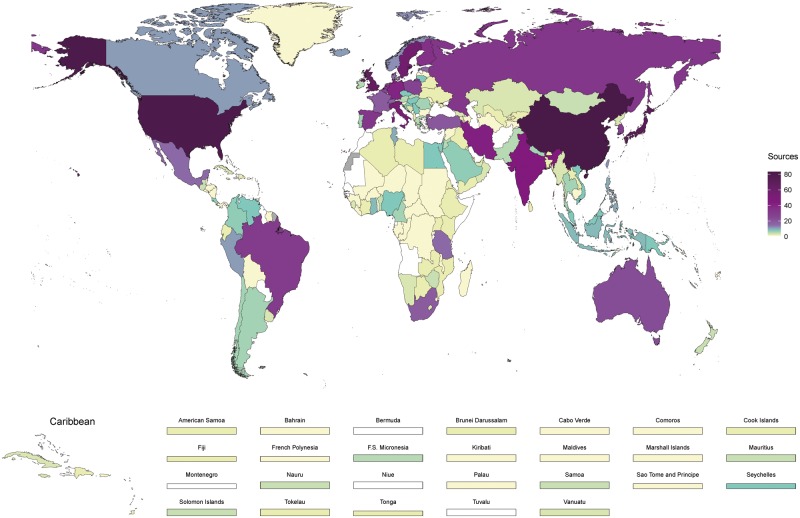
Number of blood pressure data sources from 1985 to 2016 used in the analysis, by country.

We used data from 1018 population-based studies with 88 559 656 participants, of whom 86 187 860 were aged 20–79 years, and satisfied the above inclusion criteria (Supplementary Table 3, available as Supplementary data at *IJE* online). A total of 385 studies were from the high-income Western region, 108 from East and South-east Asia, 107 from Central Asia, the Middle East and North Africa, 106 from Central and Eastern Europe, 83 from sub-Saharan Africa, 79 from Latin America and the Caribbean, 78 from high-income Asia Pacific, 38 from South Asia and 34 from Oceania. The individual-level data were summarized into 7910 age-sex-specific pairs of mean of and prevalence of raised blood pressure. The number of data sources by country is shown in Figure 2, and a list of data sources and their characteristics is provided in Supplementary Table 4 (available as Supplementary data at *IJE* online).

### Association of prevalence of raised blood pressure with mean SBP and DBP

The coefficients of the regression models are listed in Supplementary Tables 5 and 6 (available as Supplementary data at *IJE* online). Together, mean SBP and DBP, decade, age group and region explained most of the variation in the prevalence of raised blood pressure, evidenced by the high conditional R^2^ statistics of 0.918 for women and 0.871 for men.

### Changes in prevalence of raised blood pressure and mean SBP and DBP, by region

In 2005–16, the age-standardized prevalence of raised blood pressure in people aged 20–49 years ranged from 4% (95% credible interval: 3–6%) in high-income Asia Pacific to 16% (13–19%) in sub-Saharan Africa in women, and from 14% (11–17%) in high-income Asia Pacific to 25% (21–30%) in Central and Eastern Europe in men. In those aged 50–79 years, the range was from 31% (26–36%) in high-income Asia Pacific to 56% (52–61%) in sub-Saharan Africa in women, and from 40% (36–43%) in the high-income Western region to 57% (51–63%) in Central and Eastern Europe in men.

**Figure 3:**
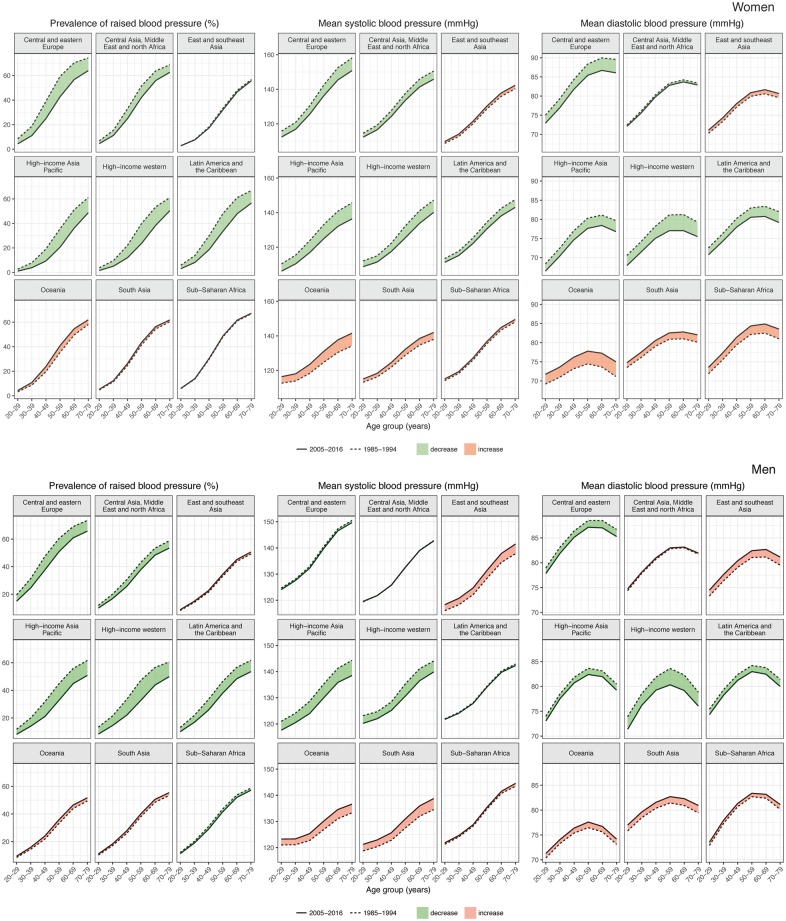
Changes in prevalence of raised blood pressure, mean SBP and mean DBP from 1985–94 to 2005–16, by region, sex and age group.

The prevalence of raised blood pressure decreased substantially from 1985–94 to 2005–16 in the two high-income regions and Central and Eastern Europe in both men and women across all ages (Figure 3).^6^ It also decreased in Latin America and the Caribbean, and in Central Asia, the Middle East and North Africa, and marginally in men in sub-Saharan Africa. Over the same period, mean SBP and mean DBP decreased in these regions and sexes, except in men in sub-Saharan Africa, whose mean SBP and DBP increased, and in men in Central Asia, the Middle East and North Africa, whose mean SBP and DBP were unchanged. The prevalence of raised blood pressure and mean SBP and DBP increased in Oceania and South Asia.

### Contributions of mean and shape of blood pressure distribution to regional variations in raised blood pressure

**Figure 4:**
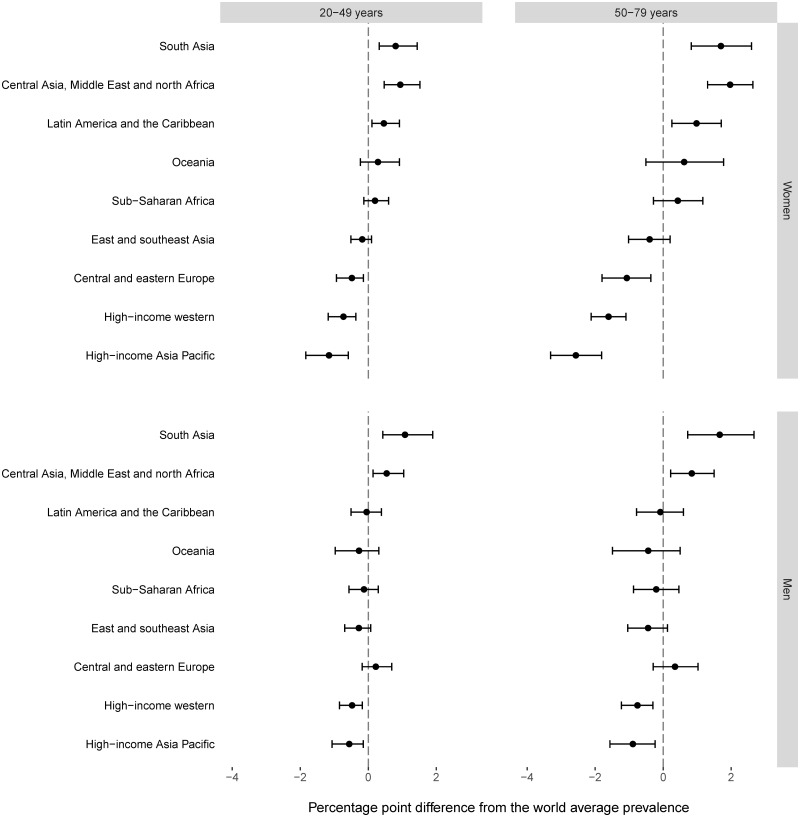
Regional differences in prevalence of raised blood pressure among men and women aged 20–49 years and 50–79 years in 2005–16 if every region had the same mean SBP and DBP, equal to the global age-sex-specific mean in 2010. The points show the central estimates and the bars their 95% confidence intervals.

Although in 2005–16 the ranking of regions in terms of prevalence of raised blood pressure was largely the same as the ranking of the mean, especially for women, inter-region differences in prevalence were not entirely due to those of mean blood pressure. Rather, some regions had an excess prevalence compared with what would be expected based on their mean, and others a lower prevalence compared with what would be expected based on their mean. At the same level of population mean SBP and DBP as that of the world as a whole, men and women in South Asia and in Central Asia, the Middle East and North Africa would have the highest prevalence of raised blood pressure, about 1–2 percentage points higher than the world average in different age groups (Figure 4). In contrast, at the same level of population mean SBP and DBP as that of the world as a whole, high-income Asia Pacific would have the lowest prevalence, followed by the high-income Western region, with prevalence about 1–3 percentage points lower than the world average across different age and sex groups, especially among women. The ordering of regions in terms of excess prevalence was similar between men and women, except for men in Central and Eastern Europe whose ranking in terms of excess prevalence was worse than that of women in the same region.

### Contributions of mean and shape of blood pressure distribution to trends in raised blood pressure

**Figure 5:**
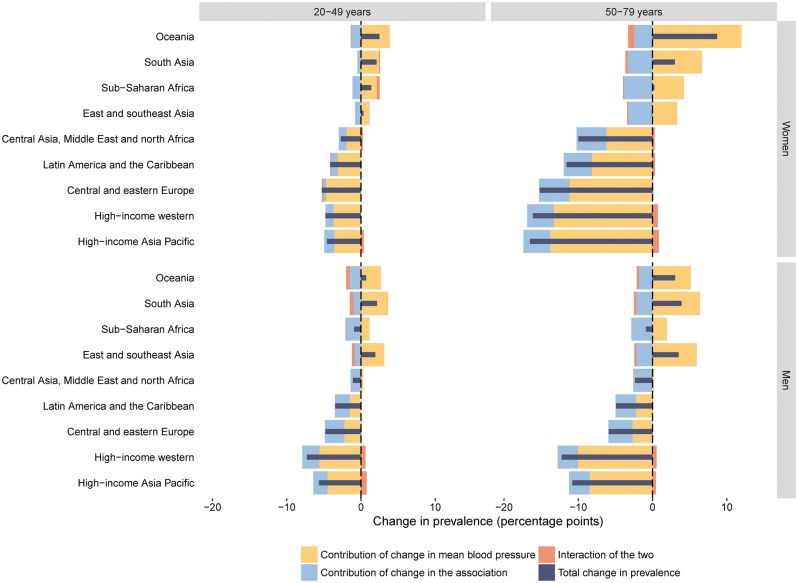
Contributions of change in mean blood pressure, change in prevalence-mean association, and the interaction of the two, to change in prevalence of raised blood pressure from 1985–94 to 2005–16 by region, sex and age group.

In most regions where sex and age groups experienced a decline in the prevalence of raised blood pressure, the decline in mean blood pressure was the main driver of the decline in prevalence (Figure 5). The main exceptions to this distributional shift were men in sub-Saharan Africa and in Central Asia, the Middle East and North Africa, whose mean blood pressure increased or remained unchanged while prevalence declined slightly. Further, in men in Latin America and the Caribbean and in Central and Eastern Europe, change in prevalence-mean association contributed marginally more to prevalence decline than did the decline in mean blood pressure. Elsewhere, the decline in mean blood pressure accounted for 60% or more of the decline in the prevalence of raised blood pressure, with a larger contribution where mean blood pressure declined more, typically in high-income regions. Change in the prevalence-mean association, which represents change in the high-blood-pressure tail of the distribution, was responsible for the majority of the remainder of change in prevalence, and for its entirety among men in sub-Saharan Africa and in Central Asia, the Middle East and North Africa. The contribution of change in prevalence-mean association was larger in those aged 50–79 years than in those aged 20–49 years in most regions, especially for women.

The prevalence of raised blood pressure increased among men and women in Oceania and South Asia, and among women in sub-Saharan Africa and men in East and South-east Asia. The increase was driven entirely by rise in mean blood pressure, offset partly by the change in the prevalence-mean association. Prevalence of raised blood pressure remained largely unchanged among women in East and South-east Asia, due to opposing effects of increasing mean and the decrease brought by the changes in prevalence-mean association.

## Discussion

We found that the trends and geographical variations in the prevalence of raised blood pressure are largely driven by shifts in the distribution of blood pressure in whole populations, rather than by the shape of the distribution. There was nonetheless an important contribution from having fewer high-blood-pressure individuals at the same level of population mean SBP and DBP over time, especially in older age groups.

Rose and Day^30^ and Laaser *et al.*^31^ used data from the Intersalt Study and from population-based studies in Germany, respectively, and found a strong association between prevalence of raised blood pressure and its mean, as we did, but neither analysis had sufficient data to quantify how the association varied in relation to age, time period or region as was done here. An analysis of data from the multi-country MONICA Project^32^ found that the upper percentiles of blood pressure distribution changed as much as its mean in some communities, and by a larger amount in others. The authors concluded that the decline in blood pressure is mostly a population phenomenon but there was no detailed quantification of the contribution, especially in relation to age, time period or region as was done here with substantially more data. Downward shifts in the whole blood pressure distribution over time have also been reported in a few high-income countries,^33–40^ with some studies also finding a larger decline in the upper tail than in the mean of the blood pressure distribution, which is consistent with our results.

The strengths of our study include presenting the first global analysis of how much population mean and high-blood-pressure individuals have contributed to worldwide trends and variations in raised blood pressure, using a large global database with data from different regions and over time, and using methods that allowed the prevalence-mean association to vary by sex, age group, time period and region. Despite using the most comprehensive global collection of population-based studies to date, some regions had limited data, especially early in our analysis period. Further, there have been changes over time in devices used for measuring blood pressure in health surveys, with standard mercury sphygmomanometers replaced by random-zero sphygmomanometers and more recently digital oscillometric devices. These changes are unlikely to have affected our regional comparisons, and would only affect prevalence-mean association over time if they had differential effects at high versus low blood pressure.

Although we found that changes in the prevalence of raised blood pressure have been mostly due to whole-distribution shifts, the behavioural, nutritional and environmental drivers of this shift remain uncertain. In high-income countries, the decline in blood pressure has occurred despite the rise in BMI,^21^ which is an established risk factor for high blood pressure, but how the concurrent and at times larger rise in BMI in low- and middle-income countries may be affecting blood pressure is unclear. Salt intake has declined in China^41^ and possibly in some high-income countries,^42–44^ but has not changed in other countries where blood pressure has declined.^45–49^ Similarly, prevalence of smoking has declined in most high-income countries and in some middle-income countries but remains high or is increasing in other low- and middle-income regions.^50^ Alcohol consumption has also had mixed trends across countries and regions.^51^ Other potential population-wide drivers of the decline in mean blood pressure which tend to improve with social and economic development include year-round availability of fruits and vegetables, which might increase the amount and regularity of their consumption;^52^ central heating at home and work which would lower winter blood pressure;^53–55^ and improvements in early childhood and adolescent nutrition, as seen in greater height in successive birth cohorts when they reach adulthood.^23^ A role for such distal determinants with life course impacts is strengthened by the fact that blood pressure is also decreasing in adolescents in high-income countries and possibly some middle-income countries.^56–60^

Whereas these determinants act to lower mean blood pressure, better developed health systems are more effective in identifying and treating high-blood-pressure individuals, which would change the tail of the distribution without a major impact on its mean. The role of treatment in reducing the prevalence of high blood pressure has become increasingly important as clinical guidelines have lowered the threshold for diagnosing and treating hypertension, e.g. from having an SBP of 160 mmHg or DBP of 95 mmHg in the 1970s^61^ to an SBP of 140 mmHg or DBP of 90 mmHg,^4^^,^^62^ and to an SBP of 130 mmHg or DBP of 80 mmHg in the newly released ACC/AHA guidelines.^3^ Over time, regional and international guidelines for diagnosis and treatment of hypertension, which are evaluated as cost-effective,^2^^,^^63^^,^^64^ have been developed and a larger share of people with raised blood pressure are treated in high-income countries^33^^,^^65–72^ and in some middle-income countries.^73–78^ Nonetheless, treatment coverage and effectiveness remain low, especially in low-income settings.^79^^,^^80^ Further, there have been improvements in effectiveness of treatment over time, leading to better control of those with hypertension. It may also be the case that changes in some risk factors, e.g. lower salt intake, have larger benefits for people whose blood pressure is high compared with those with low blood pressure,^11^ hence changing the high-blood-pressure tail of the distribution as well as its mean.

Our results demonstrate that changes in blood pressure both at the population and at the individual level have contributed to lowering raised blood pressure. What factors have spurred the former over the past few decades, however, remain largely unclear, and may be related to societal changes in nutrition, housing and health systems arising from social and economic development and technological progress. They also demonstrate the need for data that go beyond identifying the causes of low or high blood pressure, but also help measure how these factors change over time in worldwide populations. Learning about these factors would inform programmes that can help reverse the rise in the prevalence of raised blood pressure or accelerate its decline in low- and middle-income nations, where prevalence remains the highest, more effectively.

## Supplementary Data

Supplementary data are available at *IJE* online.

## Supplementary Material

Supplementary Data

## NCD Risk Factor Collaboration (NCD-RisC)

### Pooled Analysis and Writing

Bin Zhou (Imperial College London, UK); James Bentham (University of Kent, UK; Imperial College London, UK); Mariachiara Di Cesare (Middlesex University, UK); Honor Bixby (Imperial College London, UK); Goodarz Danaei (Harvard TH Chan School of Public Health, USA); Kaveh Hajifathalian (Cleveland Clinic, USA); Cristina Taddei (Imperial College London, UK); Rodrigo M Carrillo-Larco (Universidad Peruana Cayetano Heredia, Peru); Shirin Djalalinia (Tehran University of Medical Sciences, Iran; Ministry of Health and Medical Education, Iran); Shahab Khatibzadeh (Brandeis University, USA); Charles Lugero (Mulago Hospital, Uganda); Niloofar Peykari (Tehran University of Medical Sciences, Iran); Wan Zhu Zhang (Uganda Heart Institute, Uganda); James Bennett (Imperial College London, UK); Ver Bilano (Imperial College London, UK); Gretchen A Stevens (World Health Organization, Switzerland); Melanie J Cowan (World Health Organization, Switzerland); Leanne M Riley (World Health Organization, Switzerland); Zhengming Chen (University of Oxford, UK); Ian R Hambleton (The University of the West Indies, Barbados); Rod T Jackson (University of Auckland, New Zealand); Andre Pascal Kengne (South African Medical Research Council, South Africa); Young-Ho Khang (Seoul National University, Republic of Korea); Avula Laxmaiah (National Institute of Nutrition, India); Jing Liu (Capital Medical University Beijing An Zhen Hospital, China); Reza Malekzadeh (Tehran University of Medical Sciences, Iran); Hannelore K Neuhauser (Robert Koch Institute, Germany; German Center for Cardiovascular Research, Germany); Maroje Sorić (University of Zagreb, Croatia); Gregor Starc (University of Ljubljana, Slovenia); Johan Sundström (Uppsala University, Sweden); Mark Woodward (University of New South Wales, Australia; University of Oxford, UK); Majid Ezzati (Imperial College London, UK)

### Country and Regional Data(* equal contribution; listed alphabetically)

Leandra Abarca-Gómez (Caja Costarricense de Seguro Social, Costa Rica)*; Ziad A Abdeen (Al-Quds University, Palestine)*; Niveen M Abu-Rmeileh (Birzeit University, Palestine)*; Benjamin Acosta-Cazares (Instituto Mexicano del Seguro Social, Mexico)*; Robert J Adams (The University of Adelaide, Australia)*; Wichai Aekplakorn (Mahidol University, Thailand)*; Kaosar Afsana (BRAC, Bangladesh)*; Carlos A Aguilar-Salinas (Instituto Nacional de Ciencias Médicas y Nutricion, Mexico)*; Charles Agyemang (University of Amsterdam, The Netherlands)*; Noor Ani Ahmad (Ministry of Health Malaysia, Malaysia)*; Alireza Ahmadvand (Non-Communicable Diseases Research Center, Iran)*; Wolfgang Ahrens (Leibniz Institute for Prevention Research and Epidemiology - BIPS, Germany)*; Kamel Ajlouni (National Center for Diabetes and Endocrinology, Jordan)*; Nazgul Akhtaeva (Kazakh National Medical University, Kazakhstan)*; Rajaa Al-Raddadi (King Abdulaziz University, Saudi Arabia)*; Mohamed M Ali (World Health Organization, Switzerland)*; Osman Ali (Universiti Malaysia Sabah, Malaysia)*; Ala'a Alkerwi (Luxembourg Institute of Health, Luxembourg)*; Eman Aly (World Health Organization Regional Office for the Eastern Mediterranean, Egypt)*; Deepak N Amarapurkar (Bombay Hospital and Medical Research Centre, India)*; Philippe Amouyel (Lille University and Hospital, France)*; Antoinette Amuzu (London School of Hygiene & Tropical Medicine, UK)*; Lars Bo Andersen (Western Norway University of Applied Sciences, Norway)*; Sigmund A Anderssen (Norwegian School of Sport Sciences, Norway)*; Lars H Ängquist (Bispebjerg and Frederiksberg Hospitals, Denmark)*; Ranjit Mohan Anjana (Madras Diabetes Research Foundation, India)*; Daniel Ansong (Komfo Anokye Teaching Hospital, Ghana)*; Hajer Aounallah-Skhiri (National Institute of Public Health, Tunisia)*; Joana Araújo (Universidade do Porto, Portugal)*; Inger Ariansen (Norwegian Institute of Public Health, Norway)*; Tahir Aris (Ministry of Health Malaysia, Malaysia)*; Nimmathota Arlappa (National Institute of Nutrition, India)*; Dominique Arveiler (Strasbourg University and Hospital, France)*; Krishna K Aryal (Nepal Health Research Council, Nepal)*; Thor Aspelund (University of Iceland, Iceland)*; Felix K Assah (University of Yaoundé 1, Cameroon)*; Maria Cecília F Assunção (Federal University of Pelotas, Brazil)*; Mária Avdicová (Regional Authority of Public Health, Banska Bystrica, Slovakia)*; Ana Azevedo (University of Porto Medical School, Portugal)*; Fereidoun Azizi (Shahid Beheshti University of Medical Sciences, Iran)*; Bontha V Babu (Indian Council of Medical Research, India)*; Suhad Bahijri (King Abdulaziz University, Saudi Arabia)*; Nagalla Balakrishna (National Institute of Nutrition, India)*; Mohamed Bamoshmoosh (University of Science and Technology, Yemen)*; Maciej Banach (Medical University of Lodz, Poland)*; Piotr Bandosz (Medical University of Gdansk, Poland)*; José R Banegas (Universidad Autónoma de Madrid, Spain)*; Carlo M Barbagallo (University of Palermo, Italy)*; Alberto Barceló (Pan American Health Organization, USA)*; Amina Barkat (Université Mohammed V de Rabat, Morocco)*; Aluisio JD Barros (Federal University of Pelotas, Brazil)*; Mauro V Barros (University of Pernambuco, Brazil)*; Iqbal Bata (Dalhousie University, Canada)*; Anwar M Batieha (Jordan University of Science and Technology, Jordan)*; Assembekov Batyrbek (Kazakh National Medical University, Kazakhstan)*; Louise A Baur (University of Sydney, Australia)*; Robert Beaglehole (University of Auckland, New Zealand)*; Habiba Ben Romdhane (University Tunis El Manar, Tunisia)*; Mikhail Benet (CAFAM University Foundation, Colombia)*; Lowell S Benson (University of Utah School of Medicine, USA)*; Antonio Bernabe-Ortiz (Universidad Peruana Cayetano Heredia, Peru)*; Gailute Bernotiene (Lithuanian University of Health Sciences, Lithuania)*; Heloisa Bettiol (University of São Paulo, Brazil)*; Aroor Bhagyalaxmi (BJ Medical College, India)*; Sumit Bharadwaj (Chirayu Medical College, India)*; Santosh K Bhargava (SL Jain Hospital, India)*; Yufang Bi (Shanghai Jiao-Tong University School of Medicine, China)*; Mukharram Bikbov (Ufa Eye Research Institute, Russia)*; Bihungum Bista (Nepal Health Research Council, Nepal)*; Peter Bjerregaard (University of Southern Denmark, Denmark; University of Greenland, Greenland)*; Espen Bjertness (University of Oslo, Norway)*; Marius B Bjertness (University of Oslo, Norway)*; Cecilia Björkelund (University of Gothenburg, Sweden)*; Anneke Blokstra (National Institute for Public Health and the Environment, The Netherlands)*; Simona Bo (University of Turin, Italy)*; Martin Bobak (University College London, UK)*; Heiner Boeing (German Institute of Human Nutrition, Germany)*; Jose G Boggia (Universidad de la República, Uruguay)*; Carlos P Boissonnet (CEMIC, Argentina)*; Vanina Bongard (Toulouse University School of Medicine, France)*; Rossana Borchini (University Hospital of Varese, Italy)*; Pascal Bovet (Ministry of Health, Seychelles; University of Lausanne, Switzerland)*; Lutgart Braeckman (Ghent University, Belgium)*; Imperia Brajkovich (Universidad Central de Venezuela, Venezuela)*; Francesco Branca (World Health Organization, Switzerland)*; Juergen Breckenkamp (Bielefeld University, Germany)*; Hermann Brenner (German Cancer Research Center, Germany)*; Lizzy M Brewster (University of Amsterdam, The Netherlands)*; Graziella Bruno (University of Turin, Italy)*; H.B(as) Bueno-de-Mesquita (National Institute for Public Health and the Environment, The Netherlands)*; Anna Bugge (University of Southern Denmark, Denmark)*; Con Burns (Cork Institute of Technology, Ireland)*; Michael Bursztyn (Hadassah-Hebrew University Medical Center, Israel)*; Antonio Cabrera de León (Universidad de La Laguna, Spain)*; Joseph Cacciottolo (University of Malta, Malta)*; Hui Cai (Vanderbilt University, USA)*; Christine Cameron (Canadian Fitness and Lifestyle Research Institute, Canada)*; Günay Can (Istanbul University, Turkey)*; Ana Paula C Cândido (Universidade Federal de Juiz de Fora, Brazil)*; Vincenzo Capuano (Cardiologia di Mercato S. Severino, Italy)*; Viviane C Cardoso (University of São Paulo, Brazil)*; Axel C Carlsson (Karolinska Institutet, Sweden)*; Maria J Carvalho (University of Porto, Portugal)*; Felipe F Casanueva (Santiago de Compostela University, Spain)*; Juan-Pablo Casas (University College London, UK)*; Carmelo A Caserta (Associazione Calabrese di Epatologia, Italy)*; Snehalatha Chamukuttan (India Diabetes Research Foundation, India)*; Angelique W Chan (Duke-NUS Medical School, Singapore)*; Queenie Chan (Imperial College London, UK)*; Himanshu K Chaturvedi (National Institute of Medical Statistics, India)*; Nishi Chaturvedi (University College London, UK)*; Chien-Jen Chen (Academia Sinica, Taiwan)*; Fangfang Chen (Capital Institute of Pediatrics, China)*; Huashuai Chen (Duke University, USA)*; Shuohua Chen (Kailuan General Hospital, China)*; Zhengming Chen (University of Oxford, UK)*; Ching-Yu Cheng (Duke-NUS Medical School, Singapore)*; Imane Cherkaoui Dekkaki (Université Mohammed V de Rabat, Morocco)*; Angela Chetrit (The Gertner Institute for Epidemiology and Health Policy Research, Israel)*; Arnaud Chiolero (University of Bern, Switzerland)*; Shu-Ti Chiou (Ministry of Health and Welfare, Taiwan)*; Adela Chirita-Emandi (Victor Babes University of Medicine and Pharmacy Timisoara, Romania)*; María-Dolores Chirlaque (Murcia Regional Health Council, Spain)*; Belong Cho (Seoul National University College of Medicine, Republic of Korea)*; Yumi Cho (Korea Centers for Disease Control and Prevention, Republic of Korea)*; Diego G Christofaro (Universidade Estadual Paulista, Brazil)*; Jerzy Chudek (Medical University of Silesia, Poland)*; Renata Cifkova (Charles University in Prague, Czech Republic)*; Eliza Cinteza (Carol Davila University of Medicine and Pharmacy, Romania)*; Frank Claessens (Katholieke Universiteit Leuven, Belgium)*; Els Clays (Ghent University, Belgium)*; Hans Concin (Agency for Preventive and Social Medicine, Austria)*; Cyrus Cooper (University of Southampton, UK)*; Rachel Cooper (University College London, UK)*; Tara C Coppinger (Cork Institute of Technology, Ireland)*; Simona Costanzo (IRCCS Istituto Neurologico Mediterraneo Neuromed, Italy)*; Dominique Cottel (Institut Pasteur de Lille, France)*; Chris Cowell (University of Sydney, Australia)*; Cora L Craig (Canadian Fitness and Lifestyle Research Institute, Canada)*; Ana B Crujeiras (CIBEROBN, Spain)*; Juan J Cruz (Universidad Autónoma de Madrid, Spain)*; Graziella D'Arrigo (National Council of Research, Italy)*; Eleonora d'Orsi (Universidade Federal de Santa Catarina, Brazil)*; Jean Dallongeville (Institut Pasteur de Lille, France)*; Albertino Damasceno (Eduardo Mondlane University, Mozambique)*; Goodarz Danaei (Harvard TH Chan School of Public Health, USA)*; Rachel Dankner (The Gertner Institute for Epidemiology and Health Policy Research, Israel)*; Thomas M Dantoft (Bispebjerg and Frederiksberg Hospital, Denmark)*; Luc Dauchet (Lille University Hospital, France)*; Kairat Davletov (Kazakh National Medical University, Kazakhstan)*; Guy De Backer (Ghent University, Belgium)*; Dirk De Bacquer (Ghent University, Belgium)*; Giovanni de Gaetano (IRCCS Istituto Neurologico Mediterraneo Neuromed, Italy)*; Stefaan De Henauw (Ghent University, Belgium)*; Paula Duarte de Oliveira (Federal University of Pelotas, Brazil)*; Delphine De Smedt (Ghent University, Belgium)*; Mohan Deepa (Madras Diabetes Research Foundation, India)*; Abbas Dehghan (Erasmus Medical Center Rotterdam, The Netherlands)*; Hélène Delisle (University of Montreal, Canada)*; Valérie Deschamps (French Public Health Agency, France)*; Klodian Dhana (Erasmus Medical Center Rotterdam, The Netherlands)*; Augusto F Di Castelnuovo (IRCCS Istituto Neurologico Mediterraneo Neuromed, Italy)*; Juvenal Soares Dias-da-Costa (Universidade do Vale do Rio dos Sinos, Brazil)*; Alejandro Diaz (National Council of Scientific and Technical Research, Argentina)*; Ty T Dickerson (University of Utah School of Medicine, USA)*; Shirin Djalalinia (Tehran University of Medical Sciences, Iran; Ministry of Health and Medical Education, Iran)*; Ha TP Do (National Institute of Nutrition, Vietnam)*; Annette J Dobson (University of Queensland, Australia)*; Chiara Donfrancesco (Istituto Superiore di Sanità, Italy)*; Silvana P Donoso (Universidad de Cuenca, Ecuador)*; Angela Döring (Helmholtz Zentrum München, Germany)*; Maria Dorobantu (Carol Davila University of Medicine and Pharmacy, Romania)*; Kouamelan Doua (Ministère de la Santé et de la Lutte Contre le Sida, Côte d’Ivoire)*; Wojciech Drygas (The Cardinal Wyszynski Institute of Cardiology, Poland)*; Virginija Dulskiene (Lithuanian University of Health Sciences, Lithuania)*; Aleksandar Džakula (University of Zagreb, Croatia)*; Vilnis Dzerve (University of Latvia, Latvia)*; Elzbieta Dziankowska-Zaborszczyk (Medical University of Lodz, Poland)*; Robert Eggertsen (University of Gothenburg, Sweden)*; Ulf Ekelund (Norwegian School of Sport Sciences, Norway)*; Jalila El Ati (National Institute of Nutrition and Food Technology, Tunisia)*; Paul Elliott (Imperial College London, UK)*; Roberto Elosua (Institut Hospital del Mar d'Investigacions Mèdiques, Spain)*; Rajiv T Erasmus (University of Stellenbosch, South Africa)*; Cihangir Erem (Karadeniz Technical University, Turkey)*; Louise Eriksen (University of Southern Denmark, Denmark)*; Johan G Eriksson (National Institute for Health and Welfare, Finland)*; Jorge Escobedo-de la Peña (Instituto Mexicano del Seguro Social, Mexico)*; Alun Evans (Queen's University of Belfast, UK)*; David Faeh (University of Zurich, Switzerland)*; Caroline H Fall (University of Southampton, UK)*; Farshad Farzadfar (Tehran University of Medical Sciences, Iran)*; Francisco J Felix-Redondo (Centro de Salud Villanueva Norte, Spain)*; Trevor S Ferguson (The University of the West Indies, Jamaica)*; Romulo A Fernandes (Universidade Estadual Paulista, Brazil)*; Daniel Fernández-Bergés (Hospital Don Benito-Villanueva de la Serena, Spain)*; Daniel Ferrante (Ministry of Health, Argentina)*; Marika Ferrari (Council for Agricultural Research and Economics, Italy)*; Catterina Ferreccio (Pontificia Universidad Católica de Chile, Chile)*; Jean Ferrieres (Toulouse University School of Medicine, France)*; Joseph D Finn (University of Manchester, UK)*; Krista Fischer (University of Tartu, Estonia)*; Bernhard Föger (Agency for Preventive and Social Medicine, Austria)*; Leng Huat Foo (Universiti Sains Malaysia, Malaysia)*; Ann-Sofie Forslund (Umeå University, Sweden)*; Maria Forsner (Dalarna University, Sweden)*; Heba M Fouad (World Health Organization Regional Office for the Eastern Mediterranean, Egypt)*; Damian K Francis (The University of the West Indies, Jamaica)*; Maria do Carmo Franco (Federal University of São Paulo, Brazil)*; Oscar H Franco (Erasmus Medical Center Rotterdam, The Netherlands)*; Guillermo Frontera (Hospital Universitario Son Espases, Spain)*; Flavio D Fuchs (Hospital de Clinicas de Porto Alegre, Brazil)*; Sandra C Fuchs (Universidade Federal do Rio Grande do Sul, Brazil)*; Yuki Fujita (Kindai University, Japan)*; Takuro Furusawa (Kyoto University, Japan)*; Zbigniew Gaciong (Medical University of Warsaw, Poland)*; Fabio Galvano (University of Catania, Italy)*; Manoli Garcia-de-la-Hera (CIBER en Epidemiología y Salud Pública, Spain)*; Dickman Gareta (University of KwaZulu-Natal, South Africa)*; Sarah P Garnett (University of Sydney, Australia)*; Jean-Michel Gaspoz (Geneva University Hospitals, Switzerland)*; Magda Gasull (CIBER en Epidemiología y Salud Pública, Spain)*; Louise Gates (Australian Bureau of Statistics, Australia)*; Johanna M Geleijnse (Wageningen University, The Netherlands)*; Anoosheh Ghasemian (Non-Communicable Diseases Research Center, Iran)*; Anup Ghimire (B P Koirala Institute of Health Sciences, Nepal)*; Simona Giampaoli (Istituto Superiore di Sanità, Italy)*; Francesco Gianfagna (University of Insubria, Italy; IRCCS Istituto Neurologico Mediterraneo Neuromed, Italy)*; Tiffany K Gill (The University of Adelaide, Australia)*; Jonathan Giovannelli (Lille University Hospital, France)*; Rebecca A Goldsmith (Ministry of Health, Israel)*; Helen Gonçalves (Federal University of Pelotas, Brazil)*; Marcela Gonzalez-Gross (Universidad Politécnica de Madrid, Spain)*; Juan P González-Rivas (The Andes Clinic of Cardio-Metabolic Studies, Venezuela)*; Mariano Bonet Gorbea (National Institute of Hygiene, Epidemiology and Microbiology, Cuba)*; Frederic Gottrand (Université de Lille 2, France)*; Sidsel Graff-Iversen (Norwegian Institute of Public Health, Norway)*; Dušan Grafnetter (Institute for Clinical and Experimental Medicine, Czech Republic)*; Aneta Grajda (Children's Memorial Health Institute, Poland)*; Maria G Grammatikopoulou (Alexander Technological Educational Institute, Greece)*; Ronald D Gregor (Dalhousie University, Canada)*; Tomasz Grodzicki (Jagiellonian University Medical College, Poland)*; Anders Grøntved (University of Southern Denmark, Denmark)*; Giuseppe Grosso (Azienda Ospedaliera Universitaria Policlinico Vittorio Emanuele, Italy)*; Gabriella Gruden (University of Turin, Italy)*; Vera Grujic (University of Novi Sad, Serbia)*; Dongfeng Gu (National Center of Cardiovascular Diseases, China)*; Ong Peng Guan (Singapore Eye Research Institute, Singapore)*; Elias F Gudmundsson (Icelandic Heart Association, Iceland)*; Vilmundur Gudnason (University of Iceland, Iceland)*; Ramiro Guerrero (Universidad Icesi, Colombia)*; Idris Guessous (Geneva University Hospitals, Switzerland)*; Andre L Guimaraes (State University of Montes Claros, Brazil)*; Martin C Gulliford (King's College London, UK)*; Johanna Gunnlaugsdottir (Icelandic Heart Association, Iceland)*; Marc Gunter (International Agency for Research on Cancer, France)*; Prakash C Gupta (Healis-Sekhsaria Institute for Public Health, India)*; Rajeev Gupta (Eternal Heart Care Centre & Research Institute, India)*; Oye Gureje (University of Ibadan, Nigeria)*; Beata Gurzkowska (Children's Memorial Health Institute, Poland)*; Laura Gutierrez (Institute for Clinical Effectiveness and Health Policy, Argentina)*; Felix Gutzwiller (University of Zurich, Switzerland)*; Farzad Hadaegh (Shahid Beheshti University of Medical Sciences, Iran)*; Jytte Halkjær (Danish Cancer Society Research Centre, Denmark)*; Ian R Hambleton (The University of the West Indies, Barbados)*; Rebecca Hardy (University College London, UK)*; Rachakulla Hari Kumar (National Institute of Nutrition, India)*; Jun Hata (Kyushu University, Japan)*; Alison J Hayes (University of Sydney, Australia)*; Jiang He (Tulane University, USA)*; Yuna He (Chinese Center  for Disease Control and Prevention, China)*; Marleen Elisabeth Hendriks (Academic Medical Center of University of Amsterdam, The Netherlands)*; Ana Henriques (Universidade do Porto, Portugal)*; Leticia Hernandez Cadena (National Institute of Public Health, Mexico)*; Sauli Herrala (Oulu University Hospital, Finland)*; Ramin Heshmat (Chronic Diseases Research Center, Iran)*; Ilpo Tapani Hihtaniemi (Imperial College London, UK)*; Sai Yin Ho (University of Hong Kong, China)*; Suzanne C Ho (The Chinese University of Hong Kong, China)*; Michael Hobbs (University of Western Australia, Australia)*; Albert Hofman (Erasmus Medical Center Rotterdam, The Netherlands)*; Gonul Horasan Dinc (Celal Bayar University, Turkey)*; Andrea RVR Horimoto (Heart Institute, Brazil)*; Claudia M Hormiga (Fundación Oftalmológica de Santander, Colombia)*; Bernardo L Horta (Federal University of Pelotas, Brazil)*; Leila Houti (University of Oran 1, Algeria)*; Christina Howitt (The University of the West Indies, Barbados)*; Thein Thein Htay (Independent Public Health Specialist, Myanmar)*; Aung Soe Htet (Ministry of Health, Myanmar)*; Maung Maung Than Htike (Ministry of Health, Myanmar)*; Yonghua Hu (Peking University, China)*; José María Huerta (CIBER en Epidemiología y Salud Pública, Spain)*; Martijn Huisman (VU University Medical Center and VU University, The Netherlands)*; Abdullatif S Husseini (Birzeit University, Palestine)*; Inge Huybrechts (International Agency for Research on Cancer, France)*; Nahla Hwalla (American University of Beirut, Lebanon)*; Licia Iacoviello (IRCCS Istituto Neurologico Mediterraneo Neuromed, Italy; University of Insubria, Italy)*; Anna G Iannone (Cardiologia di Mercato S. Severino, Italy)*; Mohsen M Ibrahim (Cairo University, Egypt)*; Norazizah Ibrahim Wong (Ministry of Health Malaysia, Malaysia)*; Nayu Ikeda (National Institute of Health and Nutrition, Japan)*; M Arfan Ikram (Erasmus Medical Center Rotterdam, The Netherlands)*; Vilma E Irazola (Institute for Clinical Effectiveness and Health Policy, Argentina)*; Muhammad Islam (Aga Khan University, Pakistan)*; Aziz al-Safi Ismail (Universiti Sains Malaysia, Malaysia)*; Vanja Ivkovic (UHC Zagreb, Croatia)*; Masanori Iwasaki (Niigata University, Japan)*; Rod T Jackson (University of Auckland, New Zealand)*; Jeremy M Jacobs (Hadassah University Medical Center, Israel)*; Hashem Jaddou (Jordan University of Science and Technology, Jordan)*; Tazeen Jafar (Duke-NUS Medical School, Singapore)*; Konrad Jamrozik (The University of Adelaide, Australia; deceased)*; Imre Janszky (Norwegian University of Science and Technology, Norway)*; Grazyna Jasienska (Jagiellonian University Medical College, Poland)*; Ana Jelaković (UHC Zagreb, Croatia)*; Bojan Jelaković (University of Zagreb School of Medicine, Croatia)*; Garry Jennings (Heart Foundation, Australia)*; Seung-lyeal Jeong (National Health Insurance Service, Republic of Korea)*; Chao Qiang Jiang (Guangzhou 12th Hospital, China)*; Michel Joffres (Simon Fraser University, Canada)*; Mattias Johansson (International Agency for Research on Cancer, France)*; Jari J Jokelainen (Oulu University Hospital, Finland)*; Jost B Jonas (Ruprecht-Karls-University of Heidelberg, Germany)*; Torben Jørgensen (Research Centre for Prevention and Health, Denmark)*; Pradeep Joshi (World Health Organization Country Office, India)*; Jacek Jóźwiak (Czestochowa University of Technology, Poland)*; Anne Juolevi (National Institute for Health and Welfare, Finland)*; Gregor Jurak (University of Ljubljana, Slovenia)*; Vesna Jureša (University of Zagreb, Croatia)*; Rudolf Kaaks (German Cancer Research Center, Germany)*; Anthony Kafatos (University of Crete, Greece)*; Eero O Kajantie (National Institute for Health and Welfare, Finland)*; Ofra Kalter-Leibovici (The Gertner Institute for Epidemiology and Health Policy Research, Israel)*; Nor Azmi Kamaruddin (Universiti Kebangsaan Malaysia, Malaysia)*; Khem B Karki (Nepal Health Research Council, Nepal)*; Amir Kasaeian (Tehran University of Medical Sciences, Iran)*; Joanne Katz (Johns Hopkins Bloomberg School of Public Health, USA)*; Jussi Kauhanen (University of Eastern Finland, Finland)*; Prabhdeep Kaur (National Institute of Epidemiology, India)*; Maryam Kavousi (Erasmus Medical Center Rotterdam, The Netherlands)*; Gyulli Kazakbaeva (Ufa Eye Research Institute, Russia)*; Ulrich Keil (University of Münster, Germany)*; Lital Keinan Boker (Israel Center for Disease Control, Israel)*; Sirkka Keinänen-Kiukaanniemi (Oulu University Hospital, Finland)*; Roya Kelishadi (Research Institute for Primordial Prevention of Non- communicable Disease, Iran)*; Han CG Kemper (VU University Medical Center, The Netherlands)*; Andre P Kengne (South African Medical Research Council, South Africa)*; Alina Kerimkulova (Kyrgyz State Medical Academy, Kyrgyzstan)*; Mathilde Kersting (Research Institute of Child Nutrition, Germany)*; Timothy Key (University of Oxford, UK)*; Yousef Saleh Khader (Jordan University of Science and Technology, Jordan)*; Davood Khalili (Shahid Beheshti University of Medical Sciences, Iran)*; Young-Ho Khang (Seoul National University, Republic of Korea)*; Mohammad Khateeb (National Center for Diabetes and Endocrinology, Jordan)*; Kay-Tee Khaw (University of Cambridge, UK)*; Ursula Kiechl-Kohlendorfer (Medical University of Innsbruck, Austria)*; Stefan Kiechl (Medical University of Innsbruck, Austria)*; Japhet Killewo (Muhimbili University of Health and Allied Sciences, Tanzania)*; Jeongseon Kim (National Cancer Center, Republic of Korea)*; Yeon-Yong Kim (National Health Insurance Service, Republic of Korea)*; Jurate Klumbiene (Lithuanian University of Health Sciences, Lithuania)*; Michael Knoflach (Medical University of Innsbruck, Austria)*; Elin Kolle (Norwegian School of Sport Sciences, Norway)*; Patrick Kolsteren (Institute of Tropical Medicine, Belgium)*; Paul Korrovits (Tartu University Clinics, Estonia)*; Seppo Koskinen (National Institute for Health and Welfare, Finland)*; Katsuyasu Kouda (Kindai University, Japan)*; Sudhir Kowlessur (Ministry of Health and Quality of Life, Mauritius)*; Slawomir Koziel (Polish Academy of Sciences Anthropology Unit in Wroclaw, Poland)*; Susi Kriemler (University of Zürich, Switzerland)*; Peter Lund Kristensen (University of Southern Denmark, Denmark)*; Steinar Krokstad (Norwegian University of Science and Technology, Norway)*; Daan Kromhout (University of Groningen, The Netherlands)*; Herculina S Kruger (North-West University, South Africa)*; Ruzena Kubinova (National Institute of Public Health, Czech Republic)*; Renata Kuciene (Lithuanian University of Health Sciences, Lithuania)*; Diana Kuh (University College London, UK)*; Urho M Kujala (University of Jyväskylä, Finland)*; Zbigniew Kulaga (Children's Memorial Health Institute, Poland)*; R Krishna Kumar (Amrita Institute of Medical Sciences, India)*; Pawel Kurjata (The Cardinal Wyszynski Institute of Cardiology, Poland)*; Yadlapalli S Kusuma (All India Institute of Medical Sciences, India)*; Kari Kuulasmaa (National Institute for Health and Welfare, Finland)*; Catherine Kyobutungi (African Population and Health Research Center, Kenya)*; Tiina Laatikainen (National Institute for Health and Welfare, Finland)*; Carl Lachat (Ghent University, Belgium)*; Tai Hing Lam (University of Hong Kong, China)*; Orlando Landrove (Ministerio de Salud Pública, Cuba)*; Vera Lanska (Institute for Clinical and Experimental Medicine, Czech Republic)*; Georg Lappas (Sahlgrenska Academy, Sweden)*; Bagher Larijani (Endocrinology and Metabolism Research Center, Iran)*; Lars E Laugsand (Norwegian University of Science and Technology, Norway)*; Avula Laxmaiah (National Institute of Nutrition, India)*; Khanh Le Nguyen Bao (National Institute of Nutrition, Vietnam)*; Tuyen D Le (National Institute of Nutrition, Vietnam)*; Catherine Leclercq (Food and Agriculture Organization of the United Nations, Italy)*; Jeannette Lee (National University of Singapore, Singapore)*; Jeonghee Lee (National Cancer Center, Republic of Korea)*; Terho Lehtimäki (Tampere University Hospital, Finland)*; Luz M León-Muñoz (Universidad Autónoma de Madrid, Spain)*; Naomi S Levitt (University of Cape Town, South Africa)*; Yanping Li (Harvard TH Chan School of Public Health, USA)*; Christa L Lilly (West Virginia University, USA)*; Wei-Yen Lim (National University of Singapore, Singapore)*; M Fernanda Lima-Costa (Oswaldo Cruz Foundation Rene Rachou Research Institute, Brazil)*; Hsien-Ho Lin (National Taiwan University, Taiwan)*; Xu Lin (University of Chinese Academy of Sciences, China)*; Lars Lind (Uppsala University, Sweden)*; Allan Linneberg (Bispebjerg and Frederiksberg Hospital, Denmark)*; Lauren Lissner (University of Gothenburg, Sweden)*; Mieczyslaw Litwin (Children's Memorial Health Institute, Poland)*; Jing Liu (Capital Medical University Beijing An Zhen Hospital, China)*; Roberto Lorbeer (University Medicine Greifswald, Germany)*; Paulo A Lotufo (University of São Paulo, Brazil)*; José Eugenio Lozano (Consejería de Sanidad Junta de Castilla y León, Spain)*; Dalia Luksiene (Lithuanian University of Health Sciences, Lithuania)*; Annamari Lundqvist (National Institute for Health and Welfare, Finland)*; Nuno Lunet (Universidade do Porto, Portugal)*; Per Lytsy (University of Uppsala, Sweden)*; Guansheng Ma (Peking University, China)*; Jun Ma (Peking University, China)*; George LL Machado-Coelho (Universidade Federal de Ouro Preto, Brazil)*; Suka Machi (The Jikei University School of Medicine, Japan)*; Stefania Maggi (National Research Council, Italy)*; Dianna J Magliano (Baker Heart and Diabetes Institute, Australia)*; Emmanuella Magriplis (Agricultural University of Athens, Greece)*; Marjeta Majer (University of Zagreb, Croatia)*; Marcia Makdisse (Hospital Israelita Albert Einstein, Brazil)*; Reza Malekzadeh (Shiraz University of Medical Sciences, Iran)*; Rahul Malhotra (Duke-NUS Medical School, Singapore)*; Kodavanti Mallikharjuna Rao (National Institute of Nutrition, India)*; Sofia Malyutina (Institute of Internal and Preventive Medicine, Russia)*; Yannis Manios (Harokopio University, Greece)*; Jim I Mann (University of Otago, New Zealand)*; Enzo Manzato (University of Padova, Italy)*; Paula Margozzini (Pontificia Universidad Católica de Chile, Chile)*; Pedro Marques-Vidal (Lausanne University Hospital, Switzerland)*; Larissa Pruner Marques (Universidade Federal de Santa Catarina, Brazil)*; Jaume Marrugat (CIBERCV, Spain)*; Reynaldo Martorell (Emory University, USA)*; Ellisiv B Mathiesen (UiT The Arctic University of Norway, Norway)*; Alicia Matijasevich (University of São Paulo, Brazil)*; Tandi E Matsha (Cape Peninsula University of Technology, South Africa)*; Jean Claude N Mbanya (University of Yaoundé 1, Cameroon)*; Anselmo J Mc Donald Posso (Gorgas Memorial Institute of Health Studies, Panama)*; Shelly R McFarlane (The University of the West Indies, Jamaica)*; Stephen T McGarvey (Brown University, USA)*; Stela McLachlan (University of Edinburgh, UK)*; Rachael M McLean (University of Otago, New Zealand)*; Scott B McLean (Statistics Canada, Canada)*; Breige A McNulty (University College Dublin, Ireland)*; Sounnia Mediene-Benchekor (University of Oran 1, Algeria)*; Jurate Medzioniene (Lithuanian University of Health Sciences, Lithuania)*; Aline Meirhaeghe (Institut National de la Santé et de la Recherche Médicale, France)*; Christa Meisinger (Helmholtz Zentrum München, Germany)*; Ana Maria B Menezes (Federal University of Pelotas, Brazil)*; Geetha R Menon (Indian Council of Medical Research, India)*; Indrapal I Meshram (National Institute of Nutrition, India)*; Andres Metspalu (University of Tartu, Estonia)*; Haakon E Meyer (University of Oslo, Norway)*; Jie Mi (Capital Institute of Pediatrics, China)*; Kairit Mikkel (University of Tartu, Estonia)*; Jody C Miller (University of Otago, New Zealand)*; Cláudia S Minderico (Lusófona University, Portugal)*; Juan Francisco Miquel (Pontificia Universidad Católica de Chile, Chile)*; J Jaime Miranda (Universidad Peruana Cayetano Heredia, Peru)*; Erkin Mirrakhimov (Kyrgyz State Medical Academy, Kyrgyzstan)*; Marjeta Mišigoj-Durakovic (University of Zagreb, Croatia)*; Pietro A Modesti (Universita' degli Studi di Firenze, Italy)*; Mostafa K Mohamed (Ain Shams University, Egypt)*; Kazem Mohammad (Tehran University of Medical Sciences, Iran)*; Noushin Mohammadifard (Hypertension Research Center, Iran)*; Viswanathan Mohan (Madras Diabetes Research Foundation, India)*; Salim Mohanna (Universidad Peruana Cayetano Heredia, Peru)*; Muhammad Fadhli Mohd Yusoff (Ministry of Health Malaysia, Malaysia)*; Line T Møllehave (Bispebjerg and Frederiksberg Hospital, Denmark)*; Niels C Møller (University of Southern Denmark, Denmark)*; Dénes Molnár (University of Pécs, Hungary)*; Amirabbas Momenan (Shahid Beheshti University of Medical Sciences, Iran)*; Charles K Mondo (Mulago Hospital, Uganda)*; Kotsedi Daniel K Monyeki (University of Limpopo, South Africa)*; Jin Soo Moon (Seoul National University Children's Hospital, Republic of Korea)*; Leila B Moreira (Universidade Federal do Rio Grande do Sul, Brazil)*; Alain Morejon (University Medical Science, Cuba)*; Luis A Moreno (Universidad de Zaragoza, Spain)*; Karen Morgan (RCSI Dublin, Ireland)*; George Moschonis (La Trobe University, Australia)*; Malgorzata Mossakowska (International Institute of Molecular and Cell Biology, Poland)*; Aya Mostafa (Ain Shams University, Egypt)*; Jorge Mota (University of Porto, Portugal)*; Mohammad Esmaeel Motlagh (Ahvaz Jundishapur University of Medical Sciences, Iran)*; Jorge Motta (Gorgas Memorial Institute of Public Health, Panama)*; Kelias P Msyamboza (World Health Organization Country Office, Malawi)*; Thet Thet Mu (Department of Public Health, Myanmar)*; Maria L Muiesan (University of Brescia, Italy)*; Martina Müller-Nurasyid (Helmholtz Zentrum München, Germany)*; Neil Murphy (International Agency for Research on Cancer, France)*; Jaakko Mursu (University of Eastern Finland, Finland)*; Vera Musil (University of Zagreb, Croatia)*; Iraj Nabipour (Bushehr University of Medical Sciences, Iran)*; Gabriele Nagel (Ulm University, Germany)*; Balkish M Naidu (Ministry of Health Malaysia, Malaysia)*; Harunobu Nakamura (Kobe University, Japan)*; Jana Námešná (Regional Authority of Public Health, Banska Bystrica, Slovakia)*; Ei Ei K Nang (National University of Singapore, Singapore)*; Vinay B Nangia (Suraj Eye Institute, India)*; Sameer Narake (Healis-Sekhsaria Institute for Public Health, India)*; Matthias Nauck (University Medicine of Greifswald, Germany)*; Eva Maria Navarrete-Muñoz (CIBER en Epidemiología y Salud Pública, Spain)*; Ndeye Coumba Ndiaye (INSERM, France)*; William A Neal (West Virginia University, USA)*; Ilona Nenko (Jagiellonian University Medical College, Poland)*; Martin Neovius (Karolinska Institutet, Sweden)*; Flavio Nervi (Pontificia Universidad Católica de Chile, Chile)*; Hannelore K Neuhauser (Robert Koch Institute, Germany; German Center for Cardiovascular Research, Germany)*; Chung T Nguyen (National Institute of Hygiene and Epidemiology, Vietnam)*; Nguyen D Nguyen (The University of Pharmacy and Medicine of Ho Chi Minh City, Vietnam)*; Quang Ngoc Nguyen (Hanoi Medical University, Vietnam)*; Quang V Nguyen (National Hospital of Endocrinology, Vietnam)*; Ramfis E Nieto-Martínez (Miami Veterans Affairs Healthcare System, USA)*; Teemu J Niiranen (University of Turku Tyks, Finland; National Institute for Health and Welfare, Finland)*; Guang Ning (Shanghai Jiao-Tong University School of Medicine, China)*; Toshiharu Ninomiya (Kyushu University, Japan)*; Sania Nishtar (Heartfile, Pakistan)*; Marianna Noale (National Research Council, Italy)*; Oscar A Noboa (Universidad de la República, Uruguay)*; Ahmad Ali Noorbala (Tehran University of Medical Sciences, Iran)*; Teresa Norat (Imperial College London, UK)*; Davide Noto (University of Palermo, Italy)*; Mohannad Al Nsour (Eastern Mediterranean Public Health Network, Jordan)*; Dermot O'Reilly (Queen's University of Belfast, UK)*; Eiji Oda (Tachikawa General Hospital, Japan)*; Glenn Oehlers (Academic Hospital of Paramaribo, Suriname)*; Kyungwon Oh (Korea Centers for Disease Control and Prevention, Republic of Korea)*; Kumiko Ohara (Kobe University, Japan)*; Maria Teresa A Olinto (Universidade do Vale do Rio dos Sinos, Brazil)*; Isabel O Oliveira (Federal University of Pelotas, Brazil)*; Mohd Azahadi Omar (Ministry of Health Malaysia, Malaysia)*; Altan Onat (Istanbul University, Turkey)*; Sok King Ong (Ministry of Health, Brunei)*; Lariane M Ono (Universidade Federal de Santa Catarina, Brazil)*; Pedro Ordunez (Pan American Health Organization, USA)*; Rui Ornelas (University of Madeira, Portugal)*; Clive Osmond (MRC Lifecourse Epidemiology Unit, UK)*; Sergej M Ostojic (University of Novi Sad, Serbia)*; Afshin Ostovar (Bushehr University of Medical Sciences, Iran)*; Johanna A Otero (Fundación Oftalmológica de Santander, Colombia)*; Kim Overvad (Aarhus University, Denmark)*; Ellis Owusu-Dabo (Kwame Nkrumah University of Science and Technology, Ghana)*; Fred Michel Paccaud (nstitute for Social and Preventive Medicine, Switzerland)*; Cristina Padez (University of Coimbra, Portugal)*; Elena Pahomova (University of Latvia, Latvia)*; Andrzej Pajak (Jagiellonian University Medical College, Poland)*; Domenico Palli (Cancer Prevention and Research Institute, Italy)*; Luigi Palmieri (Istituto Superiore di Sanità, Italy)*; Wen-Harn Pan (Academia Sinica, Taiwan)*; Songhomitra Panda-Jonas (Ruprecht-Karls-University of Heidelberg, Germany)*; Francesco Panza (IRCCS Casa Sollievo della Sofferenza, Italy)*; Dimitrios Papandreou (Zayed University, UAE)*; Soon-Woo Park (Catholic University of Daegu, Republic of Korea)*; Winsome R Parnell (University of Otago, New Zealand)*; Mahboubeh Parsaeian (Tehran University of Medical Sciences, Iran)*; Nikhil D Patel (Jivandeep Hospital, India)*; Ivan Pecin (University of Zagreb School of Medicine, Croatia; University Hospital Centre Zagreb, Croatia)*; Mangesh S Pednekar (Healis-Sekhsaria Institute for Public Health, India)*; Nasheeta Peer (South African Medical Research Council, South Africa)*; Petra H Peeters (University Medical Center Utrecht, The Netherlands)*; Sergio Viana Peixoto (Oswaldo Cruz Foundation Rene Rachou Research Institute, Brazil)*; Markku Peltonen (National Institute for Health and Welfare, Finland)*; Alexandre C Pereira (Heart Institute, Brazil)*; Annette Peters (Helmholtz Zentrum München, Germany)*; Astrid Petersmann (University Medicine of Greifswald, Germany)*; Janina Petkeviciene (Lithuanian University of Health Sciences, Lithuania)*; Niloofar Peykari (Ministry of Health and Medical Education, Iran)*; Son Thai Pham (Vietnam National Heart Institute, Vietnam)*; Iris Pigeot (Leibniz Institute for Prevention Research and Epidemiology - BIPS, Germany)*; Hynek Pikhart (University College London, UK)*; Aida Pilav (University of Sarajevo, Bosnia and Herzegovina)*; Lorenza Pilotto (Cardiovascular Prevention Centre Udine, Italy)*; Freda Pitakaka (Ministry of Health and Medical Services, Solomon Islands)*; Aleksandra Piwonska (The Cardinal Wyszynski Institute of Cardiology, Poland)*; Pedro Plans-Rubió (Public Health Agency of Catalonia, Spain)*; Ozren Polašek (University of Split, Croatia)*; Miquel Porta (Institut Hospital del Mar d'Investigacions Mèdiques, Spain)*; Marileen LP Portegies (Erasmus Medical Center Rotterdam, The Netherlands)*; Akram Pourshams (Digestive Oncology Research Center, Iran)*; Hossein Poustchi (Digestive Disease Research Institute, Iran)*; Rajendra Pradeepa (Madras Diabetes Research Foundation, India)*; Mathur Prashant (Indian Council of Medical Research, India)*; Jacqueline F Price (University of Edinburgh, UK)*; Jardena J Puder (Lausanne University Hospital, Switzerland)*; Maria Puiu (Victor Babes University of Medicine and Pharmacy Timisoara, Romania)*; Margus Punab (Tartu University Clinics, Estonia)*; Radwan F Qasrawi (Al-Quds University, Palestine)*; Mostafa Qorbani (Alborz University of Medical Sciences, Iran)*; Tran Quoc Bao (Ministry of Health, Vietnam)*; Ivana Radic (University of Novi Sad, Serbia)*; Ricardas Radisauskas (Lithuanian University of Health Sciences, Lithuania)*; Mahfuzar Rahman (BRAC, Bangladesh)*; Olli Raitakari (University of Turku, Finland)*; Manu Raj (Amrita Institute of Medical Sciences, India)*; Sudha Ramachandra Rao (National Institute of Epidemiology, India)*; Ambady Ramachandran (India Diabetes Research Foundation, India)*; Elisabete Ramos (University of Porto Medical School, Portugal)*; Lekhraj Rampal (Universiti Putra Malaysia, Malaysia)*; Sanjay Rampal (University of Malaya, Malaysia)*; Daniel A Rangel Reina (Gorgas Memorial Institute of Health Studies, Panama)*; Josep Redon (University of Valencia, Spain)*; Paul Ferdinand M Reganit (University of the Philippines, Philippines)*; Robespierre Ribeiro (Minas Gerais State Secretariat for Health, Brazil)*; Elio Riboli (Imperial College London, UK)*; Fernando Rigo (Health Center San Agustín, Spain)*; Tobias F Rinke de Wit (PharmAccess Foundation, The Netherlands)*; Raphael M Ritti-Dias (Universidade Nove de Julho, Brazil)*; Sian M Robinson (University of Southampton, UK)*; Cynthia Robitaille (Public Health Agency of Canada, Canada)*; Fernando Rodríguez-Artalejo (Universidad Autónoma de Madrid, Spain)*; María del Cristo Rodriguez-Perez (Canarian Health Service, Spain)*; Laura A Rodríguez-Villamizar (Universidad Industrial de Santander, Colombia)*; Rosalba Rojas-Martinez (Instituto Nacional de Salud Pública, Mexico)*; Dora Romaguera (CIBEROBN, Spain)*; Kimmo Ronkainen (University of Eastern Finland, Finland)*; Annika Rosengren (University of Gothenburg, Sweden)*; Joel GR Roy (Statistics Canada, Canada)*; Adolfo Rubinstein (Institute for Clinical Effectiveness and Health Policy, Argentina)*; Blanca Sandra Ruiz-Betancourt (Instituto Mexicano del Seguro Social, Mexico)*; Marcin Rutkowski (Medical University of Gdansk, Poland)*; Charumathi Sabanayagam (Singapore Eye Research Institute, Singapore)*; Harshpal S Sachdev (Sitaram Bhartia Institute of Science and Research, India)*; Olfa Saidi (University Tunis El Manar, Tunisia)*; Sibel Sakarya (Marmara University, Turkey)*; Benoit Salanave (French Public Health Agency, France)*; Eduardo Salazar Martinez (National Institute of Public Health, Mexico)*; Diego Salmerón (CIBER de Epidemiología y Salud Pública, Spain)*; Veikko Salomaa (National Institute for Health and Welfare, Finland)*; Jukka T Salonen (University of Helsinki, Finland)*; Massimo Salvetti (University of Brescia, Italy)*; Jose Sánchez-Abanto (National Institute of Health, Peru)*; Susana Sans (Catalan Department of Health, Spain)*; Diana A Santos (Universidade de Lisboa, Portugal)*; Ina S Santos (Federal University of Pelotas, Brazil)*; Renata Nunes dos Santos (University of Sao Paulo Clinics Hospital, Brazil)*; Rute Santos (University of Porto, Portugal)*; Jouko L Saramies (South Karelia Social and Health Care District, Finland)*; Luis B Sardinha (Universidade de Lisboa, Portugal)*; Giselle Sarganas (Robert Koch Institute, Germany)*; Nizal Sarrafzadegan (Isfahan Cardiovascular Research Center, Iran)*; Kai-Uwe Saum (German Cancer Research Center, Germany)*; Savvas Savva (Research and Education Institute of Child Health, Cyprus)*; Marcia Scazufca (University of Sao Paulo Clinics Hospital, Brazil)*; Herman Schargrodsky (Hospital Italiano de Buenos Aires, Argentina)*; Sabine Schipf (University Medicine of Greifswald, Germany)*; Carsten O Schmidt (University Medicine of Greifswald, Germany)*; Ben Schöttker (German Cancer Research Center, Germany)*; Constance Schultsz (Academic Medical Center of University of Amsterdam, The Netherlands)*; Aletta E Schutte (South African Medical Research Council, South Africa; North-West University, South Africa)*; Aye Aye Sein (Ministry of Health, Myanmar)*; Abhijit Sen (Norwegian University of Science and Technology, Norway)*; Idowu O Senbanjo (Lagos State University College of Medicine, Nigeria)*; Sadaf G Sepanlou (Tehran University of Medical Sciences, Iran)*; Sanjib K Sharma (B P Koirala Institute of Health Sciences, Nepal)*; Jonathan E Shaw (Baker Heart and Diabetes Institute, Australia)*; Kenji Shibuya (The University of Tokyo, Japan)*; Dong Wook Shin (Samsung Medical Center, Republic of Korea)*; Youchan Shin (Singapore Eye Research Institute, Singapore)*; Khairil Si-Ramlee (Ministry of Health, Brunei)*; Rosalynn Siantar (Singapore Eye Research Institute, Singapore)*; Abla M Sibai (American University of Beirut, Lebanon)*; Diego Augusto Santos Silva (Federal University of Santa Catarina, Brazil)*; Mary Simon (India Diabetes Research Foundation, India)*; Judith Simons (St Vincent's Hospital, Australia)*; Leon A Simons (University of New South Wales, Australia)*; Michael Sjöström (Karolinska Institutet, Sweden)*; Sine Skovbjerg (Bispebjerg and Frederiksberg Hospital, Denmark)*; Jolanta Slowikowska-Hilczer (Medical University of Lodz, Poland)*; Przemyslaw Slusarczyk (International Institute of Molecular and Cell Biology, Poland)*; Liam Smeeth (London School of Hygiene & Tropical Medicine, UK)*; Margaret C Smith (University of Oxford, UK)*; Marieke B Snijder (Academic Medical Center Amsterdam, The Netherlands)*; Hung-Kwan So (University of Hong Kong, China)*; Eugène Sobngwi (University of Yaoundé 1, Cameroon)*; Stefan Söderberg (Umeå University, Sweden)*; Vincenzo Solfrizzi (University of Bari, Italy)*; Emily Sonestedt (Lund University, Sweden)*; Yi Song (Peking University, China)*; Thorkild IA Sørensen (University of Copenhagen, Denmark)*; Maroje Soric (University of Zagreb, Croatia)*; Charles Sossa Jérome (Institut Régional de Santé Publique, Benin)*; Aicha Soumare (University of Bordeaux, France)*; Jan A Staessen (University of Leuven, Belgium)*; Gregor Starc (University of Ljubljana, Slovenia)*; Maria G Stathopoulou (INSERM, France)*; Bill Stavreski (Heart Foundation, Australia)*; Jostein Steene-Johannessen (Norwegian School of Sport Sciences, Norway)*; Peter Stehle (Bonn University, Germany)*; Aryeh D Stein (Emory University, USA)*; George S Stergiou (Sotiria Hospital, Greece)*; Jochanan Stessman (Hadassah University Medical Center, Israel)*; Jutta Stieber (Helmholtz Zentrum München, Germany; deceased)*; Doris Stöckl (Helmholtz Zentrum München, Germany)*; Tanja Stocks (Lund University, Sweden)*; Jakub Stokwiszewski (National Institute of Public Health-National Institute of Hygiene, Poland)*; Karien Stronks (University of Amsterdam, The Netherlands)*; Maria Wany Strufaldi (Federal University of São Paulo, Brazil)*; Chien-An Sun (Fu Jen Catholic University, Taiwan)*; Johan Sundström (Uppsala University, Sweden)*; Yn-Tz Sung (The Chinese University of Hong Kong, China)*; Paibul Suriyawongpaisal (Mahidol University, Thailand)*; Rody G Sy (University of the Philippines, Philippines)*; E Shyong Tai (National University of Singapore, Singapore)*; Mari-Liis Tammesoo (University of Tartu, Estonia)*; Abdonas Tamosiunas (Lithuanian University of Health Sciences, Lithuania)*; Eng Joo Tan (University of Sydney, Australia)*; Xun Tang (Peking University, China)*; Frank Tanser (University of KwaZulu-Natal, South Africa)*; Yong Tao (Peking University, China)*; Mohammed Rasoul Tarawneh (Ministry of Health, Jordan)*; Carolina B Tarqui-Mamani (National Institute of Health, Peru)*; Oana-Florentina Tautu (Carol Davila University of Medicine and Pharmacy, Romania)*; Anne Taylor (The University of Adelaide, Australia)*; Holger Theobald (Karolinska Institutet, Sweden)*; Xenophon Theodoridis (Alexander Technological Educational Institute, Greece)*; Lutgarde Thijs (University of Leuven, Belgium)*; Betina H Thuesen (Bispebjerg and Frederiksberg Hospital, Denmark)*; Anne Tjonneland (Danish Cancer Society Research Centre, Denmark)*; Hanna K Tolonen (National Institute for Health and Welfare, Finland)*; Janne S Tolstrup (University of Southern Denmark, Denmark)*; Murat Topbas (Karadeniz Technical University, Turkey)*; Roman Topór-Madry (Jagiellonian University Medical College, Poland)*; María José Tormo (Health Service of Murcia, Spain)*; Maties Torrent (IB-SALUT Area de Salut de Menorca, Spain)*; Pierre Traissac (Institut de Recherche pour le Développement, France)*; Dimitrios Trichopoulos (Harvard TH Chan School of Public Health, USA; deceased)*; Antonia Trichopoulou (Hellenic Health Foundation, Greece)*; Oanh TH Trinh (The University of Pharmacy and Medicine of Ho Chi Minh City, Vietnam)*; Atul Trivedi (Government Medical College, India)*; Lechaba Tshepo (Sefako Makgatho Health Science University, South Africa)*; Marshall K Tulloch-Reid (The University of the West Indies, Jamaica)*; Fikru Tullu (Addis Ababa University, Ethiopia)*; Tomi-Pekka Tuomainen (University of Eastern Finland, Finland)*; Jaakko Tuomilehto (Dasman Diabetes Institute, Kuwait)*; Maria L Turley (Ministry of Health, New Zealand)*; Per Tynelius (Karolinska Institutet, Sweden)*; Christophe Tzourio (University of Bordeaux, France)*; Peter Ueda (Harvard TH Chan School of Public Health, USA)*; Eunice E Ugel (Universidad Centro-Occidental Lisandro Alvarado, Venezuela)*; Hanno Ulmer (Medical University of Innsbruck, Austria)*; Hannu MT Uusitalo (University of Tampere Tays Eye Center, Finland)*; Gonzalo Valdivia (Pontificia Universidad Católica de Chile, Chile)*; Damaskini Valvi (Harvard TH Chan School of Public Health, USA)*; Yvonne T van der Schouw (Utrecht University, The Netherlands)*; Koen Van Herck (Ghent University, Belgium)*; Hoang Van Minh (Hanoi University of Public Health, Vietnam)*; Lenie van Rossem (University Medical Center Utrecht, The Netherlands)*; Natasja M Van Schoor (Amsterdam Public Health Research Institute, The Netherlands)*; Irene GM van Valkengoed (Academic Medical Center Amsterdam, The Netherlands)*; Dirk Vanderschueren (Katholieke Universiteit Leuven, Belgium)*; Diego Vanuzzo (Cardiovascular Prevention Centre Udine, Italy)*; Lars Vatten (Norwegian University of Science and Technology, Norway)*; Tomas Vega (Consejería de Sanidad Junta de Castilla y León, Spain)*; Gustavo Velasquez-Melendez (Universidade Federal de Minas Gerais, Brazil)*; Giovanni Veronesi (University of Insubria, Italy)*; WM Monique Verschuren (National Institute for Public Health and the Environment, The Netherlands)*; Roosmarijn Verstraeten (Institute of Tropical Medicine, Belgium)*; Cesar G Victora (Federal University of Pelotas, Brazil)*; Lucie Viet (National Institute for Public Health and the Environment, The Netherlands)*; Eira Viikari-Juntura (Finnish Institute of Occupational Health, Finland)*; Paolo Vineis (Imperial College London, UK)*; Jesus Vioque (Universidad Miguel Hernandez, Spain)*; Jyrki K Virtanen (University of Eastern Finland, Finland)*; Sophie Visvikis-Siest (INSERM, France)*; Bharathi Viswanathan (Ministry of Health, Seychelles)*; Tiina Vlasoff (North Karelian Center for Public Health, Finland)*; Peter Vollenweider (Lausanne University Hospital, Switzerland)*; Sari Voutilainen (University of Eastern Finland, Finland)*; Alisha N Wade (University of the Witwatersrand, South Africa)*; Aline Wagner (University of Strasbourg, France)*; Janette Walton (Cork Institute of Technology, Ireland)*; Wan Mohamad Wan Bebakar (Universiti Sains Malaysia, Malaysia)*; Wan Nazaimoon Wan Mohamud (Institute for Medical Research, Malaysia)*; Rildo S Wanderley Jr. (University of Pernambuco, Brazil)*; Ming-Dong Wang (Public Health Agency of Canada, Canada)*; Qian Wang (Xinjiang Medical University, China)*; Ya Xing Wang (Capital Medical University, China)*; Ying-Wei Wang (Ministry of Health and Welfare, Taiwan)*; S Goya Wannamethee (University College London, UK)*; Nicholas Wareham (University of Cambridge, UK)*; Niels Wedderkopp (University of Southern Denmark, Denmark)*; Deepa Weerasekera (Ministry of Health, New Zealand)*; Peter H Whincup (St George’s, University of London, UK)*; Kurt Widhalm (Medical University of Vienna, Austria)*; Indah S Widyahening (Universitas Indonesia, Indonesia)*; Andrzej Wiecek (Medical University of Silesia, Poland)*; Alet H Wijga (National Institute for Public Health and the Environment, The Netherlands)*; Rainford J Wilks (The University of the West Indies, Jamaica)*; Johann Willeit (Medical University of Innsbruck, Austria)*; Peter Willeit (Medical University of Innsbruck, Austria; University of Cambridge, UK)*; Emmanuel A Williams (Komfo Anokye Teaching Hospital, Ghana)*; Tom Wilsgaard (UiT The Arctic University of Norway, Norway)*; Bogdan Wojtyniak (National Institute of Public Health-National Institute of Hygiene, Poland)*; Roy A Wong-McClure (Caja Costarricense de Seguro Social, Costa Rica)*; Justin YY Wong (Ministry of Health, Brunei)*; Tien Yin Wong (Duke-NUS Medical School, Singapore)*; Jean Woo (The Chinese University of Hong Kong, China)*; Mark Woodward (University of New South Wales, Australia; University of Oxford, UK)*; Aleksander Giwercman Wu (Lund University, Sweden)*; Frederick C Wu (University of Manchester, UK)*; Shouling Wu (Kailuan General Hospital, China)*; Haiquan Xu (Institute of Food and Nutrition Development of Ministry of Agriculture, China)*; Weili Yan (Children's Hospital of Fudan University, China)*; Xiaoguang Yang (Chinese Center for Disease Control and Prevention, China)*; Xingwang Ye (University of Chinese Academy of Sciences, China)*; Panayiotis K Yiallouros (University of Cyprus, Cyprus)*; Akihiro Yoshihara (Niigata University, Japan)*; Novie O Younger-Coleman (The University of the West Indies, Jamaica)*; Ahmad Faudzi Yusoff (Ministry of Health Malaysia, Malaysia)*; Ahmad Ali Zainuddin (Universiti Teknologi MARA, Malaysia)*; Sabina Zambon (University of Padova, Italy)*; Antonis Zampelas (Agricultural University of Athens, Greece)*; Tomasz Zdrojewski (Medical University of Gdansk, Poland)*; Yi Zeng (Duke University, USA; Peking University, China, USA)*; Dong Zhao (Capital Medical University Beijing An Zhen Hospital, China)*; Wenhua Zhao (Chinese Center for Disease Control and Prevention, China)*; Wei Zheng (Vanderbilt University, USA)*; Yingfeng Zheng (Singapore Eye Research Institute, Singapore)*; Dan Zhu (Inner Mongolia Medical University, China)*; Baurzhan Zhussupov (Kazakh National Medical University, Kazakhstan)*; Esther Zimmermann (Bispebjerg and Frederiksberg Hospitals, Denmark)*; Julio Zuñiga Cisneros (Gorgas Memorial Institute of Public Health, Panama)*
