# Supplementary material for: Contributions of mean and shape of blood pressure distribution to worldwide trends and variations in raised blood pressure: a pooled analysis of 1018 population-based measurement studies with 88.6 million participants
Source: Int J Epidemiol. 2018 Mar 19;47(3):872–883i. doi: 10.1093/ije/dyy016 (PMC6005056; doi:10.1093/ije/dyy016)
Supplement: Supplementary Data [file dyy016_ije-2017-08-0945_supplementary_data_corrected.pdf]

Supplementary Data

Contributions of mean and shape of blood pressure distribution to global trends and variations in raised blood pressure: a pooled analysis of 1,018 population-based measurement studies with 88.6 million participants

NCD Risk Factor Collaboration (NCD-RisC)

Table of Contents

Supplement 1. Inclusion and exclusion criteria. 3

Supplementary Table 1. List of analysis regions and countries in each region. 5

Supplementary Table 2. Mean systolic and diastolic blood pressure by sex, age, and region. Numbers in parentheses are the 95% credible intervals. 6

Supplementary Table 3. Number of studies and data points used in the analysis, by region. 13

Supplementary Table 4. List of data sources used in the analysis and their characteristics. 14

Supplementary Table 5. Coefficients of the regression of the probit-transformed prevalence of raised blood pressure on mean systolic and diastolic blood pressure for women. 36

Supplementary Table 6. Coefficients of the regression of the probit-transformed prevalence of raised blood pressure on mean systolic and diastolic blood pressure for men. 38

References 40

Supplement 1. Inclusion and exclusion criteria.

Data sources were included in NCD-RisC database if:

- measured data on systolic blood pressure (SBP) and/or diastolic blood pressure (DBP) were available;
- study participants were ten years of age and older;
- data were collected using a probabilistic sampling method with a defined sampling frame;
- data were from population samples at the national, sub-national, or community level;
- data were collected in or after 1950; and
- data were from the countries and territories listed in Supplementary Table 2.

We excluded all data sources that included only hypertension diagnosis history or medication status without measurement of blood pressure. We also excluded data sources on population subgroups whose blood pressure levels may differ systematically from the general population, including:

- studies that had included or excluded people based on their health status or cardiovascular risk;
- studies whose participants were only ethnic minorities;
- specific educational, occupational, or socioeconomic subgroups, with the exception noted below;
- those recruited through health facilities, with the exception noted below; and
- women aged 15-19 years in surveys which sampled only ever-married women or measured height and weight only among mothers.

We used school-based data in countries, and in age-sex groups, with school enrolment of 70% or higher. We used data whose sampling frame was health insurance schemes in countries where at least 80% of the population were insured. Finally, we used data collected through general practice and primary care system in high-income countries with universal insurance, because contact with the primary care systems tends to be at least as good as response rates for population-based surveys.

Supplementary Table 1. List of analysis regions and countries in each region.

Region	Countries
**Sub-Saharan Africa (48)**	Angola, Benin, Botswana, Burkina Faso, Burundi, Cabo Verde, Cameroon, Central African Republic, Chad, Comoros, Congo, Cote d'Ivoire, Djibouti, DR Congo, Eritrea, Ethiopia, Equatorial Guinea, Gabon, Gambia, Ghana, Guinea, Guinea Bissau, Kenya, Lesotho, Liberia, Madagascar, Malawi, Mali, Mauritania, Mauritius, Mozambique, Namibia, Niger, Nigeria, Rwanda, Sao Tome and Principe, Senegal, Seychelles, Sierra Leone, Somalia, South Africa, Sudan, Swaziland, Tanzania, Togo, Uganda, Zambia, Zimbabwe
**Central Asia, Middle East and north Africa (28)**	Algeria, Armenia, Azerbaijan, Bahrain, Egypt, Georgia, Iran, Iraq, Jordan, Kazakhstan, Kuwait, Kyrgyzstan, Lebanon, Libya, Morocco, Mongolia, Occupied Palestinian Territory, Oman, Qatar, Saudi Arabia, Syrian Arab Republic, Tajikistan, Tunisia, Turkey, Turkmenistan, United Arab Emirates, Uzbekistan, Yemen
**South Asia (6)**	Afghanistan, Bangladesh, Bhutan, India, Nepal, Pakistan
**East and southeast Asia (16)**	Brunei Darussalam, Cambodia, China, China (Hong Kong SAR), Indonesia, Lao PDR, Malaysia, Maldives, Myanmar, North Korea, Philippines, Sri Lanka, Taiwan, Thailand, Timor-Leste, Viet Nam
**Oceania (17)**	American Samoa, Cook Islands, Fiji, French Polynesia, Kiribati, Marshall Islands, Micronesia (Federated States of), Nauru, Niue, Palau, Papua New Guinea, Samoa, Solomon Islands, Tokelau, Tonga, Tuvalu, Vanuatu
**High-income Asia Pacific (3)**	Japan, Singapore, South Korea
**Latin America and the Caribbean (35)**	Antigua and Barbuda, Argentina, Bahamas, Barbados, Belize, Bermuda, Bolivia, Brazil, Chile, Cuba, Colombia, Costa Rica, Dominica, Dominican Republic, Ecuador, El Salvador, Grenada, Guatemala, Guyana, Haiti, Honduras, Jamaica, Mexico, Nicaragua, Panama, Paraguay, Peru, Puerto Rico, Saint Kitts and Nevis, Saint Lucia, Saint Vincent and the Grenadines, Suriname, Trinidad and Tobago, Uruguay, Venezuela
**High-income western (27)**	Andorra, Austria, Australia, Belgium, Canada, Cyprus, Denmark, Finland, France, Germany, Greece, Greenland, Iceland, Ireland, Israel, Italy, Luxembourg, Malta, Netherlands, New Zealand, Norway, Portugal, Spain, Sweden, Switzerland, United Kingdom, United States of America
**Central and eastern Europe (20)**	Albania, Belarus, Bosnia and Herzegovina, Bulgaria, Croatia, Czech Republic, Estonia, Hungary, Latvia, Lithuania, Macedonia (TFYR), Moldova, Montenegro, Poland, Russian Federation, Romania, Serbia, Slovakia, Slovenia, Ukraine

Supplementary Table 2. Mean systolic and diastolic blood pressure by sex, age, and region. Numbers in parentheses are the 95% credible intervals.

Mean systolic blood pressure (mmHg)			
**Super-region (Men)**	**Year**		
	**1985-1994**	**1995-2004**	**2005-2016**
**20-29 years**			
World	119.2 (118.3-120.1)	119.6 (119.0-120.1)	120.3 (119.7-120.9)
Central and eastern Europe	124.6 (123.3-126.0)	123.9 (122.4-125.3)	124.1 (122.4-125.8)
Central Asia, Middle East and north Africa	119.4 (117.5-121.2)	119.1 (117.9-120.3)	119.6 (118.4-120.8)
East and southeast Asia	116.0 (114.9-117.2)	117.1 (116.0-118.0)	118.3 (117.1-119.5)
High-income Asia Pacific	121.0 (119.6-122.3)	119.4 (118.3-120.4)	117.6 (116.5-118.7)
High-income western	123.2 (122.3-124.0)	121.3 (120.6-122.1)	120.2 (119.3-121.2)
Latin America and Caribbean	121.9 (120.2-123.7)	121.2 (120.1-122.4)	121.7 (120.6-122.9)
Oceania	121.0 (118.5-123.7)	122.0 (119.1-124.9)	123.2 (119.9-126.6)
South Asia	118.7 (116.3-121.0)	119.9 (118.3-121.4)	121.2 (119.9-122.6)
Sub-Saharan Africa	121.3 (119.7-122.9)	121.6 (120.3-122.9)	121.8 (120.6-123.1)
**30-39 years**			
World	121.7 (120.8-122.6)	121.7 (121.1-122.3)	122.5 (121.8-123.1)
Central and eastern Europe	128.1 (126.7-129.4)	127.2 (125.7-128.6)	127.6 (125.9-129.2)
Central Asia, Middle East and north Africa	121.8 (119.9-123.6)	121.5 (120.4-122.7)	121.8 (120.6-122.9)
East and southeast Asia	118.3 (117.1-119.5)	119.3 (118.3-120.3)	120.7 (119.5-122.0)
High-income Asia Pacific	124.1 (122.7-125.4)	122.2 (121.1-123.3)	120.5 (119.3-121.7)
High-income western	124.6 (123.7-125.6)	123.3 (122.5-124.0)	122.0 (121.1-123.0)
Latin America and Caribbean	124.4 (122.6-126.3)	123.6 (122.5-124.8)	124.1 (123.0-125.4)
Oceania	121.1 (118.5-123.7)	122.2 (119.3-125.0)	123.3 (119.9-126.8)
South Asia	120.4 (118.0-122.8)	121.6 (119.9-123.1)	122.9 (121.5-124.4)
Sub-Saharan Africa	124.1 (122.5-125.8)	124.5 (123.1-125.8)	124.6 (123.4-125.9)
**40-49 years**			
World	125.5 (124.6-126.5)	125.4 (124.7-126.0)	125.9 (125.2-126.6)
Central and eastern Europe	132.9 (131.4-134.3)	131.9 (130.3-133.4)	132.2 (130.3-134.1)
Central Asia, Middle East and north Africa	125.7 (123.6-127.7)	125.5 (124.2-126.8)	125.8 (124.6-127.0)
East and southeast Asia	122.2 (120.8-123.4)	123.2 (122.1-124.3)	124.7 (123.3-126.1)
High-income Asia Pacific	128.6 (127.2-129.9)	126.1 (124.9-127.3)	124.0 (122.6-125.2)
High-income western	128.4 (127.4-129.4)	126.2 (125.3-127.0)	125.2 (124.1-126.2)
Latin America and Caribbean	128.1 (126.1-130.1)	127.3 (126.0-128.6)	127.9 (126.6-129.2)
Oceania	122.7 (119.8-125.6)	124.0 (120.9-127.1)	125.3 (121.7-129.0)
South Asia	122.8 (120.1-125.4)	124.2 (122.5-125.9)	125.6 (124.1-127.2)
Sub-Saharan Africa	128.2 (126.4-130.0)	128.6 (127.1-130.1)	128.7 (127.4-130.1)
**50-59 years**			
World	132.1 (130.9-133.1)	131.8 (131-132.5.0)	132.3 (131.5-133.1)
Central and eastern Europe	140.5 (138.7-142.2)	139.5 (137.7-141.2)	139.8 (137.5-142.0)
Central Asia, Middle East and north Africa	132.6 (130.1-134.9)	132.2 (130.7-133.7)	132.7 (131.2-134.1)
East and southeast Asia	128.5 (127.0-130.1)	129.8 (128.5-131.1)	131.5 (129.9-133.2)
High-income Asia Pacific	135.2 (133.6-136.8)	132.6 (131.2-134.1)	130.0 (128.4-131.5)
High-income western	135.4 (134.2-136.5)	132.6 (131.7-133.6)	130.7 (129.5-132.0)
Latin America and Caribbean	134.5 (132.1-136.8)	133.5 (132.0-135.0)	134.2 (132.7-135.8)
Oceania	126.9 (123.5-130.4)	128.4 (124.6-132.3)	130.1 (125.9-134.3)

Mean systolic blood pressure (mmHg)			
Super-region (Men)	Year		
	1985-1994	1995-2004	2005-2016
South Asia	127.5 (124.3-130.6)	129.2 (127.1-131.1)	131.0 (129.2-132.9)
Sub-Saharan Africa	134.9 (132.7-137.1)	135.4 (133.6-137.1)	135.5 (133.9-137.1)
**60-69 years**			
World	138.0 (136.8-139.3)	137.8 (137.0-138.6)	138.1 (137.2-139.0)
Central and eastern Europe	147.3 (145.2-149.3)	146.0 (143.8-148.1)	146.6 (144.2-148.9)
Central Asia, Middle East and north Africa	139.0 (136.1-141.7)	138.7 (137.0-140.4)	139.0 (137.4-140.6)
East and southeast Asia	134.5 (132.7-136.2)	135.9 (134.4-137.4)	137.9 (135.9-139.8)
High-income Asia Pacific	141.3 (139.5-143.0)	138.1 (136.5-139.7)	135.9 (133.9-137.7)
High-income western	141.1 (139.8-142.4)	139.0 (137.9-140.0)	136.5 (135.1-137.9)
Latin America and Caribbean	140.2 (137.5-142.9)	139.0 (137.3-140.7)	139.8 (138.0-141.6)
Oceania	131.1 (127.0-135.3)	132.6 (128.2-137.2)	134.5 (129.6-139.4)
South Asia	132.0 (128.3-135.5)	133.9 (131.5-136.1)	135.9 (133.8-138.0)
Sub-Saharan Africa	140.7 (138.2-143.2)	141.3 (139.2-143.2)	141.5 (139.6-143.4)
**70-79 years**			
World	141.1 (139.8-142.4)	141.1 (140.2-142.0)	141.4 (140.4-142.4)
Central and eastern Europe	150.5 (148.1-152.8)	149.2 (146.8-151.6)	149.7 (147.0-152.5)
Central Asia, Middle East and north Africa	142.8 (139.7-145.8)	142.5 (140.4-144.4)	142.7 (140.9-144.5)
East and southeast Asia	137.8 (135.9-139.7)	139.4 (137.7-141.0)	141.5 (139.3-143.6)
High-income Asia Pacific	144.3 (142.4-146.1)	141.0 (139.2-142.8)	138.5 (136.3-140.6)
High-income western	144.0 (142.6-145.5)	141.9 (140.8-143.1)	139.9 (138.4-141.4)
Latin America and Caribbean	142.9 (139.9-145.8)	141.6 (139.7-143.6)	142.3 (140.4-144.3)
Oceania	133.3 (128.5-138.3)	134.7 (129.6-139.9)	136.6 (130.9-142.2)
South Asia	134.5 (130.6-138.4)	136.6 (133.9-139.1)	138.7 (136.5-141.1)
Sub-Saharan Africa	143.5 (140.7-146.3)	144.1 (141.8-146.3)	144.5 (142.4-146.7)

Mean systolic blood pressure (mmHg)			
Super-region (Women)	Year		
	1985-1994	1995-2004	2005-2016
**20-29 years**			
World	111.8 (111.0-112.5)	111.8 (111.3-112.3)	112.0 (111.5-112.6)
Central and eastern Europe	115.8 (114.6-117.0)	114.0 (112.8-115.3)	112.4 (110.9-113.9)
Central Asia, Middle East and north Africa	114.6 (113.0-116.2)	113.6 (112.5-114.8)	112.4 (111.3-113.4)
East and southeast Asia	108.7 (107.7-109.7)	109.0 (108.2-109.8)	109.8 (108.8-110.8)
High-income Asia Pacific	110.3 (109.2-111.5)	108.3 (107.4-109.2)	106.2 (105.2-107.1)
High-income western	112.1 (111.3-112.8)	110.2 (109.6-110.8)	108.7 (107.9-109.5)
Latin America and Caribbean	113.4 (111.8-114.9)	111.9 (110.9-112.8)	111.2 (110.3-112.2)
Oceania	112.7 (110.3-115.0)	114.5 (111.8-117.1)	116.4 (113.4-119.6)
South Asia	113.2 (111.2-115.2)	114.2 (112.9-115.6)	115.0 (113.8-116.2)
Sub-Saharan Africa	114.1 (112.7-115.6)	114.8 (113.7-116.0)	115.1 (114.0-116.1)
**30-39 years**			
World	115.7 (114.9-116.6)	115.3 (114.8-115.9)	115.6 (115.0-116.2)
Central and eastern Europe	120.9 (119.6-122.2)	118.8 (117.4-120.3)	116.9 (115.3-118.6)
Central Asia, Middle East and north Africa	119.3 (117.5-121.0)	118.2 (117.1-119.4)	116.6 (115.5-117.7)
East and southeast Asia	112.7 (111.6-113.9)	113.0 (112.0-113.9)	114.0 (112.8-115.2)
High-income Asia Pacific	115.4 (114.1-116.7)	112.8 (111.8-113.8)	110.4 (109.3-111.5)
High-income western	115.0 (114.2-115.9)	113.3 (112.6-113.9)	111.4 (110.6-112.3)

Mean systolic blood pressure (mmHg)			
**Super-region (Women)**	**Year**		
	**1985-1994**	**1995-2004**	**2005-2016**
Latin America and Caribbean	117.7 (115.9-119.5)	115.9 (114.8-117.0)	115.2 (114.1-116.4)
Oceania	113.9 (111.4-116.4)	115.9 (113.0-118.7)	118.1 (114.7-121.7)
South Asia	116.3 (113.9-118.5)	117.5 (116.0-118.9)	118.3 (117.1-119.6)
Sub-Saharan Africa	118.3 (116.7-119.9)	119.1 (117.9-120.4)	119.3 (118.2-120.5)
**40-49 years**			
World	123.1 (122.1-124.1)	122.4 (121.7-123.0)	122.3 (121.5-123.0)
Central and eastern Europe	130.5 (128.9-132.1)	127.9 (126.2-129.7)	125.6 (123.5-127.7)
Central Asia, Middle East and north Africa	127.7 (125.5-129.8)	126.3 (125.0-127.7)	124.4 (123.0-125.7)
East and southeast Asia	120.0 (118.6-121.4)	120.1 (119.0-121.3)	121.4 (119.8-122.9)
High-income Asia Pacific	123.8 (122.4-125.2)	120.2 (119.0-121.4)	116.9 (115.6-118.2)
High-income western	122.2 (121.2-123.3)	119.5 (118.7-120.3)	117.5 (116.4-118.6)
Latin America and Caribbean	125.2 (123.0-127.4)	123.0 (121.6-124.3)	122.1 (120.7-123.5)
Oceania	118.4 (115.3-121.5)	120.8 (117.5-124.2)	123.5 (119.6-127.9)
South Asia	121.9 (119.0-124.7)	123.5 (121.7-125.3)	124.6 (123.0-126.1)
Sub-Saharan Africa	126.0 (123.9-128.0)	126.9 (125.4-128.5)	127.1 (125.7-128.5)
**50-59 years**			
World	132.5 (131.3-133.7)	131.2 (130.4-131.9)	130.9 (130.0-131.7)
Central and eastern Europe	142.5 (140.5-144.6)	139.2 (137.1-141.3)	136.4 (133.8-139.0)
Central Asia, Middle East and north Africa	137.7 (135.0-140.4)	136.2 (134.5-137.9)	133.7 (132.0-135.3)
East and southeast Asia	128.7 (126.9-130.5)	128.9 (127.5-130.4)	130.3 (128.4-132.2)
High-income Asia Pacific	133.1 (131.3-134.9)	129.0 (127.4-130.5)	125.0 (123.3-126.6)
High-income western	132.3 (131.1-133.6)	128.5 (127.5-129.5)	125.5 (124.2-126.9)
Latin America and Caribbean	134.5 (131.8-137.2)	131.7 (130.0-133.4)	130.7 (129.0-132.4)
Oceania	124.8 (120.9-128.7)	127.8 (123.6-132.0)	131.1 (126.2-136.3)
South Asia	128.9 (125.3-132.4)	130.8 (128.6-133.1)	132.3 (130.4-134.3)
Sub-Saharan Africa	135.5 (133.0-138.0)	136.6 (134.7-138.5)	136.8 (135.1-138.5)
**60-69 years**			
World	140.8 (139.5-142.1)	139.2 (138.3-140.0)	138.1 (137.1-139.1)
Central and eastern Europe	152.5 (150.2-155.0)	148.9 (146.4-151.4)	145.5 (142.7-148.4)
Central Asia, Middle East and north Africa	145.9 (142.7-149.0)	144.2 (142.3-146.2)	141.4 (139.5-143.3)
East and southeast Asia	135.9 (133.9-137.9)	136.3 (134.7-137.9)	137.8 (135.7-140.0)
High-income Asia Pacific	140.9 (138.9-142.8)	136.0 (134.3-137.7)	132.1 (130.0-134.1)
High-income western	141.0 (139.6-142.5)	137.5 (136.5-138.6)	133.8 (132.3-135.3)
Latin America and Caribbean	142.4 (139.3-145.4)	139.3 (137.3-141.2)	138.1 (136.2-140.1)
Oceania	130.5 (125.7-135.2)	134.0 (128.9-139.0)	137.7 (132.1-143.6)
South Asia	134.7 (130.6-138.6)	136.9 (134.3-139.4)	138.5 (136.3-140.7)
Sub-Saharan Africa	143.4 (140.6-146.3)	144.7 (142.5-146.9)	144.9 (142.9-146.8)
**70-79 years**			
World	145.8 (144.5-147.2)	144.2 (143.3-145.1)	143.0 (141.9-144.0)
Central and eastern Europe	158.1 (155.4-160.9)	154.2 (151.4-157.0)	150.9 (147.6-154.1)
Central Asia, Middle East and north Africa	150.5 (147.1-153.9)	148.7 (146.5-151.0)	145.8 (143.7-147.9)
East and southeast Asia	140.3 (138.3-142.5)	140.7 (138.9-142.4)	142.3 (140.1-144.5)
High-income Asia Pacific	145.6 (143.6-147.7)	140.6 (138.7-142.4)	136.4 (134.3-138.5)
High-income western	147.2 (145.7-148.7)	143.8 (142.6-144.9)	140.2 (138.6-141.8)
Latin America and Caribbean	147.5 (144.3-150.6)	144.3 (142.1-146.3)	143.0 (141.0-145.2)
Oceania	134.3 (128.3-140.2)	137.5 (131.4-143.7)	141.6 (135.0-148.4)
South Asia	138.0 (133.8-142.1)	140.2 (137.5-142.9)	142.0 (139.6-144.3)

Mean systolic blood pressure (mmHg)			
Super-region (Women)	Year		
	1985-1994	1995-2004	2005-2016
Sub-Saharan Africa	148.1 (145.1-151.2)	149.2 (146.9-151.7)	149.5 (147.3-151.7)

Mean diastolic blood pressure (mmHg)			
Super-region (Men)	Year		
	1985-1994	1995-2004	2005-2016
20-29 years			
World	74.5 (73.8-75.1)	74.7 (74.3-75.1)	74.8 (74.4-75.3)
Central and eastern Europe	79.0 (78.0-80.0)	78.2 (77.2-79.2)	77.8 (76.6-79.1)
Central Asia, Middle East and north Africa	74.4 (73.1-75.6)	74.5 (73.7-75.3)	74.7 (73.9-75.6)
East and southeast Asia	73.3 (72.5-74.2)	74.2 (73.5-74.9)	74.5 (73.6-75.4)
High-income Asia Pacific	74.0 (73.0-75.0)	73.4 (72.6-74.1)	73.1 (72.3-73.9)
High-income western	73.9 (73.3-74.5)	72.5 (72.0-73.1)	71.4 (70.7-72.0)
Latin America and Caribbean	75.4 (74.1-76.7)	75.2 (74.3-76.0)	74.3 (73.5-75.2)
Oceania	70.4 (68.3-72.4)	70.7 (68.4-73.0)	71.3 (68.7-73.9)
South Asia	75.8 (74.1-77.6)	76.1 (74.9-77.2)	77.0 (76.0-77.9)
Sub-Saharan Africa	72.9 (71.7-74.1)	73.3 (72.3-74.2)	73.5 (72.6-74.4)
30-39 years			
World	78.1 (77.5-78.7)	78.1 (77.7-78.5)	78.3 (77.8-78.7)
Central and eastern Europe	83.1 (82.1-84.1)	82.3 (81.3-83.4)	81.9 (80.7-83.1)
Central Asia, Middle East and north Africa	77.9 (76.6-79.1)	78.0 (77.2-78.8)	78.1 (77.3-78.9)
East and southeast Asia	76.4 (75.5-77.2)	77.4 (76.6-78.1)	77.6 (76.7-78.5)
High-income Asia Pacific	78.5 (77.5-79.4)	78.0 (77.2-78.8)	77.6 (76.7-78.4)
High-income western	78.5 (77.9-79.2)	77.3 (76.8-77.8)	76.2 (75.5-76.9)
Latin America and Caribbean	79.4 (78.0-80.7)	79.1 (78.2-80.0)	78.3 (77.4-79.1)
Oceania	73.3 (71.3-75.3)	73.6 (71.5-75.7)	74.1 (71.5-76.6)
South Asia	78.5 (76.7-80.2)	78.7 (77.5-79.8)	79.6 (78.6-80.6)
Sub-Saharan Africa	77.3 (76.2-78.5)	77.7 (76.8-78.7)	78.0 (77.1-78.9)
40-49 years			
World	80.9 (80.3-81.6)	80.9 (80.5-81.3)	80.9 (80.4-81.4)
Central and eastern Europe	86.4 (85.4-87.4)	85.6 (84.5-86.6)	85.1 (83.9-86.4)
Central Asia, Middle East and north Africa	80.7 (79.4-82.0)	80.9 (80.1-81.7)	81.0 (80.2-81.8)
East and southeast Asia	79.1 (78.3-80.0)	80.1 (79.4-80.9)	80.4 (79.4-81.4)
High-income Asia Pacific	81.8 (80.9-82.7)	81.2 (80.3-82.0)	80.7 (79.9-81.6)
High-income western	81.9 (81.2-82.5)	80.2 (79.7-80.8)	79.3 (78.5-80.0)
Latin America and Caribbean	82.4 (81.0-83.8)	82.1 (81.2-83.1)	81.2 (80.3-82.2)
Oceania	75.4 (73.3-77.5)	75.8 (73.7-78.0)	76.4 (73.9-78.8)
South Asia	80.4 (78.5-82.2)	80.6 (79.4-81.8)	81.6 (80.5-82.6)
Sub-Saharan Africa	80.7 (79.4-82.0)	81.1 (80.2-82.1)	81.3 (80.4-82.3)
50-59 years			
World	82.8 (82.2-83.5)	82.6 (82.2-83.1)	82.6 (82.1-83.1)
Central and eastern Europe	88.5 (87.4-89.6)	87.6 (86.5-88.7)	87.2 (85.8-88.5)
Central Asia, Middle East and north Africa	82.8 (81.4-84.2)	82.9 (82.0-83.7)	83.0 (82.1-83.8)
East and southeast Asia	81.1 (80.1-82.0)	82.1 (81.3-82.9)	82.4 (81.4-83.5)
High-income Asia Pacific	83.7 (82.7-84.6)	83.0 (82.1-83.8)	82.4 (81.5-83.3)
High-income western	83.6 (82.9-84.3)	81.8 (81.2-82.3)	80.3 (79.6-81.1)
Latin America and Caribbean	84.2 (82.7-85.7)	83.9 (82.9-84.9)	83.0 (82.0-84.0)

Mean diastolic blood pressure (mmHg)			
Super-region (Men)	Year		
	1985-1994	1995-2004	2005-2016
Oceania	76.5 (74.2-78.7)	76.9 (74.6-79.2)	77.6 (75.0-80.1)
South Asia	81.4 (79.5-83.4)	81.7 (80.4-82.9)	82.7 (81.6-83.8)
Sub-Saharan Africa	82.7 (81.4-84.0)	83.2 (82.1-84.2)	83.4 (82.4-84.4)
**60-69 years**			
World	82.5 (81.9-83.2)	82.5 (82.0-82.9)	82.2 (81.7-82.7)
Central and eastern Europe	88.5 (87.3-89.7)	87.6 (86.4-88.8)	87.0 (85.6-88.4)
Central Asia, Middle East and north Africa	83.0 (81.5-84.5)	83.2 (82.3-84.1)	83.1 (82.2-84.1)
East and southeast Asia	81.2 (80.2-82.2)	82.3 (81.5-83.2)	82.7 (81.5-83.8)
High-income Asia Pacific	83.1 (82.1-84.1)	82.2 (81.3-83.1)	82.0 (80.9-83.0)
High-income western	82.3 (81.6-83.0)	80.9 (80.3-81.5)	79.2 (78.4-80.0)
Latin America and Caribbean	83.8 (82.2-85.4)	83.4 (82.4-84.4)	82.5 (81.4-83.5)
Oceania	75.6 (73.0-78.3)	76.0 (73.3-78.6)	76.7 (73.9-79.6)
South Asia	80.9 (78.9-83.0)	81.2 (79.9-82.5)	82.3 (81.1-83.4)
Sub-Saharan Africa	82.4 (80.9-83.8)	82.9 (81.7-84.0)	83.2 (82.1-84.3)
**70-79 years**			
World	80.2 (79.5-80.9)	80.2 (79.7-80.7)	80.1 (79.6-80.7)
Central and eastern Europe	86.7 (85.3-88.2)	85.8 (84.3-87.3)	85.3 (83.6-87.0)
Central Asia, Middle East and north Africa	81.7 (80.0-83.4)	81.9 (80.8-83.0)	81.9 (80.8-83.0)
East and southeast Asia	79.5 (78.4-80.6)	80.8 (79.8-81.7)	81.1 (79.9-82.4)
High-income Asia Pacific	80.5 (79.3-81.6)	79.6 (78.6-80.6)	79.3 (78.1-80.5)
High-income western	78.8 (78.0-79.7)	77.5 (76.8-78.1)	76.1 (75.2-76.9)
Latin America and Caribbean	81.4 (79.7-83.2)	81.1 (79.9-82.2)	80.0 (78.9-81.2)
Oceania	73.1 (69.8-76.5)	73.4 (70.0-76.8)	74.2 (70.5-77.8)
South Asia	79.5 (77.2-81.7)	79.8 (78.3-81.2)	80.9 (79.6-82.2)
Sub-Saharan Africa	80.2 (78.4-81.8)	80.7 (79.4-82.1)	81.1 (79.8-82.4)

Mean diastolic blood pressure (mmHg)			
Super-region (Women)	Year		
	1985-1994	1995-2004	2005-2016
**20-29 years**			
World	71.7 (71.1-72.2)	71.8 (71.4-72.2)	72.1 (71.7-72.5)
Central and eastern Europe	75.0 (74.1-75.9)	73.6 (72.7-74.6)	72.9 (71.8-74.1)
Central Asia, Middle East and north Africa	72.4 (71.1-73.7)	72.5 (71.7-73.3)	72.1 (71.3-72.9)
East and southeast Asia	70.3 (69.5-71.1)	70.8 (70.1-71.5)	71.1 (70.3-71.9)
High-income Asia Pacific	68.4 (67.5-69.3)	67.2 (66.5-67.9)	66.5 (65.7-67.2)
High-income western	70.5 (69.9-71.1)	68.9 (68.4-69.4)	67.9 (67.3-68.6)
Latin America and Caribbean	72.5 (71.3-73.8)	71.9 (71.1-72.6)	70.8 (70.0-71.5)
Oceania	69.2 (67.3-71.0)	70.4 (68.3-72.5)	71.7 (69.4-74.2)
South Asia	73.5 (72.0-75.0)	74.0 (73.0-75.0)	74.8 (73.9-75.6)
Sub-Saharan Africa	71.9 (70.8-73.0)	72.8 (71.9-73.7)	73.5 (72.7-74.4)
**30-39 years**			
World	75.0 (74.4-75.6)	74.9 (74.5-75.3)	75.1 (74.7-75.5)
Central and eastern Europe	79.2 (78.3-80.2)	77.7 (76.7-78.8)	76.9 (75.7-78.1)
Central Asia, Middle East and north Africa	76.0 (74.7-77.4)	76.1 (75.3-76.9)	75.6 (74.8-76.4)
East and southeast Asia	73.4 (72.5-74.2)	73.9 (73.2-74.6)	74.3 (73.4-75.2)
High-income Asia Pacific	72.4 (71.5-73.3)	71.2 (70.4-72.0)	70.4 (69.5-71.2)

Mean diastolic blood pressure (mmHg)			
**Super-region (Women)**	**Year**		
	**1985-1994**	**1995-2004**	**2005-2016**
High-income western	74.2 (73.5-74.8)	72.5 (72.0-73.0)	71.5 (70.8-72.1)
Latin America and Caribbean	76.2 (74.9-77.6)	75.4 (74.6-76.3)	74.3 (73.4-75.1)
Oceania	70.9 (69.1-72.8)	72.2 (70.1-74.2)	73.6 (71.1-76.2)
South Asia	76.1 (74.4-77.8)	76.6 (75.5-77.8)	77.5 (76.5-78.4)
Sub-Saharan Africa	75.5 (74.3-76.7)	76.6 (75.6-77.5)	77.3 (76.4-78.1)
**40-49 years**			
World	78.8 (78.1-79.5)	78.6 (78.2-79.1)	78.6 (78.1-79.1)
Central and eastern Europe	84.2 (83.2-85.3)	82.6 (81.4-83.8)	81.7 (80.3-83.1)
Central Asia, Middle East and north Africa	80.2 (78.7-81.7)	80.3 (79.4-81.2)	79.7 (78.9-80.6)
East and southeast Asia	77.2 (76.2-78.1)	77.7 (76.9-78.4)	78.0 (77.0-79.1)
High-income Asia Pacific	77.0 (76.0-77.9)	75.6 (74.7-76.4)	74.6 (73.7-75.5)
High-income western	78.2 (77.4-78.9)	76.0 (75.4-76.6)	75.0 (74.3-75.8)
Latin America and Caribbean	80.2 (78.7-81.7)	79.3 (78.4-80.3)	78.0 (77.1-79.0)
Oceania	73.2 (71.0-75.3)	74.5 (72.3-76.8)	76.2 (73.5-78.9)
South Asia	79.0 (77.1-80.9)	79.6 (78.3-80.8)	80.5 (79.4-81.6)
Sub-Saharan Africa	79.5 (78.1-80.8)	80.7 (79.6-81.7)	81.4 (80.5-82.4)
**50-59 years**			
World	81.8 (81.1-82.5)	81.2 (80.7-81.7)	81.1 (80.6-81.7)
Central and eastern Europe	88.4 (87.1-89.6)	86.4 (85.1-87.6)	85.4 (83.9-86.9)
Central Asia, Middle East and north Africa	83.3 (81.6-85.0)	83.5 (82.5-84.5)	82.8 (81.8-83.8)
East and southeast Asia	79.9 (78.9-81.0)	80.6 (79.7-81.4)	80.9 (79.8-82.0)
High-income Asia Pacific	80.3 (79.2-81.4)	78.7 (77.8-79.6)	77.7 (76.7-78.7)
High-income western	81.1 (80.3-81.9)	78.5 (77.9-79.1)	77.1 (76.2-77.9)
Latin America and Caribbean	83.0 (81.3-84.6)	82.0 (81.0-83.0)	80.6 (79.5-81.6)
Oceania	74.5 (72.0-77.0)	75.9 (73.4-78.6)	77.8 (74.9-80.8)
South Asia	80.9 (78.8-83.0)	81.5 (80.1-82.9)	82.5 (81.4-83.7)
Sub-Saharan Africa	82.2 (80.6-83.7)	83.4 (82.3-84.6)	84.4 (83.3-85.4)
**60-69 years**			
World	82.6 (81.8-83.3)	81.9 (81.4-82.5)	81.4 (80.8-82.0)
Central and eastern Europe	90.0 (88.6-91.4)	87.9 (86.5-89.4)	86.7 (85.1-88.3)
Central Asia, Middle East and north Africa	84.2 (82.3-86.1)	84.4 (83.3-85.5)	83.7 (82.7-84.8)
East and southeast Asia	80.6 (79.4-81.8)	81.3 (80.3-82.2)	81.7 (80.4-82.9)
High-income Asia Pacific	81.1 (80.0-82.2)	79.3 (78.4-80.3)	78.4 (77.2-79.6)
High-income western	81.3 (80.4-82.1)	79.0 (78.3-79.7)	77.1 (76.2-77.9)
Latin America and Caribbean	83.4 (81.6-85.2)	82.3 (81.2-83.4)	80.8 (79.6-81.9)
Oceania	73.7 (70.8-76.6)	75.3 (72.3-78.2)	77.2 (74.0-80.6)
South Asia	81.0 (78.7-83.3)	81.7 (80.2-83.2)	82.8 (81.5-84.1)
Sub-Saharan Africa	82.5 (80.8-84.2)	83.9 (82.6-85.1)	84.9 (83.7-86.0)
**70-79 years**			
World	81.2 (80.4-82.0)	80.6 (80.0-81.1)	80.2 (79.6-80.8)
Central and eastern Europe	89.6 (87.9-91.3)	87.3 (85.6-88.9)	86.1 (84.2-87.9)
Central Asia, Middle East and north Africa	83.4 (81.3-85.4)	83.6 (82.3-84.9)	82.9 (81.7-84.2)
East and southeast Asia	79.6 (78.3-80.9)	80.3 (79.2-81.3)	80.7 (79.3-82.0)
High-income Asia Pacific	79.7 (78.5-80.9)	77.8 (76.7-78.8)	76.8 (75.5-78.1)
High-income western	79.3 (78.4-80.2)	77.1 (76.4-77.8)	75.5 (74.6-76.5)
Latin America and Caribbean	82.0 (80.0-83.9)	80.9 (79.6-82.1)	79.2 (77.9-80.4)
Oceania	71.2 (67.5-74.8)	72.8 (69.1-76.5)	75.0 (71.1-79.0)

Mean diastolic blood pressure (mmHg)			
**Super-region (Women)**	**Year**		
	**1985-1994**	**1995-2004**	**2005-2016**
South Asia	80.1 (77.6-82.6)	80.8 (79.3-82.5)	82.0 (80.6-83.5)
Sub-Saharan Africa	81.0 (79.2-82.9)	82.4 (80.9-83.8)	83.5 (82.2-84.9)

Supplementary Table 3. Number of studies and data points used in the analysis, by region.

Region	All post-1985 studies		Studies with complete metrics †		Studies with standard age groups ‡		Studies with sample size ≥25	
	No. studies	No. data points	No. studies	No. data points	No. studies	No. data points	No. studies	No. data points
Central and eastern Europe	138	1,370	124	1,264	106	801	106	757
Central Asia, Middle East and north Africa	141	1,610	115	1,358	107	983	107	940
East and southeast Asia	153	1,541	125	1,236	108	851	108	834
High-income Asia Pacific	113	1,235	103	1,143	85	896	78	773
High-income western	571	5,114	495	4,602	386	2,746	385	2,677
Latin America and the Caribbean	123	1,303	91	984	79	730	79	671
Oceania	42	432	35	395	35	276	34	246
South Asia	60	716	49	596	38	380	38	367
Sub-Saharan Africa	120	1,235	87	980	84	674	83	645
**Total**	**1,461**	**14,556**	**1,224**	**12,558**	**1,028**	**8,337**	**1,018**	**7,910**

† Including prevalence of raised blood pressure, mean systolic blood pressure and mean diastolic blood pressure.
‡ Ten-year age groups from 20-29 years to 70-79 years.

Supplementary Table 4. List of data sources used in the analysis and their characteristics.

	Country	Data years	Survey/Study name/Citation	Level of representative-ness	Rural, urban or both	Age range as in the NCD-RisC database		Sample size		Note
						Male	Female	Male	Female	
1	Albania	2008-2009	Demographic and Health Survey Albania 2008-2009	National	both	15-49	15-49	2,875	3,555	
2	Algeria	2003	STEPS	Community	both	25-64	25-64	1,612	2,450	
3	Algeria	2007-2009	The ISOR (InSulino-resistance in ORan) study	Community	urban	30-64	30-64	377	405	
4	American Samoa	1994	McGarvey ST. Cardiovascular disease (CVD) risk factors in Samoa and American Samoa, 1990-95. Pac Health Dialog 2001; 8: 157-62	National	both	25+	25+	168	242	
5	American Samoa	2004	STEPS	National	both	25-64	25-64	950	1,062	
6	Argentina	1985-1986	INTERSALT	Community	urban	20-59	20-59	100	100	
7	Argentina	2005	Encuensta Nacional de Nutricion y Salud 2005	National	both		10-49		5,469	
8	Argentina	2008-2011	The VELA Project	Community	rural	10+	10+	305	470	
9	Argentina	2011-2012	Detection and follow-up of cardiovascular disease and risk factors in the Southern Cone of Latin America. The CESCAS I Study	Community	urban	35-74	35-74	1,589	2,397	
10	Armenia	2005	Demographic and Health Survey Armenia 2005	National	both	15-49	15-49	1,208	6,188	
11	Armenia	2016	STEPS	National	both	18-69	18-69	601	1,459	
12	Australia	1988-1989	Dubbo Study of Australian Elderly	Community	urban	59+	59+	878	1,222	
13	Australia	1988-1989	MONICA, Perth inner	Community	urban	25-64	25-64	404	405	
14	Australia	1988-1989	MONICA, Perth outer	Community	urban	25-64	25-64	412	419	
15	Australia	1988-1989	MONICA, Newcastle	Subnational	urban	35-64	35-64	672	661	
16	Australia	1989	Risk Factor Prevalence Study	National	urban	20-69	20-69	4,551	4,727	
17	Australia	1990-1991	Canberra-Queanbeyan Longitudinal Study of the Elderly	Community	urban	70+	70+	454	380	
18	Australia	1992-1993	Australia Longitudinal Study of Ageing	Community	urban	65+	65+	842	771	
19	Australia	1994	MONICA, Perth outer	Community	urban	25-64	25-64	375	387	
20	Australia	1994	MONICA, Newcastle	Subnational	urban	35-64	35-64	637	688	
21	Australia	1994	MONICA, Perth inner	Community	urban	25-64	25-64	363	350	
22	Australia	1995-1996	National Nutrition Study	National	both	16+	16+	5,227	5,700	
23	Australia	1996-1998	Western Australian AAA Screening Program	Community	urban	65-84	65-84	12,202		
24	Australia	1999-2000	The Australian Diabetes, Obesity and Lifestyle Study 1999-2000	National	both	25+	25+	5,023	6,113	
25	Australia	1999-2003	North West Adelaide Health Study	Community	urban	18+	18+	1,932	2,123	
26	Australia	2004-2005	The Australian Diabetes, Obesity and Lifestyle Study 2004-2005	National	both	30+	30+	2,885	3,478	
27	Australia	2004-2006	North West Adelaide Health Study	Community	urban	20+	20+	1,515	1,669	
28	Australia	2008-2010	North West Adelaide Health Study	Community	urban	24+	24+	1,169	1,317	
29	Australia	2011-2012	Australian Health Survey	National	both	10+	10+	11,290	12,309	
30	Australia	2012	The Australian Diabetes, Obesity and Lifestyle Study 2012	National	both	37+	37+	2,052	2,536	
31	Australia	2014-2015	National Health Survey	National	both	18+	18+	5,158	5,865	
32	Austria	1992	Vorarlberg Health Monitoring and Promotion Programme	Subnational	both	18+	18+	14,161	18,825	
33	Austria	1998	Vorarlberg Health Monitoring and Promotion Programme	Subnational	both	18+	18+	16,154	20,917	
34	Austria	2004	Vorarlberg Health Monitoring and Promotion Programme	Subnational	both	18+	18+	20,157	23,891	
35	Azerbaijan	2006	Demographic and Health Survey Azerbaijan 2006	National	both	15-59	15-49	2,551	8,114	
36	Bangladesh	2011	Demographic and Health Survey Bangladesh 2011	National	both	16+	12+	3,905	3,951	
37	Bangladesh	2015	An assessment of BRAC Health Nutrition and Population Programme and benchmark survey of Sustainable Development Goal – 2015	National	rural	35+	35+	5,234	6,568	
38	Barbados	2011-2013	Health of the Nation (HotN)	National	both	25+	25+	470	739	
39	Belarus	2016	STEPS	National	both	18-69	18-69	2,086	2,896	
40	Belgium	1984-1985	Belgian Interuniversity Research on Nutrition and Health	National	both	25-74	25-74	5,899	5,287	
41	Belgium	1985-1986	INTERSALT, Ghent	Community	urban	20-59	20-59	100	100	
42	Belgium	1985-1987	INTERSALT, Charleroi	Community	urban	20-59	20-59	82	75	
43	Belgium	1985-1987	MONICA, Charleroi	Community	urban	25-64	25-64	346	326	
44	Belgium	1985-1987	MONICA, Ghent	Community	urban	25-64	25-64	550	459	
45	Belgium	1985-1990	Flemish Study on Environment, Genes and Health Outcomes	Community	rural	20-90	20-90	659	696	
46	Belgium	1987-1990	MONICA, Charleroi	Community	urban	25-64	25-64	325	301	
47	Belgium	1988-1990	MONICA, Ghent	Community	urban	25-64	25-64	456	449	
48	Belgium	1990-1992	MONICA, Ghent	Community	urban	25-64	25-64	509	475	

	Country	Data years	Survey/Study name/Citation	Level of representative-ness	Rural, urban or both	Age range as in the NCD-RisC database		Sample size		Note
						Male	Female	Male	Female	
49	Belgium	1990-1993	MONICA, Charleroi	Community	urban	25-64	25-64	337	332	
50	Belgium	1991-1994	Flemish Study on Environment, Genes and Health Outcomes	Community	rural	26-88	26-88	394	416	
51	Belgium	1992-1995	Flemish Study on Environment, Genes and Health Outcomes	Community	rural	27-89	27-89	298	312	
52	Belgium	1996-1998	Flemish Study on Environment, Genes and Health Outcomes	Community	rural	10-84	10-84	404	402	
53	Belgium	1998	Flemish Study on Environment, Genes and Health Outcomes	Community	rural	32-86	32-86	320	359	
54	Belgium	1998-2000	Flemish Study on Environment, Genes and Health Outcomes	Community	rural	10-80	10-80	222	219	
55	Belgium	1999-2001	Flemish Study on Environment, Genes and Health Outcomes	Community	rural	10-81	10-81	234	255	
56	Belgium	2001	Flemish Study on Environment, Genes and Health Outcomes	Community	rural	10-78	10-78	243	222	
57	Belgium	2002-2003	Flemish Study on Environment, Genes and Health Outcomes	Community	rural	10-81	10-81	180	200	
58	Belgium	2003	The European Male Ageing Study	Community	both	40+		448		
59	Belgium	2002-2005	Flemish Study on Environment, Genes and Health Outcomes	Community	rural	10-88	10-88	447	462	
60	Belgium	2005-2008	Flemish Study on Environment, Genes and Health Outcomes	Community	rural	10-89	10-89	464	473	
61	Belgium	2008	The European Male Ageing Study	Community	both	40+		385		
62	Belgium	2009-2013	Flemish Study on Environment, Genes and Health Outcomes	Community	rural	20-88	20-88	330	335	
63	Belgium	2010-2015	Flemish Study on Environment, Genes and Health Outcomes	Community	rural	15-87	15-87	391	411	
64	Belize	2004-2005	CAMDI	National	both	20+	20+	999	1,434	
65	Benin	2007	STEPS	Community	urban	25-64	25-64	954	1,468	
66	Benin	2008	STEPS	National	both	25-64	25-64	3,445	3,383	
67	Benin	2011-2012	Demographic and Health Survey Benin 2011-2012	National	both	30-64	15-49	2,681	4,763	
68	Benin	2015	STEPS	National	both	18-69	18-69	2,310	2,545	
69	Bhutan	2007	STEPS	Community	urban	25-74	25-74	1,131	1,328	
70	Bhutan	2014	STEPS	National	both	18-69	18-69	1,071	1,680	
71	Bosnia and Herzegovina	2002	Non-communicable disease risk factor survey, Federation of B&H	Subnational	both	25-64	25-64	1,118	1,621	
72	Bosnia and Herzegovina	2012	Non-communicable disease risk factor survey, Federation of B&H	Subnational	rural	18+	18+	1,177	1,287	
73	Bosnia and Herzegovina	2012	Non-communicable disease risk factor survey, Federation of B&H	Subnational	urban	18+	18+	589	706	
74	Botswana	2007	STEPS	National	both	25-64	25-64	1,266	2,600	
75	Botswana	2014	STEPS	National	both	15-69	15-69	1,299	2,618	
76	Brazil	1995-1996	Cohort study from Porto Alegre	Community	urban	18+	18+	490	596	
77	Brazil	1996-1997	The Bambui Cohort Study of Ageing	Community	urban	18+	18+	947	1,367	
78	Brazil	1999-2000	Pelotas cross-sectional survey	Community	urban	20-69	20-69	844	1,116	
79	Brazil	2003-2005	São Paulo Ageing & Health Study	Community	urban	65+	65+	781	1,200	
80	Brazil	2004-2006	Hearts of Brazil	National	urban	18+	18+	574	654	
81	Brazil	2006-2007	SOFT study	Community	urban	18+	18+	739	1,110	
82	Brazil	2009-2010	EpiFloripa Cohort Study of Ageing - Wave 1	Community	urban	60+	60+	610	1,069	
83	Brazil	2010-2013	Baependi Heart Study	Community	urban	18+	18+	609	885	
84	Brazil	2012-2013	Prevalence of Leptin Polymorphism Gln223Arg	Community	urban	18+	18+	282	526	
85	Brazil	2013	Pesquisas Nacional de Saude	National	both	35+	35+	15,948	21,014	
86	Brazil	2013-2014	EpiFloripa Cohort Study of Ageing - Wave 2	Community	urban	63+	63+	414	766	
87	Brunei Darussalam	2010-2011	National Health And Nutritional Status Survey (NHANSS)	National	both	10-75	10-75	898	1,018	
88	Brunei Darussalam	2015-2016	National Non-Communicable Diseases Survey (NNCDS)	National	both	18-69	18-69	903	1,159	
89	Burkina Faso	2013	STEPS	National	both	25-64	25-64	2,231	2,255	
90	Cabo Verde	2007	STEPS	National	both	25-64	25-64	663	1,076	
91	Cambodia	2010	STEPS	National	both	25-64	25-64	1,884	3,346	
92	Cameroon	1998-1999	ENHIP	Community	rural	15+	15+	503	722	
93	Cameroon	1998-1999	ENHIP	Community	urban	15+	15+	509	626	
94	Cameroon	2003	STEPS	Subnational	urban	15+	15+	3,723	5,550	
95	Cameroon	2007	Cameroon Burden of Diabetes - Second Survey	Subnational	urban	18+	18+	3,118	4,108	
96	Canada	1985-1986	INTERSALT, StJohns	Community	urban	20-59	20-59	100	100	
97	Canada	1985-1988	MONICA, Halifax	Community	both	25-64	25-64	438	421	
98	Canada	1986-1992	Canada Heart Health Survey	National	both	18-74	18-74	11,353	11,737	
99	Canada	1995	MONICA, Halifax	Community	both	25-64	25-64	274	287	

	Country	Data years	Survey/Study name/Citation	Level of representative-ness	Rural, urban or both	Age range as in the NCD-RisC database		Sample size		Note
						Male	Female	Male	Female	
100	Canada	2007-2009	Canadian Health Measures Survey, Cycle 1	National	both	6-79	6-79	3,214	3,392	
101	Canada	2009-2011	Canadian Health Measures Survey, Cycle 2	National	both	6-79	6-79	2,764	3,019	
102	Canada	2012-2013	Canadian Health Measures Survey, Cycle 3	National	both	6-79	6-79	2,594	2,619	
103	Canada	2014-2015	Canadian Health Measures Survey, Cycle 4	National	both	6-79	6-79	2,603	2,602	
104	Central African Republi	2010	STEPS	Community	both	25-64	25-64	1,863	1,992	
105	Chad	2008	STEPS	Community	urban	25-64	25-64	982	822	
106	Chile	1992-1993	Miquel JF, C. Covarrubias, L. Villarroel, G. Mingrone, A.V. Greco, P. Carvallo, G. Marshall, G. Del Pino, y F. Nervi. Genetic epidemiology of cholesterol cholelithiasis among Chilean Hispanics and Maoris. Gastroenterology 1998; 115: 937-946	Community	urban	18+	18+	660	1,032	
107	Chile	2000	Nervi F, Miquel J.F., Alvarez M, Ferreccio C, García-Zattera MJ, González R, Pérez-Ayuso RM, Rigotti A, Villarroel L. Gallbladder disease is associated with insulin resistance in a high risk population. J Hepatol 2006; 45: 299 - 305	Community	urban	18+	18+	335	625	
108	Chile	2003	Encuesta Nacional de Salud	National	both	17+	17+	1,563	1,866	
109	Chile	2009-2010	Encuesta Nacional de Salud	National	both	15+	15+	1,994	2,972	
110	Chile	2011-2012	Detection and follow-up of cardiovascular disease and risk factors in the Southern Cone of Latin America. The CESCAS I Study	Community	urban	35-74	35-74	923	1,027	
111	China	1984-1985	Sino-MONICA Beijing	Subnational	both	25-64	25-64	816	857	
112	China	1986	INTERSALT, Tianjin	Community	urban	20-59	20-59	100	100	
113	China	1986	INTERSALT, Beijing	Community	urban	20-59	20-59	100	100	
114	China	1986	INTERSALT, Nanning	Community	both	20-59	20-59	100	100	
115	China	1986-1989	Sino-MONICA Shanghai	Subnational	rural	25-64	25-64	675	753	
116	China	1988	Sino-MONICA Hebei	Subnational	both	25-64		610		
117	China	1988	Sino-MONICA Heilongjiang	Subnational	urban	25-64	25-64	800	800	
118	China	1988	Sino-MONICA Henan	Subnational	urban	25-64	25-64	345	427	
119	China	1988	Sino-MONICA Neimenggu	Subnational	urban	25-64	25-64	397	400	
120	China	1988	Sino-MONICA Sichuan	Subnational	both	25-64	25-64	313	334	
121	China	1988	Sino-MONICA Shandong	Subnational	urban	25-64	25-64	211	225	
122	China	1988-1989	Sino-MONICA Beijing	Subnational	both	25-64	25-64	703	863	
123	China	1988-1989	Sino-MONICA Jilin	Subnational	urban	25-64	25-64	394	408	
124	China	1988-1989	Sino-MONICA Jiangxi	Subnational	urban	25-64	25-64	379	386	
125	China	1988-1989	Sino-MONICA Liaoning	Subnational	both	25-64	25-64	728	734	
126	China	1988-1990	East Beijing Study 2	Community	urban	20-84	20-84	139	150	
127	China	1989	Sino-MONICA Fujian	Subnational	urban	25-64	25-64	179	191	
128	China	1989	China Health and Nutrition Study	National	both	10-45	10-45	2,383	2,570	1
129	China	1989	Sino-MONICA Jiangsu	Subnational	rural	25-64	25-64	398	399	
130	China	1990-1991	China Prospective Study	National	both	40-79		221,080		
131	China	1991	China National Hypertension Survey Epidemiology Follow-up Study	National	both	40+	40+	83,347	86,183	
132	China	1991	Sino-MONICA Shanghai	Subnational	rural	30-64	30-64	564	624	
133	China	1991	China Health and Nutrition Study	National	both	10+	10+	5,024	5,412	1
134	China	1991-1992	Fangshan Cohort Study	Community	urban	34-86	34-86	879	1,746	
135	China	1992	Sino-MONICA Sichuan	Subnational	both	25-64	25-64	628	536	
136	China	1992	Huashan Study	Community	urban	35-75	35-75	896	970	
137	China	1992-1993	Anzhen 02 Cohort Study	Community	urban	34-65	34-65	2,032	2,120	
138	China	1993	China Health and Nutrition Study	National	both	10+	10+	4,759	5,073	1
139	China	1993	Sino-MONICA Beijing	Subnational	both	25-64	25-64	613	816	
140	China	1993	Sino-MONICA Jiangsu	Subnational	urban	25-64	25-64	862	365	
141	China	1993	Sino-MONICA Liaoning	Subnational	both	25-64	25-64	493	500	
142	China	1993	Sino-MONICA Anhui	Subnational	urban	25-64	25-64	193	195	
143	China	1997	China Health and Nutrition Study	National	both	10+	10+	5,045	5,217	1
144	China	1997	INTERMAP, Guangxi	Community	rural	40-59	40-59	140	138	
145	China	1997	INTERMAP, Shanxi	Community	rural	40-59	40-59	143	146	

	Country	Data years	Survey/Study name/Citation	Level of representative-ness	Rural, urban or both	Age range as in the NCD-RisC database		Sample size		Note
						Male	Female	Male	Female	
146	China	1997	INTERMAP, Beijing	Community	rural	40-59	40-59	133	139	
147	China	2000	China Health and Nutrition Study	National	both	10+	10+	5,497	5,785	1
148	China	2000-2001	The International Collaborative Study of Cardiovascular Disease in ASIA	National	both	35-74	35-74	7,515	8,008	
149	China	2002	China National Nutrition and Health Survey	National	both	20+	20+	63,471	76,623	
150	China	2004	China Health and Nutrition Study	National	both	10+	10+	4,978	5,307	1
151	China	2004-2008	China Kadoorie Biobank baseline survey	Subnational	rural	35-74	35-74	115,791	162,848	
152	China	2004-2008	China Kadoorie Biobank baseline survey	Subnational	urban	35-74	35-74	89,218	132,859	
153	China	2006	Beijing Eye Study	Community	both	45+	45+	1,396	1,826	
154	China	2006	China Health and Nutrition Study	National	both	10+	10+	4,750	5,238	1
155	China	2008	China Health and Retirement Longitudinal Study (CHARLS), pilot survey	Subnational	both	45+	45+	934	960	
156	China	2008-2009	Chinese Longitudinal Healthy Longevity Survey	National	both	65+	65+	6,743	8,965	
157	China	2007-2010	SAGE	National	both	50+	50+	5,808	6,689	
158	China	2008-2010	Fangshan Family-based Ischemic Stroke Study in China (FISSIC) program	Community	rural	40+	40+	20,241	37,772	
159	China	2009	China Health and Nutrition Study	National	both	10+	10+	4,940	5,291	1
160	China	2010	China Noncommunicable Disease Surveillance	National	both	18+	18+	45,077	53,460	
161	China	2011	Beijing Eye Study	Community	both	50+	50+	1,499	1,949	
162	China	2011	China Health and Nutrition Study	National	both	10+	10+	6,377	7,116	1
163	China	2011-2012	China Health and Retirement Longitudinal Study (CHARLS), baseline survey	National	both	45+	45+	6,416	7,077	
164	China	2012	China Health and Retirement Longitudinal Study (CHARLS), wave 2 pilot survey	Subnational	both	45+	45+	873	957	
165	China	2012-2013	The Kailuan Study	Community	urban	18+	18+	81,496	21,590	
166	China	2013	China Health and Retirement Longitudinal Study (CHARLS), wave 2 survey	National	both	45+	45+	6,031	6,711	
167	China	2012-2015	Shanghai Men's Health Study	Community	urban	47-87		33,177		
168	China	2012-2015	Shanghai Women's Health Study	Community	urban		52-88		42,981	
169	China (Hong Kong SAI	1985-1986	Shatin New Town Study	Community	urban	70+	70+	285	697	
170	China (Hong Kong SAI	1991	The Hong Kong study on Health, health risk and quality of life in the Chinese elderly cohort	Community	both	70+	70+	992	1,027	
171	China (Hong Kong SAI	1995-1996	Hong Kong Cardiovascular Risk Factor Prevalence Study 1995-1996	National	both	25-74	25-74	1,410	1,482	
172	Colombia	1986	INTERSALT	Community	rural	20-59	20-59	96	95	
173	Colombia	2007	Encuesta Nacional de Salud	National	both	18-69	18-69	5,510	7,744	
174	Colombia	2010	STEPS	Subnational	both	15-64	15-64	984	1,302	
175	Colombia	2015	STEPS	Subnational	both	15-64	15-64	978	1,180	
176	Comoros	2011	STEPS	National	both	25-64	25-64	1,568	3,591	
177	Congo	2004	STEPS	Community	urban	25-64	25-64	1,014	914	
178	Cook Islands	2003	STEPS	National	both	25-64	25-64	925	959	
179	Cook Islands	2014	STEPS	National	both	18-64	18-64	460	475	
180	Costa Rica	2004	CAMDI	Community	urban	20+	20+	522	895	
181	Costa Rica	2004-2006	Costa Rican Longevity and Healthy Aging Study Pre-1945 Cohort Wave 1	National	both	60+	60+	1,278	1,515	
182	Costa Rica	2006-2008	Costa Rican Longevity and Healthy Aging Study Pre-1945 Cohort Wave 2	National	both	62+	62+	1,060	1,261	
183	Costa Rica	2009-2010	Costa Rican Longevity and Healthy Aging Study Pre-1945 Cohort Wave 3	National	both	64+	64+	808	1,008	
184	Costa Rica	2010	Costa Rican National Cardiovascular Risk Factors Survey, 2010	National	both	20+	20+	1,016	2,618	
185	Cote d'Ivoire	2005	STEPS	National	urban	15-64	15-64	1,074	1,440	
186	Cote d'Ivoire	2005	STEPS	National	rural	15-64	15-64	890	1,019	
187	Croatia	2003	2003 CAHS - Croatian Health Survey project of the Ministry of Health and Social Welfare	National	both	18+	18+	2,878	6,162	
188	Croatia	2005	Endemic Nephropathy and Arterial hypertension (ENAH)	Subnational	rural	18+	18+	267	401	
189	Croatia	2008	Endemic Nephropathy and Arterial hypertension (ENAH)	Subnational	rural	18+	18+	263	465	
190	Croatia	2010	Endemic Nephropathy and Arterial hypertension (ENAH)	Subnational	rural	18+	18+	258	406	
191	Croatia	2015	Endemic Nephropathy and Arterial hypertension (ENAH) Follow-up Study	Subnational	rural	18+	18+	225	462	
192	Cuba	1999-2000	The Survey on Health, Well-Being, and Aging in Latin America and the Caribbean	Community	urban	60+	60+	645	1,087	2
193	Cuba	2010	National Risk Factor Survey	National	both	15+	15+	3,350	3,884	
194	Cuba	2011	Non communicable disease risk factor in Cienfuegos	Community	urban	15-74	15-74	616	880	
195	Czech Republic	1985	MONICA, Czech Republic	National	both	25-64	25-64	1,245	1,309	
196	Czech Republic	1988	MONICA, Czech Republic	National	both	25-64	25-64	1,357	1,411	

	Country	Data years	Survey/Study name/Citation	Level of representative-ness	Rural, urban or both	Age range as in the NCD-RisC database		Sample size		Note
						Male	Female	Male	Female	
197	Czech Republic	1992	MONICA, Czech Republic	National	both	25-64	25-64	1,134	1,209	
198	Czech Republic	1997-1998	Czech post-MONICA	National	both	25-64	25-64	1,529	1,666	
199	Czech Republic	2000-2001	Czech post-MONICA	National	both	25-64	25-64	1,631	1,690	
200	Czech Republic	2002-2005	Health, Alcohol and Psychosocial factors In Eastern Europe	Subnational	urban	45-69	45-69	3,240	3,887	
201	Czech Republic	2007-2008	Czech post-MONICA	National	both	25-64	25-64	1,718	1,861	
202	Denmark	1985	INTERSALT	Community	urban	20-59	20-59	99	100	
203	Denmark	1991-1992	MONICA, Glostrup	Community	urban	29-61	29-61	808	816	
204	Denmark	1992-1994	Obesity Research Group-Copenhagen City Heart Study 3	Subnational	both	33-73		922		
205	Denmark	1993-1997	EPIC Aarhus	Community	urban	50-65	50-65	8,424	8,712	
206	Denmark	1993-1997	EPIC Copenhagen	Community	urban	50-65	50-65	18,717	21,114	
207	Denmark	2006-2008	The Health2006 Cohort	Community	urban	18-71	18-71	1,553	1,918	
208	Denmark	2007-2008	The Danish Health Examination Survey 2007-2008	National	both	18+	18+	7,358	10,655	
209	Denmark	2012-2015	Danish study of Functional Disorders (DanFunD)	Subnational	urban	18-72	18-72	3,455	4,036	
210	Dominica	2007	STEPS	National	both	15-64	15-64	459	557	
211	DR Congo	2005	STEPS	Community	urban	15+	15+	761	1,152	
212	Ecuador	2011-2013	Encuesta Nacional de Salud y Nutricion	National	both	10-59	10-59	18,673	24,035	
213	Egypt	1991-1993	The Egyptian National Hypertension Study	National	both	25+	25+	3,253	4,201	
214	Egypt	2002	National Survey of Smoking, Obesity, Blood Pressure and Blood Glucose	National	both	10+	10+	2,808	3,444	
215	Egypt	2003-2004	Metabolic and cardiovascular risk profiles and hepatitis C virus infection in rural Egypt	Community	rural	25+	25+	316	446	
216	Egypt	2005	STEPS	National	both	15-65	15-65	4,743	4,418	
217	Egypt	2007-2009	Hepatitis C infection and clearance: impact on atherosclerosis and cardiometabolic risk factors	Community	rural	35+	35+	638	840	
218	Egypt	2008	Demographic and Health Survey Egypt 2008	National	both	10-59	10-59	5,424	6,156	
219	Egypt	2011	STEPS	National	both	15-65	15-65	1,775	3,009	
220	Egypt	2015	Demographic and Health Survey Egypt 2015	National	both	15-59	15-59	7,429	8,639	
221	El Salvador	2004	CAMDI	Community	urban	20+	20+	397	811	
222	Eritrea	2004	STEPS	National	both	15-64	15-64	1,145	1,109	
223	Eritrea	2010	STEPS	National	both	25-74	25-74	1,713	4,290	
224	Estonia		Abina et al., Blood Press 2003; 12: 111-21	Community	urban	20-54	20-54			
225	Estonia		Abina et al., Blood Press 2003; 12: 111-21	Community	urban	20-54	20-54			
226	Estonia		Abina et al., Blood Press 2003; 12: 111-21	Community	urban	20-54	20-54			
227	Estonia	2002	Estonian Biobank	National	both	18+	18+	89	216	
228	Estonia	2003	The European Male Ageing Study	Community	both	40+		419		
229	Estonia	2003	Estonian Biobank	National	both	18+	18+	2,685	5,668	
230	Estonia	2004	Estonian Biobank	National	both	18+	18+	527	944	
231	Estonia	2007	Estonian Biobank	National	both	18+	18+	1,000	2,185	
232	Estonia	2008	The European Male Ageing Study	Community	both	40+		306		
233	Estonia	2008	Estonian Biobank	National	both	18+	18+	5,148	10,967	
234	Estonia	2009	Estonian Biobank	National	both	18+	18+	3,963	6,489	
235	Estonia	2010	Estonian Biobank	National	both	18+	18+	4,054	7,042	
236	Estonia	2011	Estonian Biobank	National	both	18+	18+	111	174	
237	Estonia	2012	Estonian Biobank	National	both	18+	18+	89	130	
238	Estonia	2013	Estonian Biobank	National	both	18+	18+	110	146	
239	Ethiopia	2006	Surveillance of NCD Risk Factors in Addis Ababa (STEPS_Addis2006)	Subnational	urban	25-64	25-64	1,646	2,317	
240	Fiji	2002	STEPS	National	both	15-64	15-64	2,686	3,819	
241	Fiji	2011	STEPS	National	both	25-64	25-64	1,118	1,413	
242	Finland	1985	INTERSALT, Turku	Community	urban	20-59	20-59	100	100	
243	Finland	1985-1986	INTERSALT, Joensuu	Community	urban	20-59	20-59	100	100	
244	Finland	1987	MONICA, North Karelia/Kuopio/Turku/Loimaa	Subnational	both	25-64	25-64	2,898	3,152	
245	Finland	1989	Finnish cohort of the FINE study	Community	rural	70-89		462		
246	Finland	1992	The National FINRISK Study	Subnational	both	25-64	25-64	2,847	3,201	
247	Finland	1996-1998	Savitaipale Study, Baseline	Community	rural	40-66	40-66	573	573	

	Country	Data years	Survey/Study name/Citation	Level of representative-ness	Rural, urban or both	Age range as in the NCD-RisC database		Sample size		Note
						Male	Female	Male	Female	
248	Finland	1997	The National FINRISK Study	National	both	25-74	25-74	4,248	4,184	
249	Finland	2000-2001	Health 2000 Survey	National	both	30+	30+	2,870	3,463	
250	Finland	2002	The National FINRISK Study	National	both	25-74	25-74	3,267	3,814	
251	Finland	2001-2004	Helsinki Birth Cohort Study	Community	urban	56-69	56-69	928	1,075	
252	Finland	2007	The National FINRISK Study	National	both	25-74	25-74	2,926	3,310	
253	Finland	2007-2008	Savitaipale Study, Follow-up	Community	rural	51-75	51-75	433	484	
254	Finland	2008	Control group for Finnish male former elite athletes	National	both	61+		207		
255	Finland	2011-2012	Health 2011 Survey	National	both	30+	30+	2,061	2,563	
256	Finland	2012	The National FINRISK Study	National	both	25-74	25-74	2,771	3,042	
257	France	1985-1987	MONICA, Strasbourg	Subnational	both	35-64	35-64	667	714	
258	France	1985-1987	MONICA, Toulouse	Subnational	both	35-64	35-64	678	645	
259	France	1986-1989	MONICA, Lille	Community	urban	25-64	25-64	882	737	
260	France	1988-1991	MONICA, Toulouse	Subnational	both	35-64	35-64	586		
261	France	1994-1996	MONICA, Toulouse	Subnational	both	35-64	35-64	609	566	
262	France	1995-1997	MONICA, Lille	Community	urban	36-67	36-66	598	594	
263	France	1995-1997	MONICA, Strasbourg	Subnational	both	35-64	35-64	527	533	
264	France	1999-2001	The Three city Study	Community	urban	65+	65+	3,511	5,412	
265	France	2001-2003	The Three city Study	Community	urban	66+	66+	3,087	4,869	
266	France	2003-2005	The Three city Study	Community	urban	68+	68+	2,692	4,371	
267	France	2004-2006	National Monitoring of Arterial Risk in Lille (MONA LISA Lille)	Community	urban	35-75	35-75	797	796	
268	France	2005-2007	National Monitoring of Arterial Risk in Bas-Rhin (MONA LISA Bas-Rhin)	Subnational	both	35-74	35-74	781	785	
269	France	2006-2007	Etude Nationale Nutrition Santé	National	both	18-74	18-74	792	1,330	
270	France	2011-2013	Enquête LIttorale Souffle Air Biologie EnvironnemenT (ELISABET) Lille	Community	urban	40-64	40-64	753	850	
271	France	2011-2013	Enquête LIttorale Souffle Air Biologie EnvironnemenT (ELISABET) Dunkerque	Community	urban	40-64	40-64	749	798	
272	French Polynesia	2010	STEPS	National	both	18-64	18-64	993	1,308	
273	Gabon	2009	STEPS	Community	urban	15-64	15-64	1,061	1,539	
274	Gambia	2010	STEPS	National	both	25-64	25-64	1,643	1,953	
275	Georgia	2010	STEPS	National	both	18-64	18-64	1,849	4,476	
276	Georgia	2016	STEPS	National	both	18-69	18-69	1,193	2,789	
277	Germany	1984-1985	MONICA, Augsburg	Community	both	25-64	25-64	2,017	1,991	
278	Germany	1984-1986	MONICA, Cottbus County	Community	urban	25-64	25-64	657	739	
279	Germany	1985-1986	INTERSALT, Heidelberg	Community	urban	20-59	20-59	97	99	
280	Germany	1985-1986	INTERSALT, Cottbus	Community	urban	20-59	20-59	99	99	
281	Germany	1985-1986	INTERSALT, Bernried	Community	urban	20-59	20-59	99	98	
282	Germany	1987-1988	MONICA, Erfurt	Community	urban	25-64	25-64	879	915	
283	Germany	1988	MONICA, Berlin-Lichtenberg	Community	urban	25-64	25-64	689	727	
284	Germany	1988	MONICA, Bremen North/West	Community	urban	25-69	25-69	621	632	
285	Germany	1988	German Cardiovascular Prevention Study (GCP) - National Health Survey 1988	Subnational	both	25-69	25-69	2,647	2,684	
286	Germany	1988	MONICA, Chemnitz	Community	urban	25-64	25-64	288	382	
287	Germany	1988	MONICA, Zwickau	Community	urban	25-64	25-64	193	250	
288	Germany	1988	MONICA, Bremen Center/South/East	Community	urban	25-69	25-69	502	582	
289	Germany	1988-1989	MONICA, Rest of Karl-Marx-Stadt County	Subnational	urban	25-64	25-64	543	626	
290	Germany	1988-1989	MONICA, Halle County	Subnational	urban	25-64	25-64	963	1,202	
291	Germany	1989-1990	MONICA, Cottbus County	Community	urban	25-64	25-64	542	529	
292	Germany	1989-1990	MONICA, Augsburg	Community	both	25-64	25-64	1,952	1,997	
293	Germany	1991-1992	MONICA, Bremen Center/South/East	Community	urban	25-69	25-69	524	547	
294	Germany	1991-1992	MONICA, Bremen North/West	Community	urban	25-69	25-69	598	672	
295	Germany	1991-1992	German Cardiovascular Prevention Study (GCP) - National Health Survey 1991	Subnational	both	25-69	25-69	2,622	2,686	
296	Germany	1991-1992	First National Examination of life conditions, Environment and Health in East Germany 1991/92	Subnational	both	25-69	25-69	1,051	1,160	
297	Germany	1991-1992	MONICA, Erfurt	Community	urban	25-64	25-64	588	574	
298	Germany	1993-1994	MONICA, Zwickau	Community	urban	25-64	25-64	139	186	

	Country	Data years	Survey/Study name/Citation	Level of representative-ness	Rural, urban or both	Age range as in the NCD-RisC database		Sample size		Note
						Male	Female	Male	Female	
299	Germany	1993-1994	MONICA, Chemnitz	Community	urban	25-64	25-64	407	426	
300	Germany	1994-1995	MONICA, Augsburg	Community	both	25-64	25-64	1,905	1,994	
301	Germany	1994-1998	EPIC Potsdam	Community	urban	40-64	35-64	9,541	15,239	
302	Germany	1994-1998	EPIC Heidelberg	Community	urban	40-64	35-64	4,396	5,369	
303	Germany	1997-1999	German National Health Interview and Examination Survey (GNHIES98)	National	both	18-79	18-79	3,445	3,616	
304	Germany	1997-2001	Study of Health in Pomerania (SHIP-0) baseline study	Subnational	both	20-80	20-80	2,112	2,187	
305	Germany	1999-2001	KORA S4 Study: Kooperative Research in the Region of Augsburg Survey 4	Community	both	24-75	24-75	2,082	2,161	
306	Germany	2000-2002	Epidemiological study of the chances of prevention, early recognition and optimal treatment of chronic diseases in an elderly population (ESTHER)	Subnational	both	50-75	50-75	4,315	5,306	
307	Germany	2002-2006	Study of Health in Pomerania (SHIP-1) 5-year follow-up	Subnational	both	25-85	25-85	1,584	1,707	
308	Germany	2006-2008	KORA F4 Study: Kooperative Research in the Region of Augsburg Follow-Up of Survey 4	Community	both	31-82	31-82	1,482	1,591	
309	Germany	2008-2011	Epidemiological study of the chances of prevention, early recognition and optimal treatment of chronic diseases in an elderly population (ESTHER)	Subnational	both	58-84	58-84	1,297	1,443	
310	Germany	2008-2011	German Health Interview and Examination Survey for adults 2008-11 (DEGS1)	National	both	18-79	18-79	3,405	3,665	
311	Germany	2008-2012	Study of Health in Pomerania, second cohort (SHIP-TREND)	Subnational	both	20-79	20-79	2,095	2,228	
312	Ghana	2006	STEPS	Community	urban	25+	25+	887	1,698	
313	Ghana	2007-2008	SAGE	National	both	50+	50+	2,215	2,034	
314	Ghana	2012	The Burden and Correlates of Hypertension in Rural Ghana: A Cross-Sectional Study	Subnational	rural	35+	35+	150	271	
315	Ghana	2012-2014	Research on Obesity and Diabetes among African Migrants (RODAM), control group	Subnational	urban	25+	25+	419	1,033	
316	Ghana	2012-2014	Research on Obesity and Diabetes among African Migrants (RODAM), control group	Subnational	rural	25+	25+	431	679	
317	Ghana	2013	Silent Crisis: Epidemic Hypertension in Rural West Africa	Subnational	rural	35+	35+	151	278	
318	Ghana	2014	Demographic and Health Survey Ghana 2014	National	both	10+	10+	4,378	9,013	
319	Greece	1991-1999	EPIC	National	both	19-86	19-86	11,120	15,885	
320	Greece	1997	The Didima Study	Community	rural	18+	18+	278	387	
321	Greece	2016	SKG-Elderly	Community	urban	60+	60+	51	63	
322	Greenland	2005-2010	Population Health Survey in Greenland	National	both	18+	18+	1,351	1,725	
323	Grenada	2011	STEPS	National	both	25-64	25-64	440	646	
324	Guatemala	2001-2002	CAMDI	Community	urban	20+	20+	454	940	
325	Guatemala	2003-2005	The Institute of Nutrition of Central America and Panama Nutrition Supplementation Trial Cohort	Community	both	25-41	25-41	265	304	
326	Guinea	2009	STEPS	Subnational	both	15-64	15-64	1,139	1,246	
327	Honduras	2003-2004	CAMDI	Community	urban	20+	20+	437	792	
328	Hungary	1985	INTERSALT	Community	rural	20-59	20-59	100	100	
329	Hungary	2003	The European Male Ageing Study	Community	both	40+		430		
330	Hungary	2008	The European Male Ageing Study	Community	both	40+		349		
331	Iceland	1985-1986	INTERSALT	Community	urban	20-59	20-59	100	100	
332	Iceland	1988-1989	MONICA, Arnes County	Community	rural	25-64	25-64	388	442	
333	Iceland	1988-1989	MONICA, Reykjavik	Subnational	urban	25-64	25-64	413	445	
334	Iceland	1993-1994	MONICA, Reykjavik	Subnational	urban	25-64	25-64	443	449	
335	Iceland	1993-1994	MONICA, Arnes County	Community	rural	25-64	25-64	422	484	
336	Iceland	2002-2006	AGES-Reykjavik Study	Subnational	urban	66-96	66-96	2,420	3,287	
337	Iceland	2005-2011	Risk Evaluation For INfarct Estimates (REFINE)	Subnational	urban	20-73	20-73	3,403	3,527	
338	Iceland	2010-2012	Risk Evaluation For INfarct Estimates (REFINE) follow-up visit (REFINELO)	Subnational	urban	26-74	26-74	654	669	
339	Iceland	2012-2013	Risk Evaluation For INfarct Estimates (REFINE) follow-up visit (REFLOCT)	Subnational	urban	55-73	55-73	516	561	
340	India	1986	INTERSALT	Community	urban	20-59	20-59	100	99	
341	India	1988-1989	Ramachandran et al., Diabetes Res Clin Pract 58:55-60, 2002	Community	urban	20-74	20-74	455	435	
342	India	1992-1994	Jaipur Heart Watch 1	Community	urban	20-80	20-80	1,389	785	
343	India	1992-1994	Jaipur Heart Watch 1	Community	rural	20-80	20-80	1,971	1,159	
344	India	1995	Ramachandran et al., Diabetes Res Clin Pract 42:181-6, 1998	Community	urban	20-74	20-74	1,061	1,093	
345	India	1995-1996	Epidemiology of blood pressure across cross-cultural populations of Visakhapatnam district, Andhra Pradesh, India	Community	rural	19-76	19-76	210	228	
346	India	1995-1997	Aravind Comprehensice Eye Survey	Community	rural	40+	40+	2,200	2,672	

	Country	Data years	Survey/Study name/Citation	Level of representative-ness	Rural, urban or both	Age range as in the NCD-RisC database		Sample size		Note
						Male	Female	Male	Female	
347	India	1997	Ramachandran et al., Diabetes Res Clin Pract 44:207–13, 1999	Community	rural	20-74	20-74	738	879	
348	India	1996-1999	Chennai Urban Population Study	Community	urban	20+	20+	557	705	
349	India	1999-2001	Jaipur Heart Watch 2	Community	urban	20-75	20-75	531	569	
350	India	2001-2004	Chennai Urban Rural Epidemiology Study	Community	urban	20+	20+	1,095	1,252	
351	India	2002-2003	Blood Pressure epidemiology in tribal, rural and urban communities of Orissa with special reference to physical and social parameters	Community	rural	18-80	18-80	201	187	
352	India	2003-2005	WHO-ICMR NCD risk factor surveillance study	National	urban	15-69	15-69	6,553	6,727	
353	India	2003-2005	WHO-ICMR NCD risk factor surveillance study	National	rural	15-69	15-69	6,432	6,842	
354	India	2005-2006	Diet and Nutritional status of Rural population and Prevalnce of Hypertension	National	rural	20+	20+	11,920	13,702	
355	India	2005-2007	Prevalence of cardiovascular risk factors in rural Tamil Nadu	Community	rural	25-64	25-64	4,900	5,563	
356	India	2006	Ramachandran et al., Diabetes Care 31:893-8, 2008	Community	both	20+	20+	3,321	3,745	
357	India	2006-2008	Central India Eye and Medical Study	Community	rural	30+	30+	2,191	2,518	
358	India	2007-2008	SAGE	National	both	50+	50+	3,257	3,201	
359	India	2007-2009	Prevalence of NCD risk factor in people above 15 year in Rural area Nagpur using WHO STEP approach	Community	rural	15+	15+	1,971	1,811	
360	India	2008-2010	ICMR India Diabetes Study	National	both	20+	20+	6,953	7,025	
361	India	2011-2012	Diet and Nutritional status of Rural population and Prevalnce of Hypertension	National	rural	18+	18+	22,075	27,244	
362	India	2012-2014	District Level Household and Facility Survey (DLHS) 4	National	both	18+	18+	402,994	476,021	
363	India	2014	Annual Health Survey-Chemical, Anthropometric	National	both	18+	18+	442,040	479,130	
364	India	2015-2016	Diet and nutritional status of urban population and prevalence of hypertension	National	urban	18+	18+	39,511	54,601	
365	Indonesia	1997-1998	Indonesian Family Life Surveys	National	both	15+	15+	8,714	10,543	
366	Indonesia	2000-2001	Indonesian Family Life Surveys	National	both	15+	15+	11,820	12,745	
367	Indonesia	2007-2008	Indonesian Family Life Surveys	National	both	15+	15+	13,864	15,359	
368	Indonesia	2014-2015	Indonesian Family Life Surveys	National	both	15+	15+	15,284	16,779	
369	Iran	1990-1991	National Health Survey I	National	both	10+	10+	11,849	15,427	
370	Iran	1998	Isfahan Salt Study (ISS)	Community	both	20-60	20-60	421	601	
371	Iran	1999-2000	National Health Survey II	National	both	10+	10+	18,142	21,600	
372	Iran	1999-2001	Tehran Lipid and Glucose Study	Community	both	5+	5+	8,100	8,400	
373	Iran	2001	Isfahan Healthy Heart Program, Najaf Abad rural	Community	rural	19+	19+	408	419	
374	Iran	2001	Isfahan Salt Study (ISS)	Community	both	20-60	20-60	166	181	
375	Iran	2001	Isfahan Healthy Heart Program, Najaf Abad urban	Community	urban	19+	19+	580	576	
376	Iran	2001	Isfahan Healthy Heart Program, Arak urban	Community	urban	19+	19+	2,085	2,127	
377	Iran	2001	Isfahan Healthy Heart Program, Isfahan urban	Community	urban	19+	19+	1,776	1,926	
378	Iran	2001	Isfahan Healthy Heart Program, Isfahan rural	Community	rural	19+	19+	234	239	
379	Iran	2001	Isfahan Healthy Heart Program, Arak rural	Community	rural	19+	19+	1,028	1,090	
380	Iran	2002-2005	Tehran Lipid and Glucose Study	Community	both	5+	5+	2,782	3,560	
381	Iran	2003-2004	The Persian Gulf Healthy Heart Study	Subnational	urban	25-75	25-75	1,740	1,976	
382	Iran	2005	Provincial Non-Communicable Disease Surveillance Survey 2005	National	both	15-64	15-64	40,853	39,823	
383	Iran	2004-2008	Golestan Cohort Study Main Phase	Subnational	rural	40-75	40-75	17,283	22,684	3
384	Iran	2004-2008	Golestan Cohort Study Main Phase	Community	urban	40-75	40-75	3,934	6,098	3
385	Iran	2006	Provincial Non-Communicable Disease Surveillance Survey 2006	National	both	16-65	16-65	14,934	14,636	
386	Iran	2005-2008	Tehran Lipid and Glucose Study	Community	both	5+	5+	3,060	3,808	
387	Iran	2007	Isfahan Healthy Heart Program, Isfahan rural	Community	rural	19+	19+	157	152	
388	Iran	2007	Isfahan Healthy Heart Program, Arak rural	Community	rural	19+	19+	1,030	1,028	
389	Iran	2007	Isfahan Healthy Heart Program, Arak urban	Community	urban	19+	19+	1,429	1,366	
390	Iran	2007	Isfahan Healthy Heart Program, Najaf Abad urban	Community	urban	19+	19+	495	544	
391	Iran	2007	Isfahan Healthy Heart Program, Najaf Abad rural	Community	rural	19+	19+	255	254	
392	Iran	2007	National Non-Communicable Disease Surveillance Survey 2007	National	both	15-64	15-64	2,594	2,528	
393	Iran	2007	Isfahan Healthy Heart Program, Isfahan urban	Community	urban	19+	19+	1,340	1,339	
394	Iran	2007	Isfahan Salt Study (ISS)	Community	both	19-60	19-60	336	437	
395	Iran	2007	Provincial Non-Communicable Disease Surveillance Survey 2007	National	both	15-64	15-64	14,886	14,572	
396	Iran	2008	Provincial Non-Communicable Disease Surveillance Survey 2008	National	both	15-64	15-64	14,845	14,431	

	Country	Data years	Survey/Study name/Citation	Level of representative-ness	Rural, urban or both	Age range as in the NCD-RisC database		Sample size		Note
						Male	Female	Male	Female	
397	Iran	2008-2009	Zahedan city study	Community	urban	10+	10+	1,379	1,204	
398	Iran	2008-2010	Amol county study	Community	both	10+	10+	3,488	2,651	
399	Iran	2008-2010	Tehran city study	Community	urban	10+	10+	426	557	
400	Iran	2009	Provincial Non-Communicable Disease Surveillance Survey 2009	National	both	15-64	15-64	14,903	14,545	
401	Iran	2008-2011	Tehran Lipid and Glucose Study	Community	urban	20+	20+	4,713	6,024	
402	Iran	2009-2010	The Persian Gulf Healthy Heart Study	Subnational	urban	31-79	31-79	835	1,016	
403	Iran	2010-2012	Golestan Cohort Study Second Phase	Subnational	rural	43-82	43-82	4,325	4,920	3
404	Iran	2010-2012	Golestan Cohort Study Second Phase	Community	urban	43-82	43-82	1,089	1,062	3
405	Iran	2011	Provincial Non-Communicable Disease Surveillance Survey 2011	National	both	10-69	10-69	4,722	6,387	
406	Iran	2013-2014	Isfahan Salt Study (ISS)	Community	both	10-60	10-60	649	715	
407	Iran	2013-2014	Bushehr Elderly Health Program (BEH)	Community	urban	60+	60+	1,455	1,544	
408	Iraq	2015	STEPS	National	both	18+	18+	1,593	2,326	
409	Ireland	1998	Survey of Lifestye, Attitudes and Nutritional in Ireland 1998	National	both	18+	18+	125	296	
410	Ireland	2002	Survey of Lifestye, Attitudes and Nutritional in Ireland 2002	National	both	18+	18+	169	221	
411	Ireland	2006-2007	Survey of Lifestye, Attitudes and Nutritional in Ireland 2006-2007	National	both	45+	45+	526	679	
412	Ireland	2008-2010	National Adult Nutrition Survey	National	both	18+	18+	644	671	
413	Ireland	2009-2011	The Irish Longitudinal Study on Ageing	National	both	50+	50+	2,696	3,173	
414	Israel	1999-2005	The Israel Glucose Intolerance, Obesity and Hypertention Study	National	urban	58+	58+	513	505	
415	Israel	2002-2007	Hadera District Study	Subnational	urban	25-78	25-78	388	377	
416	Israel	2005-2006	Mabat Zahav National Health and Nutrition Survey ages 65 and over 2005-6	National	urban	65+	65+	793	890	
417	Italy	1985	INTERSALT, Naples	Community	urban	20-59	20-59	100	100	
418	Italy	1985	Finland, Italy, Netherlands, Elderly (Fine-Italy)	Community	rural	65-84		680		
419	Italy	1986	INTERSALT, Gubbio	Community	urban	20-59	20-59	99	100	
420	Italy	1986	INTERSALT, Bassiano	Community	urban	20-59	20-59	99	100	
421	Italy	1986	INTERSALT, Mirano	Community	urban	20-59	20-59	100	100	
422	Italy	1986	MONICA, Friuli	Subnational	urban	25-64	25-64	925	919	
423	Italy	1986-1987	MONICA, Brianza	Subnational	urban	25-64	25-64	809	830	
424	Italy	1989	MONICA, Friuli	Subnational	urban	25-64	25-64	906	906	
425	Italy	1989	Ventimiglia Heart Study	Community	rural	10+	10+	578	690	
426	Italy	1983-1996	Malattie cardiovascolari ATerosclerotiche Istituto Superiore di Sanità	Community	rural	18-77	18-77	3,968	4,519	
427	Italy	1989-1990	MONICA, Brianza	Subnational	urban	25-64	25-64	764	762	
428	Italy	1990	Bruneck Study	Community	rural	40-79	40-79	469	450	
429	Italy	1992-1993	Italian Longitudinal Study on Aging	National	both	65-84	65-84	1,828	1,669	
430	Italy	1993-1994	MONICA, Brianza	Subnational	urban	25-64	25-64	796	848	
431	Italy	1994	MONICA, Friuli	Subnational	urban	25-64	25-64	883	888	
432	Italy	1995	Bruneck Study	Community	rural	45-84	45-84	414	412	
433	Italy	1993-1998	EPIC Florence	Community	urban	24-72	24-72	3,327	9,486	
434	Italy	1995-1996	Italian Longitudinal Study on Aging	National	both	69-90	69-90	1,091	972	
435	Italy	1995-1999	PROgetto Veneto Anziani	Subnational	both	65+	65+	1,243	1,851	
436	Italy	1998-1999	progetto VIP	Community	both	25-74	25-74	598	599	
437	Italy	1998-2002	Osservatorio Epidemiologico Cardiovascolare	National	both	35-74	35-74	4,878	4,772	
438	Italy	2000	Bruneck Study	Community	rural	50-89	50-89	331	361	
439	Italy	2000-2003	PROgetto Veneto Anziani	Subnational	both	67+	67+	804	1,363	
440	Italy	2001-2003	The Study of Asti	Community	both	45-64	45-64	780	878	
441	Italy	2003	The European Male Ageing Study	Community	both	40+		433		
442	Italy	2002-2005	PROgetto Veneto Anziani	Subnational	both	68+	68+	626	1,147	
443	Italy	2004-2005	Italian Project on the Epidemiolofy of Alzheimer's disease	National	both	65-84	65-84	1,578	1,440	
444	Italy	2004-2005	Vobarno study	Community	rural	55-74	55-74	99	113	
445	Italy	2005	Bruneck Study	Community	rural	55-93	55-93	264	307	
446	Italy	2005-2007	Moli-family Study	Subnational	both	14-35	14-35	129	158	
447	Italy	2005-2010	Moli-sani Study	Subnational	both	35+	35+	11,699	12,617	

	Country	Data years	Survey/Study name/Citation	Level of representative-ness	Rural, urban or both	Age range as in the NCD-RisC database		Sample size		Note
						Male	Female	Male	Female	
448	Italy	2008	The European Male Ageing Study	Community	both	40+		352		
449	Italy	2008-2009	progetto VIP	Community	both	25-74	25-74	600	598	
450	Italy	2008-2012	Osservatorio Epidemiologico Cardiovascolare/Health Examination Survey	National	both	35-80	35-80	4,370	4,337	
451	Italy	2010	Bruneck Study	Community	rural	60-98	60-98	224	256	
452	Italy	2010-2012	CArdiovascular risk MEtabolic syndrome LIver and Autoimmunity diseases (CA.ME.LI.A)	Community	both	18-75	18-75	477	515	
453	Italy	2011-2012	Vobarno study	Community	rural	49-62	49-62	104	143	
454	Italy	2014-2016	Mediterranean healthy Eating, Aging and Lifestyles (MEAL) study	Subnational	urban	20+	20+	750	1,117	
455	Italy	2015	Bruneck Study	Community	rural	65-98	65-98	171	169	
456	Jamaica	2000-2001	Jamaica Health and Lifestyle Survey	National	both	15-74	15-74	656	1,291	
457	Jamaica	2007-2008	Jamaica Health and Lifestyle Survey	National	both	15-74	15-74	869	1,926	
458	Japan	1985	INTERSALT, Osaka	Community	urban	20-59	20-59	100	97	
459	Japan	1985	National Nutrition Survey	National	both	15+	15+	5,364	7,154	
460	Japan	1985	INTERSALT, Toyama	Community	urban	20-59	20-59	100	100	
461	Japan	1985	INTERSALT, Tochigi	Community	urban	20-59	20-59	95	99	
462	Japan	1985-1986	Akabane Study	Community	urban	40-69	40-69	813	1,023	
463	Japan	1986	National Nutrition Survey	National	both	15+	15+	5,471	7,050	
464	Japan	1987	Konan Town Study	Community	rural	20-79	20-79	70	89	
465	Japan	1987	National Nutrition Survey	National	both	15+	15+	4,485	6,485	
466	Japan	1988	National Nutrition Survey	National	both	15+	15+	4,528	6,254	
467	Japan	1988-1990	Miyama Cohort Study	Community	rural	40-80	40-80	477	601	
468	Japan	1989	National Nutrition Survey	National	both	15+	15+	4,066	5,548	
469	Japan	1990	National Nutrition Survey	National	both	15+	15+	4,448	5,988	
470	Japan	1991	Shigaraki Town Study	Community	rural	30-89	30-89	234	330	
471	Japan	1991	Konan Town Study	Community	rural	20-79	20-79	93	117	
472	Japan	1991	National Nutrition Survey	National	both	15+	15+	4,237	5,706	
473	Japan	1992	Shigaraki Town Study	Community	rural	30-89	30-89	288	387	
474	Japan	1992	National Nutrition Survey	National	both	15+	15+	3,879	5,307	
475	Japan	1993	Shigaraki Town Study	Community	rural	30-89	30-89	301	454	
476	Japan	1993	National Nutrition Survey	National	both	15+	15+	3,492	4,921	
477	Japan	1994	National Nutrition Survey	National	both	15+	15+	3,342	4,783	
478	Japan	1994	Shigaraki Town Study	Community	rural	30-89	30-89	252	336	
479	Japan	1995	National Nutrition Survey	National	both	15+	15+	3,269	4,654	
480	Japan	1995	Shigaraki Town Study	Community	rural	30-89	30-89	300	470	
481	Japan	1996	National Nutrition Survey	National	both	15+	15+	3,152	4,476	
482	Japan	1996	Shigaraki Town Study	Community	rural	30-89	30-89	86	152	
483	Japan	1996-1997	INTERMAP, AitoTown	Community	both	40-59	40-59	130	129	
484	Japan	1997	National Nutrition Survey	National	both	15+	15+	3,069	4,386	
485	Japan	1997	Shigaraki Town Study	Community	rural	30-89	30-89	61	100	
486	Japan	1997-1998	INTERMAP, Wakayama	Community	urban	40-59	40-59	146	144	
487	Japan	1997-1998	INTERMAP, Toyama	Community	urban	40-59	40-59	149	150	
488	Japan	1997-1998	INTERMAP, Sapporo	Community	urban	40-59	40-59	149	148	
489	Japan	1998	National Nutrition Survey	National	both	15+	15+	3,281	4,681	
490	Japan	1999	National Nutrition Survey	National	both	15+	15+	2,550	3,814	
491	Japan	2000	National Nutrition Survey	National	both	15+	15+	2,690	3,788	
492	Japan	2001	National Nutrition Survey	National	both	15+	15+	2,465	3,754	
493	Japan	2001	The Japan Association of Health Service Database	Subnational	both	20+	20+	1,318,133	1,061,237	
494	Japan	2002	National Nutrition Survey	National	both	15+	15+	2,394	3,473	
495	Japan	2002-2003	The Hisayama Study	Community	rural	40+	40+	1,414	1,883	
496	Japan	2003	National Health and Nutrition Survey	National	both	15+	15+	2,350	3,491	
497	Japan	2004	National Health and Nutrition Survey	National	both	15+	15+	1,743	2,590	
498	Japan	2005	National Health and Nutrition Survey	National	both	15+	15+	1,723	2,496	

	Country	Data years	Survey/Study name/Citation	Level of representative-ness	Rural, urban or both	Age range as in the NCD-RisC database		Sample size		Note
						Male	Female	Male	Female	
499	Japan	2006	National Health and Nutrition Survey	National	both	15+	15+	1,925	2,787	
500	Japan	2007	National Health and Nutrition Survey	National	both	15+	15+	1,810	2,634	
501	Japan	2008	National Health and Nutrition Survey	National	both	15+	15+	1,981	2,822	
502	Japan	2009	National Health and Nutrition Survey	National	both	15+	15+	1,887	2,726	
503	Japan	2010	National Health and Nutrition Survey	National	both	15+	15+	1,758	2,470	
504	Japan	2011	National Health and Nutrition Survey	National	both	15+	15+	1,652	2,326	
505	Japan	2011	The Tokyo Health Service Association Database	Community	urban	20+	20+	82,375	53,738	
506	Japan	2012	National Health and Nutrition Survey	National	both	15+	15+	6,163	8,875	
507	Japan	2013	National Health and Nutrition Survey	National	both	15+	15+	1,487	2,061	
508	Japan	2014	National Health and Nutrition Survey	National	both	15+	15+	1,555	2,141	
509	Japan	2014-2015	Nagaoka Health Screening	Community	both	20-89	20-89	4,938	4,298	
510	Japan	2015	National Health and Nutrition Survey	National	both	15+	15+	1,433	2,091	
511	Jordan	2004	Behavioural Risk Factor Surveillence Survey	National	both	18+	18+	237	474	
512	Jordan	2007	Behavioural Risk Factor Surveillence Survey	National	both	18+	18+	332	433	
513	Jordan	2009	Metablic abnomalities and vitamin D study	National	both	10+	10+	1,496	3,727	
514	Jordan	2016-2017	National Cardiovascular Diseases and Diabetes Study (NCDDS)	National	both	18+	18+	1,186	2,731	
515	Kazakhstan	2015	Shymkent STEPS	Subnational	both	18-69	18-69	362	701	
516	Kazakhstan	2015	Almaty STEPS	Subnational	both	18-69	18-69	384	1,145	
517	Kazakhstan	2015-2016	Aktobe STEPS	Subnational	both	18-69	18-69	346	1,156	
518	Kenya	2015	STEPS	National	both	18-69	18-69	1,761	2,526	
519	Kiribati	2004	STEPS	National	both	15-64	15-64	784	945	
520	Kuwait	2006	STEPS	National	both	20-65	20-65	918	1,297	
521	Kuwait	2008-2010	Gulf Cooperation Council World Health Survey	National	both	18+	18+	1,624	1,851	
522	Kuwait	2014	STEPS	National	both	18-69	18-69	1,380	2,216	
523	Kyrgyzstan	2012	Demographic and Health Survey Kyrgyzstan 2012	National	both	15-49	15-49	2,412	7,604	
524	Kyrgyzstan	2013	STEPS	National	both	25-64	25-64	943	1,601	
525	Lao PDR	2008	STEPS	Community	urban	25-64	25-64	1,668	2,455	
526	Lao PDR	2013	STEPS	National	both	18-64	18-64	988	1,469	
527	Lebanon	2008-2009	STEPS	National	both	18+	18+	1,319	1,480	
528	Lesotho	2009-2010	Demographic and Health Survey Lesotho 2009-2010	National	both	15-59	15-49	3,166	3,732	
529	Lesotho	2012	STEPS	National	both	25-64	25-64	743	1,457	
530	Lesotho	2014	Demographic and Health Survey Lesotho 2014	National	both	15-59	15-49	2,796	3,165	
531	Liberia	2011	STEPS	National	both	25-64	25-64	1,017	1,270	
532	Libya	2009	STEPS	National	both	25-64	25-64	1,684	1,569	
533	Lithuania	1986-1987	MONICA, Kaunas	Community	urban	35-64	35-64	894	868	
534	Lithuania	1987	Countrywide Integrated Noncommunicable Diseases Intervention Programme survey	Subnational	rural	25-64	25-64	1,227	1,445	
535	Lithuania	1992-1993	MONICA, Kaunas	Community	urban	35-64	35-64	611	628	
536	Lithuania	1992-1993	Countrywide Integrated Noncommunicable Diseases Intervention Programme survey	Subnational	rural	25-64	25-64	677	864	
537	Lithuania	1998-1999	Countrywide Integrated Noncommunicable Diseases Intervention Programme survey	Subnational	rural	25-64	25-64	816	1,018	
538	Lithuania	2001-2002	MONICA4	Community	urban	35-64	35-64	626	775	
539	Lithuania	2006-2007	Countrywide Integrated Noncommunicable Diseases Intervention Programme survey	Subnational	rural	25-64	25-64	731	982	
540	Lithuania	2006-2008	Health, Alcohol and Psychosocial factors In Eastern Europe	Community	urban	45-75	45-75	3,220	3,863	
541	Luxembourg	2007-2009	Observation des Risques et de la Santé Cardio-Vasculaire au Luxembourg (ORISCAV-LUX)	National	both	18-69	18-69	696	734	
542	Madagascar	2005	STEPS	Community	urban	25-64	25-64	2,745	2,682	
543	Malawi	2009	STEPS	National	both	25-64	25-64	1,183	2,527	
544	Malaysia	1996	National Health and Morbidity Survey (NHMS)	National	both	18+	18+	10,123	11,532	
545	Malaysia	2004	Rampal et al., Public Health 2008; 122: 11-8	National	both	15+	15+	7,301	9,899	
546	Malaysia	2006	National Health and Morbidity Survey (NHMS)	National	both	18+	18+	15,208	18,774	
547	Malaysia	2008	Metabolic Syndrome Study in Malaysia	National	urban	18+	18+	769	1,449	
548	Malaysia	2008	Metabolic Syndrome Study in Malaysia	National	rural	18+	18+	754	1,368	
549	Malaysia	2011	National Health and Morbidity Survey (NHMS)	National	both	18+	18+	8,235	9,450	

	Country	Data years	Survey/Study name/Citation	Level of representative-ness	Rural, urban or both	Age range as in the NCD-RisC database		Sample size		Note
						Male	Female	Male	Female	
550	Malaysia	2015	National Health and Morbidity Survey (NHMS)	National	both	18+	18+	9,067	10,100	
551	Maldives	2011	STEPS	National	both	15-64	15-64	661	1,067	
552	Mali	2007	STEPS	Community	both	15-64	15-64	1,060	1,539	
553	Malta	1986	INTERSALT	Community	rural	20-59	20-59	100	100	
554	Marshall Islands	2002	STEPS	National	both	15-64	15-64	785	1,196	
555	Mauritius	1987	Mauritius non communicable disease survey	National	both	25-74	25-74	2,351	2,659	
556	Mauritius	1992	Mauritius non communicable disease survey	National	both	25-74	25-74	2,983	3,475	
557	Mauritius	1998	Mauritius non communicable disease survey	National	both	25-74	25-74	2,560	3,243	
558	Mauritius	2009	Mauritius non communicable diseases survey	National	both	19+	19+	2,901	3,430	
559	Mexico	1992-1993	Encuesta Nacional de Enfermedades Cronicas	National	urban	20-69	20-69	6,115	8,351	
560	Mexico	1999-2000	The Survey on Health, Well-Being, and Aging in Latin America and the Caribbean	Community	urban	60+	60+	423	645	2
561	Mexico	2000	Encuesta Nacional de Salud	National	both	20+	20+	13,689	28,706	
562	Mexico	2002	Encuesta Nacional Sobre Niveles de vida de los Hogares	National	both	15+	15+	7,371	9,143	
563	Mexico	2005	Encuesta Nacional Sobre Niveles de vida de los Hogares	National	both	10+	10+	8,398	9,994	
564	Mexico	2006	Encuesta Nacional de Salud Y Nutricion	National	both	20+	20+	13,358	20,008	
565	Mexico	2006	PREVENIMSS National Coverage Surveys	Subnational	both	20+	20+	8,722	11,333	
566	Mexico	2009-2010	SAGE	National	both	50+	50+	834	1,298	
567	Mexico	2009-2012	Encuesta Nacional Sobre Niveles de vida de los Hogares	National	both	15+	15+	9,886	11,905	
568	Mexico	2011-2012	Encuesta Nacional de Salud Y Nutricion	National	both	20+	20+	4,635	6,716	
569	Mexico	2012	The Mexican Health and Aging Study	National	both	50+	50+	803	1,126	
570	Mexico	2016	Encuesta Nacional de Salud Y Nutricion	National	both	20+	20+	2,852	5,444	
571	Micronesia (Federated S	2002	STEPS	Subnational	both	25-64	25-64	637	948	
572	Micronesia (Federated S	2006	STEPS	Subnational	both	15-64	15-64	925	1,557	
573	Micronesia (Federated S	2008	STEPS	Subnational	both	25-64	25-64	897	1,292	
574	Micronesia (Federated S	2009	STEPS	Subnational	both	15-64	15-64	213	429	
575	Micronesia (Federated S	2009	STEPS	Subnational	both	15-64	15-64	435	549	
576	Moldova	2013	STEPS	National	both	18-69	18-69	1,729	2,809	
577	Mongolia	2005	STEPS	National	both	15-64	15-64	1,664	1,713	
578	Mongolia	2009	STEPS	National	both	15-64	15-64	2,212	3,117	
579	Mongolia	2013	STEPS	National	both	15-64	15-64	2,711	3,168	
580	Mozambique	2005	STEPS	National	both	25-64	25-64	1,280	1,687	
581	Mozambique	2014-2015	STEPS	National	both	15-64	15-64	1,151	1,693	
582	Myanmar	2003-2004	STEPS	Subnational	both	25-74	25-74	1,989	2,446	
583	Myanmar	2009	STEPS	National	both	15-64	15-64	2,858	4,451	
584	Myanmar	2013-2014	Non-communicable Disease Risk Factor Survey	Subnational	both	25-74	25-74	745	740	
585	Namibia	2005	STEPS	National	both	24-64	24-64	1,403	1,795	
586	Namibia	2009	Okambilimbili Survey	Community	urban	12+	12+	973	1,189	
587	Namibia	2013	Demographic and Health Survey Namibia 2013	National	both	15-64	15-64	1,539	2,042	
588	Nauru	1987	Trends in the prevalence and incidence of non-insulin-dependent diabetes mellitus and impaired glucose tolerance	National	both	20+	20+	553	666	
589	Nauru	1994	Trends in the prevalence and incidence of non-insulin-dependent diabetes mellitus and impaired glucose tolerance	National	both	25+	25+	648	726	
590	Nauru	2004	STEPS	National	both	15-64	15-64	1,085	1,148	
591	Nauru	2006	STEPS	National	both	16-65	16-65	257	238	
592	Nepal	2005	Noncommunicable Disease Risk Factors Survey; STEPS	Subnational	both	15-64	15-64	3,667	4,025	
593	Nepal	2007-2008	Noncommunicable Disease Risk Factors Survey; STEPS	National	both	15-64	15-64	1,905	2,372	
594	Nepal	2006-2011	Early detection and management of Kidney disease, Hypertension, Diabetes and Cardiovascular disease (KHDC Nepal), Damak	Community	urban	18+	18+	1,095	1,577	
595	Nepal	2006-2011	Early detection and management of Kidney disease, Hypertension, Diabetes and Cardiovascular disease (KHDC Nepal), Dharan	Community	urban	18+	18+	4,130	6,127	
596	Nepal	2006-2011	Early detection and management of Kidney disease, Hypertension, Diabetes and Cardiovascular disease (KHDC Nepal), Tarahara	Community	rural	18+	18+	1,176	2,351	
597	Nepal	2013	STEPS	National	both	15-69	15-69	1,326	2,763	

	Country	Data years	Survey/Study name/Citation	Level of representative-ness	Rural, urban or both	Age range as in the NCD-RisC database		Sample size		Note
						Male	Female	Male	Female	
598	Netherlands	1985	INTERSALT	Community	urban	20-59	20-59	100	99	
599	Netherlands	1985	Zutphen Elderly Study	Community	urban	65-85		886		
600	Netherlands	1990	Zutphen Elderly Study	Community	urban	69-90		554		
601	Netherlands	1989-1993	the Rotterdam Study, first subcohort	Community	urban	55+	55+	2,797	4,212	
602	Netherlands	1992-1993	The Longitudinal Aging Study Amsterdam (LASA)	Subnational	both	55-85	55-85	1,270	1,353	4
603	Netherlands	1993-1995	the Rotterdam Study, first subcohort	Community	urban	56+	56+	2,305	3,278	
604	Netherlands	1993-1997	EPIC Utrecht	Community	both		49-70		16,991	
605	Netherlands	1993-1997	EPIC Bilthoven	Community	urban	20-59	20-59	9,662	11,653	
606	Netherlands	1995-1996	The Longitudinal Aging Study Amsterdam (LASA)	Subnational	both	58-88	58-88	722	751	4
607	Netherlands	1997-1999	the Rotterdam Study, first subcohort	Community	urban	61+	61+	1,741	2,428	
608	Netherlands	1998-1999	The Longitudinal Aging Study Amsterdam (LASA)	Subnational	both	61-91	61-91	616	741	4
609	Netherlands	1998-2001	Regenboog Project	National	both	12-89	12-89	2,713	2,636	
610	Netherlands	2000-2001	the Rotterdam Study, second subcohort	Community	urban	55+	55+	1,212	1,472	
611	Netherlands	2001-2002	The Longitudinal Aging Study Amsterdam (LASA)	Subnational	both	64-94	64-94	587	686	4
612	Netherlands	2001-2003	Surinamese in the Netherlands: Study on Ethnicity and Health (SUNSET)	Community	urban	35-60	35-60	251	257	
613	Netherlands	2002-2004	the Rotterdam Study, first subcohort	Community	urban	65+	65+	1,287	1,852	
614	Netherlands	2004-2005	the Rotterdam Study, second subcohort	Community	urban	58+	58+	980	1,267	
615	Netherlands	2005-2006	The Longitudinal Aging Study Amsterdam (LASA)	Subnational	both	57-97	57-97	806	976	4
616	Netherlands	2006-2008	the Rotterdam Study, third subcohort	Community	urban	45+	45+	1,574	2,068	
617	Netherlands	2008-2009	The Longitudinal Aging Study Amsterdam (LASA)	Subnational	both	60-100	60-100	663	813	4
618	Netherlands	2009-2010	Measuring the Netherlands (NL de Maat)	Subnational	both	30-70	30-70	1,779	2,019	
619	Netherlands	2011-2012	the Rotterdam Study, second subcohort	Community	urban	65+	65+	720	913	
620	Netherlands	2011-2012	The Longitudinal Aging Study Amsterdam (LASA)	Subnational	both	63-104	63-104	541	660	4
621	Netherlands	2011-2015	Healthy Life in an Urban Setting (HELIUS)	Community	urban	18-71	18-71	2,085	2,470	
622	Netherlands	2012-2014	the Rotterdam Study, third subcohort	Community	urban	51+	51+	1,254	1,635	
623	New Zealand	1993-1994	MONICA, Auckland	Community	urban	35-64	35-64	745	726	
624	New Zealand	2008-2009	2008/09 New Zealand Adult Nutrition Survey	National	both	15+	15+	1,949	2,396	
625	New Zealand	2012-2013	2012/13 New Zealand Health Survey	National	both	15+	15+	4,952	6,692	
626	Nicaragua	2003-2004	CAMDI	Community	urban	20+	20+	774	916	
627	Niger	2007	STEPS	National	both	15-64	15-64	1,447	1,225	
628	Nigeria	2007	Ibadan Study of Ageing	Subnational	both	60+	60+	670	928	
629	Nigeria	2008	Ibadan Study of Ageing	Subnational	both	61+	61+	479	662	
630	Nigeria	2009	Community Health Plan - Kwara Central Survey	Community	rural	12+	12+	1,642	1,777	
631	Nigeria	2009	Ibadan Study of Ageing	Subnational	both	62+	62+	450	616	
632	Nigeria	2011	Community Health Plan - Kwara Central Survey	Community	rural	12+	12+	591	636	
633	Nigeria	2013	Community Health Plan - Kwara Central Survey	Community	rural	12+	12+	530	583	
634	Norway	1984-1986	HUNT1 Study	Subnational	rural	20+	20+	36,655	38,145	
635	Norway	1986-1987	The Tromsø Study: Tromsø 3	Community	both	20-61	20-56	10,372	9,820	
636	Norway	1994-1995	The Tromsø Study: Tromsø 4	Community	both	25+	25+	12,775	13,864	
637	Norway	1995-1997	HUNT2 study	Subnational	rural	20+	20+	30,297	33,836	
638	Norway	2001-2002	The Tromsø Study: Tromsø 5, Tromsø Study Panel	Community	both	30-89	30-89	2,542	3,594	
639	Norway	2006-2008	HUNT3 Study	Subnational	rural	20+	20+	20,011	24,068	
640	Norway	2007-2008	The Tromsø Study: Tromsø 6	Community	both	30-87	30-87	6,014	6,868	
641	Occupied Palestinian Te	1996-1998	Old Ramallah, urban	Community	urban	15-64	15-64	182	494	
642	Occupied Palestinian Te	1996-1998	Kobar, rural	Community	rural	15-64	15-64	206	483	
643	Occupied Palestinian Te	1999-2000	the First National Health and Nutrition Survey	National	both	18-64	18-64	1,600	1,704	
644	Occupied Palestinian Te	2010	STEPS	National	both	15-64	15-64	2,566	4,086	
645	Oman	2008	Gulf Cooperation Council World Health Survey	National	both	18+	18+	2,421	2,234	
646	Pakistan	1990-1994	National Health Survey Of Pakistan 1990-1994	National	both	10+	10+	5,642	6,070	
647	Pakistan	2005	STEPS	National	both	25-65	25-65	800	1,100	
648	Pakistan	2014	STEPS	Subnational	both	18-69	18-69	2,988	3,703	

	Country	Data years	Survey/Study name/Citation	Level of representative-ness	Rural, urban or both	Age range as in the NCD-RisC database		Sample size		Note
						Male	Female	Male	Female	
649	Palau	2011-2013	STEPS	National	both	25-64	25-64	1,048	1,133	
650	Panama	2010-2011	Prevalencia de factores de riesgo asociados a enfermedad cardiovascular 2010-2011	Subnational	both	18+	18+	1,073	2,474	
651	Papua New Guinea	1985-1986	INTERSALT	Community	rural	20-59	20-59	88	74	
652	Papua New Guinea	2007	STEPS	National	both	15-64	15-64	1,411	1,448	
653	Peru	2004-2005	Encuesta Nacional de Indicadores Nutricionales, BioquC-micos, SocioeconC3micos y Culturales Relacionados con las Enfermedades CrC3nicas Degenerativas (ENIN)	National	both	20+	20+	2,090	2,103	
654	Peru	2007-2008	PERU MIGRANT Study	Community	both	35+	35+	406	442	
655	Peru	2010	Demographic and Health Survey Peru 2010	National	both	50+	15+	7,735	31,501	
656	Peru	2009-2012	CRONICAS Cohort Study	Subnational	both	35+	35+	1,557	1,659	
657	Peru	2011	Demographic and Health Survey Peru 2011	National	both	40+	15+	12,894	14,866	
658	Peru	2012	Demographic and Health Survey Peru 2012	National	both	40+	15+	14,278	15,928	
659	Peru	2013	DHS	National	both	15+	15+	15,853	17,680	
660	Peru	2014	DHS	National	both	15+	15+	12,758	14,414	
661	Peru	2015	DHS	National	both	15+	15+	14,798	17,921	
662	Philippines	1998-1999	Cebu Longitudinal Health and Nutrition Survey 1998-1999 Mother Followup	Community	both		15-59		1,913	
663	Philippines	2002	Cebu Longitudinal Health and Nutrition Survey 2002 Mother Followup	Community	both		32-66		2,080	
664	Philippines	2005	Cebu Longitudinal Health and Nutrition Survey 2005 Mother Followup	Community	both		35-69		2,000	
665	Philippines	2007	Cebu Longitudinal Health and Nutrition Survey 2007 Mother Followup	Community	both		38-71		1,974	
666	Poland	1986	INTERSALT, Krakow	Community	urban	20-59	20-59	100	100	
667	Poland	1986	INTERSALT, Warsaw	Community	urban	20-59	20-59	100	100	
668	Poland	1987-1988	MONICA, Tarnobrzeg Voivodship	Community	rural	35-64	35-64	616	672	
669	Poland	1988-1989	MONICA, Warsaw	Community	urban	35-64	35-64	705	713	
670	Poland	1989-1990	Polish Program CINDI (CINDI Lodz 1989-1990)	Community	urban	25-64	25-64	822	948	
671	Poland	1992-1993	MONICA, Tarnobrzeg Voivodship	Community	rural	35-64	35-64	621	696	
672	Poland	1993	MONICA, Warsaw	Community	urban	35-64	35-64	751	763	
673	Poland	1995-1996	Polish Program CINDI (CINDI Lodz 1995)	Community	urban	17-64	17-64	987	1,465	
674	Poland	2000	The health status, risk factors of chronic diseases and health behaviors of residents of Torun (CINDI Torun 2000)	Community	urban	16-83	16-83	988	1,057	
675	Poland	2001-2002	The health status, risk factors of chronic diseases and health behaviors of residents of Lodz (CINDI Lodz 2001)	Community	urban	18-64	18-64	997	838	
676	Poland	2002	The health status, risk factors of chronic diseases and health behaviors of residents of Lodz - seniors (CINDI Lodz 2002)	Community	urban	65+	65+	291	538	
677	Poland	2002	NATPOL	National	both	18+	18+	1,017	1,302	
678	Poland	2003	The European Male Ageing Study	Community	both	40+		407		
679	Poland	2002-2005	Health, Alcohol and Psychosocial factors In Eastern Europe	Community	urban	45-69	45-69	4,454	4,715	
680	Poland	2003-2005	National Multicenter Health Survey in Poland. Project WOBASZ	National	both	20-74	20-74	6,311	6,967	
681	Poland	2006	The health, risk factors for chronic diseases, attitudes and behaviors of health residents of Torun (CINDI Torun 2006)	Community	urban	15-65	15-65	790	1,146	
682	Poland	2003-2013	Mogielica Human Ecology Study	Community	rural	21+	21+	349	893	
683	Poland	2008	The European Male Ageing Study	Community	both	40+		311		
684	Poland	2007-2011	Medical, psychological and socioeconomic aspects of aging in Poland (PolSenior)	National	both	55+	55+	2,877	2,756	
685	Poland	2011	NATPOL	National	both	18-79	18-79	1,168	1,244	
686	Poland	2013-2014	National Multicenter Health Survey in Poland. Project WOBASZ II	National	both	20+	20+	2,752	3,371	
687	Poland	2015-2016	LIPIDOGRAM2015 & LIPIDOGEN2015 Study - National epidemiological study of lipid disorders and selected risk factors of cardiovascular disease in primary health care in Poland	National	both	18+	18+	5,034	8,690	
688	Portugal	1986	INTERSALT	Community	both	20-59	20-59	99	99	
689	Portugal	1999-2003	EPIPorto Study	Community	urban	18+	18+	888	1,424	
690	Portugal	2010-2012	Exercise for Elderly	Community	urban	60-84	60-84	47	104	
691	Qatar	2006	World Health Survey	National	both	18+	18+	1,885	2,043	
692	Qatar	2012	STEPS	National	both	18-64	18-64	1,039	1,355	
693	Romania	2011-2012	Study for the Evaluation of Prevalence of Hypertension and cArdiovascular Risk among the Adult Population of Romania - SEPHAR II	National	both	18-80	18-80	1,038	936	

	Country	Data years	Survey/Study name/Citation	Level of representative-ness	Rural, urban or both	Age range as in the NCD-RisC database		Sample size		Note
						Male	Female	Male	Female	
694	Romania	2015-2016	Study for the Evaluation of Prevalence of Hypertension and cArdiovascular Risk among the Adult Population of Romania - SEPHAR III	National	both	18-80	18-80	936	1,034	
695	Russian Federation	1984-1986	MONICA, Moscow, Cheremushkinsky district	Community	urban	35-64	35-64	600	600	
696	Russian Federation	1984-1986	MONICA, Moscow (control)	Community	urban	35-64	35-64	774	642	
697	Russian Federation	1984-1986	MONICA, Moscow, Leninsky district	Community	urban	35-64	35-64	553	622	
698	Russian Federation	1985	MONICA, Novosibirsk (intervention)	Community	urban	25-64	25-64	797	818	
699	Russian Federation	1985-1986	MONICA, Novosibirsk, Kirowsky district	Community	urban	25-64	25-64	758	774	
700	Russian Federation	1985-1986	MONICA, Novosibirsk, Leninsky district	Community	urban	25-64	25-64	624	628	
701	Russian Federation	1986	INTERSALT	Community	urban	20-59	20-59	97	97	
702	Russian Federation	1988	MONICA, Novosibirsk (intervention)	Community	urban	25-64	25-64	840	853	
703	Russian Federation	1988-1989	MONICA, Moscow (control)	Community	urban	35-64	35-64	621	581	
704	Russian Federation	1988-1989	MONICA, Moscow, Leninsky district	Community	urban	35-64	35-64	597	613	
705	Russian Federation	1988-1989	MONICA, Novosibirsk, Kirowsky district	Community	urban	25-64	25-64	876	805	
706	Russian Federation	1992	Russian Karelia Survey in Pitkaranta	Community	both	25-64	25-64	380	454	
707	Russian Federation	1992-1993	Russia Longitudinal Monitoring Survey-Higher School of Economics Round II	National	both	10+	10+	4,799	6,411	
708	Russian Federation	1992-1995	MONICA, Moscow, Leninsky district	Community	urban	35-64	35-64	537	858	
709	Russian Federation	1992-1995	MONICA, Moscow (control)	Community	urban	35-64	35-64	557	527	
710	Russian Federation	1993	Russia Longitudinal Monitoring Survey-Higher School of Economics Round III	National	both	10+	10+	5,420	7,191	
711	Russian Federation	1993-1994	Russia Longitudinal Monitoring Survey-Higher School of Economics Round IV	National	both	10+	10+	4,985	6,597	
712	Russian Federation	1994-1995	MONICA, Novosibirsk (intervention)	Community	urban	25-64	25-64	822	865	
713	Russian Federation	1995	MONICA, Novosibirsk, Kirowsky district	Community	urban	25-64	25-64	771	787	
714	Russian Federation	1997	Russian Karelia Survey in Pitkaranta	Community	both	25-64	25-64	309	440	
715	Russian Federation	2002	Russian Karelia Survey in Pitkaranta	Community	both	25-64	25-64	258	342	
716	Russian Federation	2002-2005	Health, Alcohol and Psychosocial factors In Eastern Europe	Community	urban	45-69	45-69	4,205	5,039	
717	Russian Federation	2007	Russian Karelia Survey in Pitkaranta	Community	both	25-64	25-64	176	277	
718	Russian Federation	2007-2010	SAGE	National	both	50+	50+	1,350	2,464	
719	Russian Federation	2015-2016	Ural Eye and Medical Study (UEMS)	Subnational	rural	40+	40+	816	1,371	
720	Russian Federation	2015-2016	Ural Eye and Medical Study (UEMS)	Subnational	urban	40+	40+	706	1,353	
721	Rwanda	2012	STEPS	National	both	15-64	15-64	2,650	4,244	
722	Saint Kitts and Nevis	2007	STEPS	Subnational	both	25-64	25-64	454	794	
723	Saint Lucia	2012	STEPS	National	both	25-64	25-64	573	918	
724	Samoa	1995	McGarvey ST. Cardiovascular disease (CVD) risk factors in Samoa and American Samoa, 1990-95. Pac Health Dialog 2001; 8: 157-62	National	both	25+	25+	144	151	
725	Samoa	2002	STEPS	National	both	25-64	25-64	1,197	1,346	
726	Samoa	2010	Samoan Genome-Wide Association Study	National	both	24-65	24-65	1,396	2,043	
727	Samoa	2013	STEPS	National	both	18-64	18-64	604	901	
728	Sao Tome and Principe	2009	STEPS	National	both	25-64	25-64	1,047	1,322	
729	Saudi Arabia	2007	Gulf Cooperation Council World Health Survey	National	both	18+	18+	4,906	3,701	
730	Saudi Arabia	2011-2013	Jeddah City Study	Community	urban	10+	10+	817	823	
731	Saudi Arabia	2013	Saudi Health Information Survey	National	both	15+	15+	5,194	5,411	
732	Serbia	1988-1989	MONICA, Novi Sad	Community	urban	25-64	25-64	778	791	
733	Serbia	1994-1995	MONICA, Novi Sad	Community	urban	25-64	25-64	600	670	
734	Serbia	2000	Health Status, Health Needs and Utilization of Health Care of the Population of Serbia	National	both	10+	10+	4,812	5,915	
735	Serbia	2006	The 2006 National Health Survey for the Population of Serbia	National	both	10+	10+	7,683	8,558	
736	Serbia	2013	The National Health Survey 2013	National	both	15+	15+	6,586	7,630	
737	Serbia	2013-2014	Stay Fit for Lifelong Health; The Prevalence of Lifestyle Health Conditions in Serbian Population	National	urban	18-65		1,367		
738	Seychelles	1989	Seychelles Heart Survey I	National	both	25-64	25-64	513	568	
739	Seychelles	1994	Seychelles Heart Survey II	National	both	25-64	25-64	504	563	
740	Seychelles	2004	Seychelles Heart Survey III	National	both	25-64	25-64	568	687	
741	Seychelles	2013-2014	Seychelles Heart Survey IV	National	both	25-64	25-64	531	699	
742	Sierra Leone	2009	STEPS	National	both	25-64	25-64	2,214	2,332	

	Country	Data years	Survey/Study name/Citation	Level of representative-ness	Rural, urban or both	Age range as in the NCD-RisC database		Sample size		Note
						Male	Female	Male	Female	
743	Singapore	1993-1995	NUH Heart Study	National	both	26+	26+	498	484	
744	Singapore	2004-2007	Combined follow up of Singapore Cardiovascular Cohort study and Singapore Prospective study	National	both	24+	24+	2,467	2,666	
745	Singapore	2009	Social Isolation, Health and Lifestyles Survey (SIHLS) 2009	National	both	60+	60+	2,050	2,438	
746	Singapore	2009-2011	The Singapore Chinese Eye Study	Community	both	40+	40+	1,662	1,691	
747	Singapore	2012-2013	Singapore Health Study 2012	National	both	18-79	18-79	958	1,026	
748	Slovakia	1993	Countrywide Integrated Noncommunicable Diseases Intervention Programme	National	both	15-64	15-64	877	1,290	
749	Slovakia	1998	Countrywide Integrated Noncommunicable Diseases Intervention Programme	National	both	15-64	15-64	923	1,122	
750	Slovakia	2003	Countrywide Integrated Noncommunicable Diseases Intervention Programme	National	both	15-64	15-64	664	904	
751	Slovakia	2008	Countrywide Integrated Noncommunicable Diseases Intervention Programme	National	both	15-64	15-64	412	584	
752	Slovakia	2011-2012	European Health Examination Survey	National	both	18-64	18-64	885	1,080	
753	Solomon Islands	2006	STEPS	Subnational	both	15-64	15-64	1,032	1,376	
754	Solomon Islands	2009-2010	Influences of the large-scale disaster and related socioecological changes on people's nutritional status and health in the Western Solomon Islands	Subnational	rural	18+	18+	153	215	
755	Solomon Islands	2015	STEPS	National	both	18-69	18-69	841	1,010	
756	South Africa	1998	Demographic and Health Survey South Africa 1998	National	both	15+	15+	5,659	7,704	
757	South Africa	2003	Demographic and Health Survey South Africa 2003	National	both	15+	15+	3,265	4,566	
758	South Africa	2003-2004	Africa Centre Biomeasure Survey	Community	rural	25-49	25-49	790	1,758	
759	South Africa	2007-2008	SAGE	National	both	50+	50+	1,584	2,138	
760	South Africa	2008	National Income Dynamics Study Wave I	National	both	15+	15+	5,562	8,198	
761	South Africa	2008-2009	Cape Town Bellville South Cohort Study - Baseline evaluation I	Community	urban	16+	16+	220	710	
762	South Africa	2010	Africa Centre Biomeasure Survey	Community	rural	15+	15+	3,466	7,819	
763	South Africa	2010-2011	National Income Dynamics Study Wave II	National	both	15+	15+	6,208	8,760	
764	South Africa	2012	National Income Dynamics Study Wave III	National	both	15+	15+	7,428	10,610	
765	South Africa	2014-2015	Health and Aging in Africa: A Longitudinal Study of an INDEPTH Community in South Africa (HAALSI)	Community	rural	40+	40+	2,243	2,603	
766	South Korea	1986	INTERSALT	Community	urban	20-59	20-59	100	98	
767	South Korea	1998	Korea National Health and Nutrition Examination Survey	National	both	10+	10+	4,514	5,193	
768	South Korea	2001	Korea National Health and Nutrition Examination Survey	National	both	10+	10+	3,269	3,998	
769	South Korea	2002-2003	Korean National Health Insurance	National	both	40+	40+	3,002,129	2,506,319	
770	South Korea	2004-2005	Korean National Health Insurance	National	both	40+	40+	3,613,977	3,288,304	
771	South Korea	2005	Korea National Health and Nutrition Examination Survey	National	both	10+	10+	2,863	3,589	
772	South Korea	2006-2007	Korean National Health Insurance	National	both	40+	40+	4,358,877	4,402,085	
773	South Korea	2007	Korea National Health and Nutrition Examination Survey	National	both	10+	10+	1,547	1,998	
774	South Korea	2008	Korea National Health and Nutrition Examination Survey	National	both	10+	10+	3,480	4,478	
775	South Korea	2008-2009	Korean National Health Insurance	National	both	40+	40+	5,521,545	5,785,937	
776	South Korea	2009	Korea National Health and Nutrition Examination Survey	National	both	10+	10+	3,915	4,839	
777	South Korea	2010	Korea National Health and Nutrition Examination Survey	National	both	10+	10+	3,264	4,021	
778	South Korea	2010-2011	Korean National Health Insurance	National	both	40+	40+	6,688,661	7,149,463	
779	South Korea	2011	Korea National Health and Nutrition Examination Survey	National	both	10+	10+	3,104	3,941	
780	South Korea	2012	Korea National Health and Nutrition Examination Survey	National	both	10+	10+	2,924	3,826	
781	South Korea	2012-2013	Korean National Health Insurance	National	both	40+	40+	7,270,222	7,800,669	
782	South Korea	2013	Korea National Health and Nutrition Examination Survey	National	both	10+	10+	2,934	3,686	
783	South Korea	2014	Korea National Health and Nutrition Examination Survey	National	both	10+	10+	2,727	3,551	
784	South Korea	2014-2015	Korean National Health Insurance	National	both	40+	40+	7,881,544	8,371,302	
785	South Korea	2015	Korea National Health and Nutrition Examination Survey	National	both	10+	10+	2,816	3,432	
786	Spain	1985	INTERSALT, Manresa	Community	urban	20-59	20-59	100	100	
787	Spain	1986	INTERSALT, Torrejo	Community	urban	20-59	20-59	100	100	
788	Spain		Plans et al., Med Clin (Barc) 1992; 98: 369-72	Community	urban	15+	15+			
789	Spain	1986-1988	MONICA, Catalonia	Community	urban	25-64	25-64	1,252	1,271	
790	Spain	1989	Cardiovascular Risk Factors Study in Catalonia	Subnational	both	15-85	15-85	330	372	
791	Spain	1990	Blood pressure in Spain: distribution, awareness, control, and benefits of a reduction in average pressure. Hypertension 1998; 32: 998-1002	National	both	35-65	35-65	810	1,211	

	Country	Data years	Survey/Study name/Citation	Level of representative-ness	Rural, urban or both	Age range as in the NCD-RisC database		Sample size		Note
						Male	Female	Male	Female	
792	Spain	1990-1992	MONICA, Catalonia	Community	urban	25-64	25-64	1,720	1,192	
793	Spain	1991-1993	Encuesta de Factores de Riesgo Cardiovascular en la Región de Murcia (Cardiovascular Risk Factors Survey)	Subnational	both	18-69	18-69	1,512	1,573	
794	Spain	1994-1995	Encuesta de Nutrición y Salud Comunidad Valenciana 1994-95 (ENCV)	Subnational	urban	15+	15+	813	957	
795	Spain	1994-1996	MONICA, Catalonia	Community	urban	25-64	25-64	1,802	1,631	
796	Spain	1999-2000	Factores de riesgo en las islas Baleares: Estudio CORSAIB	Subnational	both	35-74	35-74	801	856	
797	Spain	2000-2001	EUREYE Study	Subnational	both	65+	65+	274	324	
798	Spain	2000-2001	Influence of childhood socioeconomic circumstances, height, and obesity on pulse pressure and systolic and diastolic blood pressure in older people. J Hum Hypertens 2006; 20: 73-82	National	both	60+	60+	1,408	2,461	
799	Spain	2001-2002	Catalan Health Interview Survey	Subnational	both	18-74	18-74	606	760	
800	Spain	2001-2003	DIabetes, Nutrición y Obesidad en la población adulta de la Región de Murcia (DINO)	Subnational	both	20+	20+	717	834	
801	Spain	2000-2003	CDC of the Canary Islands	Subnational	both	18-75	18-75	2,889	4,033	
802	Spain	2003	The European Male Ageing Study	Community	both	40+		406		
803	Spain	2003-2005	Registre Gironi del Cor (REGICOR)	Subnational	both	35-79	35-79	2,964	3,289	
804	Spain	2004	Cardiovascular Risk Study in Castilla y León (RECCyL)	Subnational	both	15+	15+	1,913	2,073	
805	Spain	2004-2006	PREVICTUS	National	both	60+	60+	3,416	3,915	
806	Spain	2007-2009	Harmonizing Equation of Risk in Mediterraneon countries EXtremadura	Subnational	both	25-79	25-79	1,298	1,498	
807	Spain	2008	The European Male Ageing Study	Community	both	40+		267		
808	Spain	2008-2010	Study on Nutrition and Cardiovascular Risk in Spain	National	both	18+	18+	5,792	6,414	
809	Spain	2009	Cardiovascular Risk Study in Castilla y León (RECCyL)	Subnational	both	20+	20+	1,322	1,601	
810	Spain	2014	Cardiovascular Risk Study in Castilla y León (RECCyL)	Subnational	both	20+	20+	1,228	1,500	
811	Spain	2015	National Study of Nutrition and Cardiovascular Risk (ENRICA)	National	both	60+	60+	741	799	
812	Sri Lanka	2006	STEPS	National	both	15-64	15-64	6,153	6,222	
813	Sri Lanka	2014	STEPS	National	both	18-69	18-69	1,909	2,969	
814	Suriname	2013-2015	The Healthy Life in Suriname Study (HELISUR)	Subnational	urban	18-70	18-70	424	722	
815	Swaziland	2014	STEPS	National	both	15-69	15-69	1,113	1,988	
816	Sweden	1985	MONICA Gothenburg	Community	urban	25-64	25-64	550	595	
817	Sweden	1986	MONICA, Northern Sweden	Subnational	urban	25-64	25-64	820	799	
818	Sweden	1990	MONICA Gothenburg	Community	urban	25-64	25-64	766	766	
819	Sweden	1990	MONICA, Northern Sweden	Subnational	urban	25-64	25-64	764	794	
820	Sweden	1985-1996	EPIC Umea	Subnational	both	24-72	24-72	12,139	12,985	
821	Sweden	1991-1996	Malmö Diet and Cancer	Community	urban	45-73	45-73	12,101	18,291	
822	Sweden	1994	MONICA, Northern Sweden	Subnational	urban	25-64	25-64	736	769	
823	Sweden	1995	MONICA Gothenburg	Community	urban	25-64	25-64	741	863	
824	Sweden	1999	MONICA Northern Sweden	Subnational	both	25-74	25-74	889	934	
825	Sweden	2001-2004	Swedish INTERGENE Cohort Study	Subnational	both	24-76	24-76	1,691	1,905	
826	Sweden	2003	The European Male Ageing Study	Community	both	40+		397		
827	Sweden	2004	MONICA Northern Sweden	Subnational	both	25-74	25-74	910	957	
828	Sweden	2008	The European Male Ageing Study	Community	both	40+		370		
829	Sweden	2009	MONICA Northern Sweden	Subnational	both	25-74	25-74	850	872	
830	Sweden	2014	MONICA Northern Sweden	Subnational	both	25-74	25-74	756	806	
831	Switzerland	1984-1986	The Swiss MONICA Study Wave I	Subnational	both	25-74	25-74	1,749	1,692	
832	Switzerland	1988-1989	The Swiss MONICA Study Wave II	Subnational	both	25-74	25-74	1,775	1,683	
833	Switzerland	1992-1993	The Swiss MONICA Study Wave III	Subnational	both	25-74	25-74	1,570	1,670	
834	Switzerland	2003-2006	Cohorte Lausannoise	Community	urban	35-75	35-75	3,185	3,531	
835	Switzerland	2007-2012	Bus Santé Study	Subnational	both	20-80	20-80	1,886	1,913	
836	Switzerland	2009-2012	Cohorte Lausannoise	Community	urban	40-81	40-81	2,199	2,507	
837	Taiwan	1985	INTERSALT	Community	rural	20-59	20-59	89	92	
838	Taiwan	1993-1994	The Kinmen Neurological Disorders Survey	Community	urban	50+	50+	1,446	1,338	
839	Taiwan	1993-1996	Nutrition and Health Survey in Taiwan 1993-1996	National	both	10+	10+	3,988	3,990	
840	Taiwan	1999-2000	Nutrition and Health Survey in Taiwan 1999-2000	National	both	65+	65+	1,277	1,217	
841	Taiwan	2002	Taiwanese Survey on Hypertension, Hyperglycemia and Hyperlipidemia	National	both	15+	15+	3,335	3,606	

	Country	Data years	Survey/Study name/Citation	Level of representative-ness	Rural, urban or both	Age range as in the NCD-RisC database		Sample size		Note
						Male	Female	Male	Female	
842	Taiwan	2005-2008	Nutrition and Health Survey in Taiwan 2005-2008	National	both	19+	19+	1,342	1,402	
843	Taiwan	2007	Taiwanese Survey on Hypertension, Hyperglycemia and Hyperlipidemia	National	both	20+	20+	2,160	2,468	
844	Taiwan	2013-2016	Nutrition And Health Survey in Taiwan	National	both	10+	10+	2,152	2,221	
845	Tajikistan	2016	STEPS	National	both	18-69	18-69	1,092	1,554	
846	Tanzania	1998-1999	Bovet et al., Int J Epidemiol 2002; 31: 240-7	Community	urban	25-64	25-64	3,600	5,653	
847	Tanzania	2011	STEPS	Subnational	both	25-64	25-64	1,007	1,504	
848	Tanzania	2012	STEPS	National	both	25-64	25-64	2,585	2,834	
849	Tanzania	2014	Dar es Salaam Urban Cohort Hypertension Study	Community	urban	40+	40+	971	1,271	
850	Thailand	1991	Thailand National Health Examination Survey I	National	both	15+	15+	6,238	8,689	
851	Thailand	1997	Thailand National Health Examination Survey II	National	both	10+	10+	4,195	5,680	
852	Thailand	2000	InterASIA	National	both	35+	35+	2,093	3,212	
853	Thailand	2004	Thailand National Health Examination Survey III	National	both	15+	15+	18,900	20,244	
854	Thailand	2009	Thailand National Health Examination Survey IV	National	both	15+	15+	9,718	10,615	
855	Timor-Leste	2014	STEPS	National	both	18-69	18-69	1,066	1,452	
856	Togo	2010	STEPS	National	both	15-64	15-64	2,023	2,026	
857	Tokelau	2005	STEPS	National	both	15-64	15-64	270	295	
858	Tokelau	2014	STEPS	National	both	18-64	18-64	256	279	
859	Tonga	2004	STEPS	National	both	15-64	15-64	404	554	
860	Tonga	2011	STEPS	National	both	15-64	15-64	884	1,412	
861	Trinidad and Tobago	1985	INTERSALT	Community	urban	20-59	20-59	84	92	
862	Trinidad and Tobago	2001	Adult Survey	National	rural	25+	25+	204	267	
863	Tunisia	1996-1997	Ariana Healthy Project 1997	Community	both	35-65	35-65	2,655	2,724	
864	Tunisia	1996-1997	Tunisian National Nutrition Survey 1996-1997	National	both	10+	10+	2,164	3,549	
865	Tunisia	2005	Tunisian National Survey	National	both	35-70	35-70	3,417	4,590	
866	Tunisia	2009-2010	ObeMaghreb	Subnational	urban	10-49	10-49	1,515	1,273	
867	Turkey	1990	Turkish Adult Risk Factor Study	National	both	20+	20+	1,344	1,372	
868	Turkey	1995	Turkish Adult Risk Factor Study	National	both	25+	25+	864	894	
869	Turkey	1998	Turkish Adult Risk Factor Study	National	both	28+	28+	883	915	
870	Turkey	2000	Turkish Adult Risk Factor Study	National	both	30+	30+	905	952	
871	Turkey	2001-2002	Turkish Adult Risk Factor Study	National	both	32+	32+	1,137	1,252	
872	Turkey	2003	Prevalence, awareness, treatment and control of hypertension in Turkey in 2003	National	both	18+	18+	2,019	2,891	
873	Turkey	2003-2004	Turkish Adult Risk Factor Study	National	both	34+	34+	1,106	1,150	
874	Turkey	2003-2005	Prevalence of diabetes and associated risk factors among adult population in Trabzon city	Subnational	both	20+	20+	2,208	2,601	
875	Turkey	2005-2006	Turkish Adult Risk Factor Study	National	both	33+	33+	1,031	1,091	
876	Turkey	2007-2008	Turkish Adult Risk Factor Study	National	both	35+	35+	1,134	1,148	
877	Turkey	2009-2010	Turkish Adult Risk Factor Study	National	both	37+	37+	1,490	1,555	
878	Turkey	2011	Chronic Diseases and Risk Factors Survey in Turkey	National	both	15+	15+	7,995	8,906	
879	Turkey	2012-2013	Turkish Adult Risk Factor Study	National	both	37+	37+	1,030	1,110	
880	Turkey	2014-2015	Turkish Adult Risk Factor Study	National	both	44+	44+	746	768	
881	Turkmenistan	2013	STEPS	National	both	18-64	18-64	1,931	2,886	
882	Uganda	2011-2012	The Prevalence and Distribution of Non-communicable Diseases and Their Risk Factors in Kasese District, Uganda	Subnational	both	25-79	25-79	284	234	
883	Uganda	2014	STEPS	National	both	18-69	18-69	1,570	2,132	
884	Ukraine	2007	Demographic and Health Survey Ukraine 2007	National	both	15-49	15-49	2,555	5,537	
885	United Arab Emirates	2009	Gulf Cooperation Council World Health Survey	National	both	18+	18+	624	679	
886	United Kingdom	1984-1986	Scottish Heart Health Survey	Subnational	both	40-59	40-59	4,385	4,471	
887	United Kingdom	1985	INTERSALT, Birmingham	Community	urban	20-59	20-59	100	100	
888	United Kingdom	1985	INTERSALT, Wales	Community	urban	20-59	20-59	100	99	
889	United Kingdom	1985-1986	INTERSALT, Belfast	Community	urban	20-59	20-59	99	100	
890	United Kingdom	1986-1987	MONICA, Belfast	Subnational	both	25-64	25-64	1,164	1,185	
891	United Kingdom	1986-1987	Dietary and Nutritional Survey of British Adults 1986-1987	National	both	16-64	16-64	1,190	1,182	

	Country	Data years	Survey/Study name/Citation	Level of representative-ness	Rural, urban or both	Age range as in the NCD-RisC database		Sample size		Note
						Male	Female	Male	Female	
892	United Kingdom	1987-1988	Edinburgh Artery Study	Community	urban	54-75	54-75	808	783	
893	United Kingdom	1991-1992	MONICA, Belfast	Subnational	both	25-64	25-64	999	997	
894	United Kingdom	1991-1992	Health Survey for England	National	both	16+	16+	2,951	3,319	
895	United Kingdom	1992	MONICA, Glasgow	Community	urban	25-64	25-64	704	779	
896	United Kingdom	1992-1994	Edinburgh Artery Study	Community	urban	60-81	60-81	580	580	
897	United Kingdom	1993	Health Survey for England	National	both	16+	16+	6,745	7,489	
898	United Kingdom	1994	Health Survey for England	National	both	16+	16+	6,223	7,178	
899	United Kingdom	1993-1997	EPIC Norfolk	Subnational	both	40-79	40-79	11,551	13,961	
900	United Kingdom	1994-1995	National Diet and Nutrition Survey (NDNS)	National	both	65+	65+	743	730	
901	United Kingdom	1995	MONICA, Glasgow	Community	urban	25-64	25-64	857	958	
902	United Kingdom	1995	Scottish Health Survey (SHeS)	Subnational	both	16-64	16-64	3,104	3,747	
903	United Kingdom	1995	Health Survey for England	National	both	10+	10+	6,977	7,954	
904	United Kingdom	1996	Health Survey for England	National	both	10+	10+	7,280	8,309	
905	United Kingdom	1993-2000	EPIC Oxford	Subnational	both	20-98	20-98	2,444	8,923	
906	United Kingdom	1997	Health Survey for England	National	both	10+	10+	4,771	5,261	
907	United Kingdom	1997-1999	INTERMAP, WestBromwich	Community	urban	40-59	40-59	141	138	
908	United Kingdom	1998	Health Survey for England	National	both	10+	10+	6,767	7,828	
909	United Kingdom	1998	Scottish Health Survey (SHeS)	Subnational	both	10-74	10-74	3,958	4,781	
910	United Kingdom	1998-1999	INTERMAP, Belfast	Community	urban	40-59	40-59	125	97	
911	United Kingdom	1998-2000	The British Regional Heart Study	National	urban	60-79		4,141		
912	United Kingdom	1999-2001	Edinburgh Artery Study	Community	urban	66-87	66-87	373	404	
913	United Kingdom	1999-2001	British Women's Heart and Health Study	National	both		60-79		3,801	
914	United Kingdom	2000	Health Survey for England	National	both	16+	16+	3,301	4,197	
915	United Kingdom	1999-2004	Hertfordshire Cohort Study	Subnational	both	59-73	59-73	1,574	1,411	
916	United Kingdom	2000-2001	National Diet and Nutrition Survey 2000-2001	National	both	19-64	19-64	797	939	
917	United Kingdom	2001	Health Survey for England	National	both	10+	10+	6,156	7,191	
918	United Kingdom	2002	Health Survey for England	National	both	10+	10+	4,818	5,725	
919	United Kingdom	2003	Scottish Health Survey (SHeS)	Subnational	both	10+	10+	2,790	3,407	
920	United Kingdom	2003	Health Survey for England	National	both	10+	10+	5,566	6,683	
921	United Kingdom	2003	The European Male Ageing Study	Community	both	40+		396		
922	United Kingdom	2004-2005	English Longitudinal Study of Ageing Wave 2 2004-2005	National	both	52+	52+	3,404	4,159	
923	United Kingdom	2005	Health Survey for England	National	both	10+	10+	3,518	4,239	
924	United Kingdom	2006	Health Survey for England	National	both	10+	10+	5,123	6,088	
925	United Kingdom	2007	Health Survey for England	National	both	10+	10+	2,427	2,879	
926	United Kingdom	2008	Health Survey for England	National	both	10+	10+	5,170	6,194	
927	United Kingdom	2008	The European Male Ageing Study	Community	both	40+		315		
928	United Kingdom	2008	Scottish Health Survey (SHeS)	Subnational	both	16+	16+	492	601	
929	United Kingdom	2008-2009	English Longitudinal Study of Ageing Wave 4 2008-2009	National	both	50+	50+	3,833	4,630	
930	United Kingdom	2009	Scottish Health Survey (SHeS)	Subnational	both	16+	16+	472	623	
931	United Kingdom	2009	Health Survey for England	National	both	10+	10+	1,450	1,733	
932	United Kingdom	2008-2012	National Diet and Nutrition Survey (NDNS)	National	both	10+	10+	1,037	1,241	
933	United Kingdom	2010	Health Survey for England	National	both	10+	10+	2,611	3,283	
934	United Kingdom	2010	Scottish Health Survey (SHeS)	Subnational	both	16+	16+	443	584	
935	United Kingdom	2011	Scottish Health Survey (SHeS)	Subnational	both	16+	16+	421	536	
936	United Kingdom	2011	Health Survey for England	National	both	10+	10+	2,648	3,313	
937	United Kingdom	2012	Scottish Health Survey (SHeS)	Subnational	both	16+	16+	430	535	
938	United Kingdom	2012	Health Survey for England	National	both	10+	10+	2,548	3,196	
939	United Kingdom	2012-2013	English Longitudinal Study of Ageing Wave 6 2012-2013	National	both	50+	50+	3,528	4,284	
940	United Kingdom	2013	Scottish Health Survey (SHeS)	Subnational	both	16+	16+	529	654	
941	United Kingdom	2013	Health Survey for England	National	both	10+	10+	2,932	3,632	
942	United Kingdom	2013-2014	National Diet and Nutrition Survey (NDNS)	National	both	10+	10+	623	844	

	Country	Data years	Survey/Study name/Citation	Level of representative-ness	Rural, urban or both	Age range as in the NCD-RisC database		Sample size		Note
						Male	Female	Male	Female	
943	United Kingdom	2014	Scottish Health Survey (SHeS)	Subnational	both	16+	16+	550	712	
944	United Kingdom	2014	Health Survey for England	National	both	10+	10+	2,657	3,168	
945	United Kingdom	2015	Scottish Health Survey (SHeS)	Subnational	both	16+	16+	385	492	
946	United States of Americ	1983-1987	Framingham Heart Study (FHS) - Offspring Cohort	Community	urban	24-72	24-72	1,819	1,965	
947	United States of Americ	1985-1986	MONICA, Stanford	Subnational	urban	25-64	25-64	703	824	5
948	United States of Americ	1985-1986	Coronary Artery Risk Development in Young Adults (CARDIA)	Subnational	urban	18-30	18-30	2,327	2,785	
949	United States of Americ	1985-1986	INTERSALT, Chicago	Community	urban	20-59	20-59	97	99	
950	United States of Americ	1986	INTERSALT, Goodman	Community	urban	20-59	20-59	192	192	
951	United States of Americ	1986	INTERSALT, Jackson	Community	urban	20-59	20-59	184	199	
952	United States of Americ	1985-1988	Framingham Heart Study (FHS)	Community	urban	65-93	65-96	559	881	
953	United States of Americ	1986-1990	Framingham Heart Study (FHS)	Community	urban	68-95	67-97	499	802	
954	United States of Americ	1987-1988	Coronary Artery Risk Development in Young Adults (CARDIA)	Subnational	urban	20-32	20-32	2,089	2,531	
955	United States of Americ	1987-1989	Atherosclerosis Risk in Communities Study (ARIC)	Subnational	both	44-66	44-66	8,153	6,784	
956	United States of Americ	1987-1991	Framingham Heart Study (FHS) - Offspring Cohort	Community	urban	28-76	28-76	1,886	2,039	
957	United States of Americ	1988-1992	Framingham Heart Study (FHS)	Community	urban	69-97	69-99	430	780	
958	United States of Americ	1989-1990	MONICA, Stanford	Subnational	urban	25-64	25-64	655	754	5
959	United States of Americ	1988-1994	US NHANES III	National	both	17+	17+	8,966	9,843	
960	United States of Americ	1990-1992	Atherosclerosis Risk in Communities Study (ARIC)	Subnational	both	46-70	47-70	7,415	6,120	
961	United States of Americ	1991-1995	Framingham Heart Study (FHS) - Offspring Cohort	Community	urban	31-78	31-78	1,742	1,969	
962	United States of Americ	1993-1995	Atherosclerosis Risk in Communities Study (ARIC)	Subnational	both	50-73	49-73	6,587	5,529	
963	United States of Americ	1993-1998	Women's Health Initiative Study (WHI OS)	National	both		50-79		93,545	
964	United States of Americ	1995-1996	Coronary Artery Risk Development in Young Adults (CARDIA)	Subnational	urban	28-40	28-40	1,754	2,182	
965	United States of Americ	1995-1996	Bogalusa Heart Study (BHS)	Community	rural	20-37	20-37	549	843	
966	United States of Americ	1995-1998	Framingham Heart Study (FHS) - Offspring Cohort	Community	urban	37-80	37-80	1,603	1,829	
967	United States of Americ	1996-1997	INTERMAP, Jackson	Community	urban	40-59	40-59	132	134	
968	United States of Americ	1996-1997	INTERMAP, Pittsburgh	Community	urban	40-59	40-59	132	128	
969	United States of Americ	1996-1997	Study of Women's Health Across the Nation	Subnational	both		40-55		3,233	6
970	United States of Americ	1996-1997	INTERMAP, Baltimore	Community	urban	40-59	40-59	146	134	
971	United States of Americ	1996-1998	Atherosclerosis Risk in Communities Study (ARIC)	Subnational	both	53-75	52-75	6,020	4,880	
972	United States of Americ	1996-1998	INTERMAP, Minneapolis	Community	urban	40-59	40-59	130	130	
973	United States of Americ	1997-1998	INTERMAP, CC	Community	urban	40-59	40-59	271	276	
974	United States of Americ	1997-1998	INTERMAP, Chicago	Community	urban	40-59	40-59	156	159	
975	United States of Americ	1997-1999	Study of Women's Health Across the Nation	Subnational	both		40-55		2,840	6
976	United States of Americ	1998-2000	Study of Women's Health Across the Nation	Subnational	both		40-55		2,661	6
977	United States of Americ	1998-2001	Framingham Heart Study (FHS) - Offspring Cohort	Community	urban	37-89	33-90	1,601	1,887	
978	United States of Americ	1999-2000	US NHANES 1999-2000	National	both	10+	10+	3,316	3,276	
979	United States of Americ	1999-2001	Study of Women's Health Across the Nation	Subnational	both		40-56		2,526	6
980	United States of Americ	2000-2002	Multi-Ethnic Study of Atherosclerosis (MESA)	Subnational	both	45-84	45-84	3,210	3,596	
981	United States of Americ	2000-2002	Study of Women's Health Across the Nation	Subnational	both		40-57		2,457	6
982	United States of Americ	2001-2002	US NHANES 2001-2002	National	both	10+	10+	3,587	3,489	
983	United States of Americ	2002-2004	Multi-Ethnic Study of Atherosclerosis (MESA)	Subnational	both	46-87	46-86	2,969	3,261	
984	United States of Americ	2002-2005	Framingham Heart Study (FHS) - Third Generation Cohort	Community	urban	19-72	19-70	1,904	2,174	
985	United States of Americ	2003-2004	US NHANES 2003-2004	National	both	10+	10+	3,376	3,215	
986	United States of Americ	2004-2005	Multi-Ethnic Study of Atherosclerosis (MESA)	Subnational	both	47-88	47-87	2,809	3,128	
987	United States of Americ	2005-2006	US NHANES 2005-2006	National	both	10+	10+	3,382	3,230	
988	United States of Americ	2005-2006	National Social Life Health and Aging Project	National	both	57-85	57-85	1,423	1,511	7
989	United States of Americ	2005-2007	Multi-Ethnic Study of Atherosclerosis (MESA)	Subnational	both	49-90	49-89	2,690	3,008	
990	United States of Americ	2005-2008	Framingham Heart Study (FHS) - Offspring Cohort	Community	urban	43-92	40-93	1,349	1,648	
991	United States of Americ	2007-2008	US NHANES 2007-2008	National	both	10+	10+	3,468	3,432	
992	United States of Americ	2009-2010	US NHANES 2009-2010	National	both	10+	10+	3,677	3,687	
993	United States of Americ	2010-2011	National Social Life Health and Aging Project	National	both	36-99	36-99	1,484	1,791	7

	Country	Data years	Survey/Study name/Citation	Level of representative-ness	Rural, urban or both	Age range as in the NCD-RisC database		Sample size		Note
						Male	Female	Male	Female	
994	United States of Americ	2011-2012	US NHANES 2011-2012	National	both	10+	10+	3,318	3,269	
995	United States of Americ	2013-2014	US NHANES 2013-2014	National	both	10+	10+	3,447	3,624	
996	Uruguay	2006	STEPS	National	both	25-64	25-64	261	644	
997	Uruguay	2011-2012	Detection and follow-up of cardiovascular disease and risk factors in the Southern Cone of Latin America. The CESCAS I Study	Community	urban	35-74	35-74	652	932	
998	Uruguay	2012-2016	Genotype, Phenotype and Environment of Hypertension in Uruguay (GEFA-HT-UY)	Community	urban	19+	19+	128	190	
999	Uzbekistan	2002	Demographic and Health Survey Uzbekistan 2002	National	both	15-59	15-49	2,331	5,225	
1000	Uzbekistan	2014	STEPS	National	both	18-64	18-64	1,539	2,170	
1001	Vanuatu	2005	STEPS	Subnational	both	15-60	15-60	631	763	
1002	Vanuatu	2011	STEPS	National	both	25-64	25-64	2,283	2,205	
1003	Venezuela	2005-2006	Brajkovich et al. Prevalencia de sobrepeso y obesidad en una poblacion de Catia La Mar (Edo.Vargas) y Municipio Sucre Distrito Metropolitano de Caracas, Rev Ven Endoc Metab 2006; 4 (3): 31-32	Community	urban	20-65	20-65	200	432	
1004	Venezuela	2007-2008	Venezuelan Study of Metabolic Syndrome, Obesity and Lifestyle (VEMSOLS)	Community	urban	20+	20+	107	232	
1005	Venezuela	2008-2009	Venezuelan Study of Metabolic Syndrome, Obesity and Lifestyle (VEMSOLS)	Community	rural	20+	20+	51	87	
1006	Venezuela	2010-2011	Venezuelan Study of Metabolic Syndrome, Obesity and Lifestyle (VEMSOLS)	Community	urban	20+	20+	66	192	
1007	Venezuela	2015-2017	Cardio-Metabolic Health Venezuelan Study (EVESCAM)	National	both	20+	20+	1,062	2,351	
1008	Viet Nam	2001-2002	Viet Nam National Health Survey 2001-2002	National	both	16+	16+	46,381	52,804	
1009	Viet Nam	2005	National Adult Overweight Survey	National	both	25-64	25-64	8,474	8,725	
1010	Viet Nam	2005	Non-communicable disease risk factors in Ho Chi Minh City	Community	urban	25-64	25-64	906	1,058	
1011	Viet Nam	2008-2009	The survey on diabetes and its risk factors in 2 northern provinces of Vietnam (DM-S)	Subnational	both	25+	25+	830	1,446	
1012	Viet Nam	2009	STEPS	National	both	25-64	25-64	6,740	7,806	
1013	Viet Nam	2012	National Survey of Diabetes in Vietnam	National	both	30-69	30-69	5,319	5,855	
1014	Viet Nam	2015	STEPS	National	both	18-69	18-69	1,319	1,723	
1015	Yemen	2007-2009	Hypertension and Diabetes in Yemen (HYDY)	National	urban	10-70	10-70	2,840	2,927	
1016	Yemen	2007-2009	Hypertension and Diabetes in Yemen (HYDY)	National	rural	10-70	10-70	2,842	2,875	
1017	Zambia	2008	STEPS	Community	urban	25+	25+	632	1,220	
1018	Zimbabwe	1985-1986	INTERSALT	Community	urban	20-59	20-59	100	95	

1. This research uses data from China Health and Nutrition Survey (CHNS). We thank the National Institute of Nutrition and Food Safety, China Center for Disease Control and Prevention, Carolina Population Center (5 R24 HD050924), the University of North Carolina at Chapel Hill, the NIH (R01-HD30880, DK056350, R24HD050924, and R01-HD38700) and the Fogarty International Center, NIH for financial support for the CHNS data collection and analysis files from 1989 to 2011 and future surveys, and the China-Japan Friendship Hospital, Ministry of Health for support for CHNS 2009.

2. The bibliographic citation for this data source is: Pelaez, Martha, Alberto Palloni, Cecilia Albala, Juan C. Alfonso, Roberto Ham-Chande, Anselm Hennis, Maria Lucia Lebrao, Esther Lesn-Diaz, Edith Pantelides, and Omar Prats. SABE - SURVEY ON HEALTH, WELL-BEING, AND AGING IN LATIN AMERICA AND THE CARIBBEAN, 2000 [Computer file]. ICPSR version. Washington, D.C.: Pan American Health Organization/World Health Organization (PAHO/WHO) [producers], 2004. Ann Arbor, MI: Inter-university Consortium for Political and Social Research [distributor], 2005.

3. The Golestan Cohort Study was funded by Tehran University of Medical Sciences number 81/15.

4. The Longitudinal Aging Study Amsterdam is supported by a grant from the Netherlands Ministry of Health Welfare and Sports, Directorate of Long-Term Care.

5. We thank Prof Stephen Fortmann for data from the Stanford Five-City Project.

6. The bibliographic citation for this data source is: Sutton-Tyrrell, Kim, Faith Selzer, MaryFran Sowers, Robert Neer, Lynda Powell, Ellen Gold, Gail Greendale, Gerson Weiss, Karen Matthews, and Sonja McKinlay. Study of Women's Health Across the Nation (SWAN), 1996-1997: Baseline Dataset. ICPSR28762-v2. Ann Arbor, MI: Inter-university Consortium for Political and Social Research[distributor], 2014-02-04. http://doi.org/10.3886/ICPSR28762.v2

7. The bibliographic citation for this data source is: Waite, Linda J., Kathleen Cagney, William Dale, Elbert Huang, Edward O. Laumann, Martha McClintock, Colm A. O'Muircheartaigh, L. Phillip Schumm, and Benjamin Cornwell. National Social Life, Health, and Aging Project (NSHAP): Wave 2 and Partner Data Collection. ICPSR34921-v1. Ann Arbor, MI: Inter-university Consortium for Political and Social Research [distributor], 2014-04-29. https://doi.org/10.3886/ICPSR34921.v1

Supplementary Table 5. Coefficients of the regression of the probit-transformed prevalence of raised blood pressure on mean systolic and diastolic blood pressure for women.

**Variable**	**Coefficient (95% CI)**
Intercept	-0.48 (-0.51, -0.44)
Mean SBP (mm Hg)	0.028 (0.022, 0.034)
Mean DBP (mm Hg)	0.035 (0.026, 0.044)
Age group (years)	
20-29	-0.39 (-0.54, -0.25)
30-39	-0.22 (-0.31, -0.13)
40-49	-0.093 (-0.15, -0.041)
50-59	Reference
60-69	0.088 (0.040, 0.14)
70-79	0.14 (0.068, 0.20)
Decade	
2005-2015	Reference
1995-2004	0.052 (0.012, 0.092)
1985-1994	0.077 (0.028, 0.12)
Mean SBP * age group	
20-29	-0.011 (-0.023, 0.0012)
30-39	-0.0061 (-0.017, 0.0051)
40-49	-0.0024 (-0.012, 0.0070)
50-59	Reference
60-69	0.0069 (-0.0018, 0.015)
70-79	0.010 (0.0028, 0.018)
Mean DBP * age group	
20-29	0.029 (0.012, 0.046)
30-39	0.023 (0.0064, 0.038)
40-49	0.011 (-0.0038, 0.025)
50-59	Reference
60-69	-0.013 (-0.024, -6.7e-06)
70-79	-0.022 (-0.033, -0.011)
Mean SBP * decade	
2005-2015	Reference
1995-2004	0.0063 (-0.0046, 0.016)
1985-1994	0.0019 (-0.0076, 0.012)
Mean DBP * decade	
2005-2015	Reference
1995-2004	-0.0052 (-0.020, 0.010)
1985-1994	0.0014 (-0.014, 0.017)
Decade * age group	
2005-2015	Reference
1995-2004	
20-29	0.096 (-0.17, 0.36)
30-39	0.036 (-0.13, 0.20)
40-49	0.026 (-0.067, 0.12)
50-59	Reference
60-69	0.043 (-0.060, 0.15)
70-79	0.031 (-0.099, 0.16)
1985-1994	
20-29	-0.26 (-0.61, 0.088)
30-39	-0.0064 (-0.16, 0.15)
40-49	0.0042 (-0.082, 0.092)
50-59	Reference
60-69	-0.012 (-0.14, 0.11)
70-79	0.067 (-0.15, 0.28)
Mean SBP * decade * age group	
2005-2015	Reference

1995-2004	
20-29	0.011 (-0.0074, 0.029)
30-39	0.0021 (-0.015, 0.019)
40-49	-0.00055 (-0.017, 0.016)
50-59	Reference
60-69	-0.0098 (-0.025, 0.0055)
70-79	-0.0084 (-0.021, 0.0051)
1985-1994	
20-29	0.0063 (-0.015, 0.028)
30-39	0.0057 (-0.013, 0.024)
40-49	0.0018 (-0.014, 0.018)
50-59	Reference
60-69	0.0016 (-0.013, 0.017)
70-79	-0.0037 (-0.019, 0.012)
Mean DBP * decade * age group	
2005-2015	Reference
1995-2004	
20-29	-0.016 (-0.041, 0.0095)
30-39	-0.0063 (-0.030, 0.017)
40-49	0.0038 (-0.020, 0.027)
50-59	Reference
60-69	0.0055 (-0.015, 0.026)
70-79	0.0083 (-0.011, 0.027)
1985-1994	
20-29	-0.046 (-0.080, -0.010)
30-39	-0.017 (-0.042, 0.011)
40-49	-0.0071 (-0.031, 0.017)
50-59	Reference
60-69	-0.0047 (-0.029, 0.019)
70-79	0.0048 (-0.021, 0.030)

Traditional R^2 is not clearly defined for mixed effect models. The conditional R^2 for the model, which describes the proportion of variance explained by both fixed and random factors (1), is 0.918 (median of the results from the 1,000 simulated datasets).

SBP = systolic blood pressure. DBP = diastolic blood pressure.

Mean SBP is centred at 130 mm Hg, and mean DBP is centred at 80 mm Hg.

* denotes statistical interaction.

Supplementary Table 6. Coefficients of the regression of the probit-transformed prevalence of raised blood pressure on mean systolic and diastolic blood pressure for men.

Variable	Coefficient (95% CI)
Intercept	-0.49 (-0.52, -0.45)
Mean SBP (mm Hg)	0.025 (0.020, 0.031)
Mean DBP (mm Hg)	0.042 (0.033, 0.051)
Age group (years)	
20-29	-0.24 (-0.33, -0.17)
30-39	-0.16 (-0.21, -0.11)
40-49	-0.10 (-0.15, -0.060)
50-59	Reference
60-69	0.086 (0.036, 0.14)
70-79	0.15 (0.088, 0.22)
Decade	
2005-2015	Reference
1995-2004	0.00093 (-0.052, 0.054)
1985-1994	0.034 (-0.026, 0.093)
Mean SBP * age group	
20-29	0.013 (0.0035, 0.023)
30-39	0.0031 (-0.0055, 0.011)
40-49	-0.0011 (-0.0094, 0.0072)
50-59	Reference
60-69	0.0094 (-9.2e-5, 0.017)
70-79	0.012 (0.0045, 0.019)
Mean DBP * age group	
20-29	-0.012 (-0.026, 0.0016)
30-39	0.0062 (-0.0070, 0.019)
40-49	0.0074 (-0.0062, 0.021)
50-59	Reference
60-69	-0.015 (-0.028, -0.00067)
70-79	-0.021 (-0.033, -0.0091)
Mean SBP * decade	
2005-2015	Reference
1995-2004	0.0036 (-0.0080, 0.015)
1985-1994	0.0077 (-0.0025, 0.018)
Mean DBP * decade	
2005-2015	Reference
1995-2004	0.0041 (-0.014, 0.022)
1985-1994	-0.0015 (-0.017, 0.014)
Decade * age group	
2005-2015	Reference
1995-2004	
20-29	0.17 (0.016, 0.32)
30-39	0.070 (-0.023, 0.16)
40-49	0.046 (-0.039, 0.13)
50-59	Reference
60-69	0.082 (-0.021, 0.19)
70-79	0.053 (-0.079, 0.19)
1985-1994	
20-29	0.073 (-0.11, 0.26)
30-39	0.074 (-0.020, 0.17)
40-49	0.043 (-0.052, 0.14)
50-59	Reference
60-69	0.067 (-0.054, 0.19)
70-79	0.091 (-0.093, 0.28)
Mean SBP * decade * age group	
2005-2015	Reference

1995-2004	
20-29	0.0059 (-0.010, 0.022)
30-39	0.0014 (-0.014, 0.016)
40-49	0.0039 (-0.012, 0.020)
50-59	Reference
60-69	-0.0082 (-0.025, 0.0085)
70-79	-0.0050 (-0.019, 0.0096)
1985-1994	
20-29	0.0040 (-0.014, 0.021)
30-39	-0.0024 (-0.018, 0.013)
40-49	-0.0027 (-0.018, 0.013)
50-59	Reference
60-69	-0.010 (-0.027, 0.0064)
70-79	-0.013 (-0.029, 0.0028)
Mean DBP * decade * age group	
2005-2015	Reference
1995-2004	
20-29	-0.0011 (-0.025, 0.023)
30-39	-0.0032 (-0.027, 0.020)
40-49	-0.0041 (-0.030, 0.021)
50-59	Reference
60-69	0.00097 (-0.025, 0.027)
70-79	-0.0055 (-0.028, 0.018)
1985-1994	
20-29	-0.010 (-0.038, 0.018)
30-39	0.00022 (-0.022, 0.023)
40-49	0.00017 (-0.023, 0.024)
50-59	Reference
60-69	0.0047 (-0.022, 0.032)
70-79	0.0057 (-0.022, 0.033)

Traditional R^2 is not clearly defined for mixed effect models. The conditional R^2 for the model, which describes the proportion of variance explained by both fixed and random factors (1), is 0.871 (median of the results from the 1,000 simulated datasets).

SBP = systolic blood pressure. DBP = diastolic blood pressure.

Mean SBP is centred at 130 mm Hg, and mean DBP is centred at 80 mm Hg.

* denotes statistical interaction.

References

1. Nakagawa S, Schielzeth H. A general and simple method for obtaining R2 from generalized linear mixed-effects models. Methods Ecol Evol. 2013;4(2):133-42.
